# Synthesis of novel 5-alkyl/aryl/heteroaryl substituted diethyl 3,4-dihydro-2*H*-pyrrole-4,4-dicarboxylates by aziridine ring expansion of 2-[(aziridin-1-yl)-1-alkyl/aryl/heteroaryl-methylene]malonic acid diethyl esters

**DOI:** 10.3762/bjoc.7.95

**Published:** 2011-06-20

**Authors:** Satish S More, T Krishna Mohan, Y Sateesh Kumar, U K Syam Kumar, Navin B Patel

**Affiliations:** 1 Technology Development Centre, Custom Pharmaceutical Services, Dr. Reddy's Laboratories Ltd., Miyapur, Hyderabad -500 049, India; 2 Department of Chemistry, Veer Narmad South Gujarat University, Surat, India

**Keywords:** 5-alkyl/aryl/heteroaryl substituted 3,4-dihydro**-**2*H*-pyrrole-4,4-dicarboxylates, aziridine, *N*-vinyl substituted aziridines, ring expansion, sodium iodide

## Abstract

A novel synthetic methodology has been developed for the synthesis of diethyl 5-alkyl/aryl/heteroaryl substituted 3,4-dihydro-2*H*-pyrrole-4,4-dicarboxylates (also called 2-substituted pyrroline-4,5-dihydro-3,3-dicarboxylic acid diethyl esters) by iodide ion induced ring expansion of 2-[(aziridin-1-yl)-1-alkyl/aryl/heteroaryl-methylene]malonic acid diethyl esters in very good to excellent yields under mild reaction conditions. The electronic and steric impact of the substituents on the kinetics of ring expansion of *N*-vinyl aziridines to pyrrolines has been studied. Various diversely substituted novel pyrroline derivatives have been synthesized by this methodology and the products can be used as key intermediates in the synthesis of substituted pyrrolines, pyrroles and pyrrolidines.

## Introduction

Vinylaziridines are a particularly interesting class of aziridine derivatives that lend themselves to a host of highly useful synthetic transformations [1]. They are versatile electrophiles and notably undergo regioselective ring opening via addition at either the vinyl terminus [2–5] or directly at the aziridine ring carbon depending on the reagents employed [6–7]. Vinylaziridines have also been exploited in a variety of ring expansion reactions to afford a range of heterocyclic products, including piperidines [8–9], pyrrolines [10–14], imidazolidinones [15], β-lactams [16–18] and azepines [19–21].

There is ample evidence in the literature to confirm that the syntheses and applications of the *N*-acyl, *N*-sulfonamide or *N*-benzyl protected *C*-vinylaziridines are of considerable interest in organic chemistry and new applications of these compounds are being continuously explored [10–22]. However, not much attention has been paid to *N*-vinyl substituted aziridines. Thus, *N*-vinyl substituted aziridines provide an opportunity for researchers to explore their use for the development of novel synthetic methodologies and for various organic transformations. Recently, the ring opening of *N*-vinyl substituted aziridines by selenide nucleophiles to furnish functionalized open chain compounds has been described [23]. *N*-vinyl substituted aziridines can be converted into pyrrolines by ring expansion using various dipolarophiles [24–25] as well as by thermal ring expansion [26–27]. The literature also suggests that *N*-vinyl substituted aziridines can undergo iodide ion mediated ring expansion reactions to yield pyrroline derivatives [13–14]. However, iodide ion mediated reactions of *N*-vinyl substituted aziridines bearing an alkyl or aryl substituent on the α-carbon of the *N*-vinyl group did not yield pyrroline derivatives [28–29].

**Figure 1:**
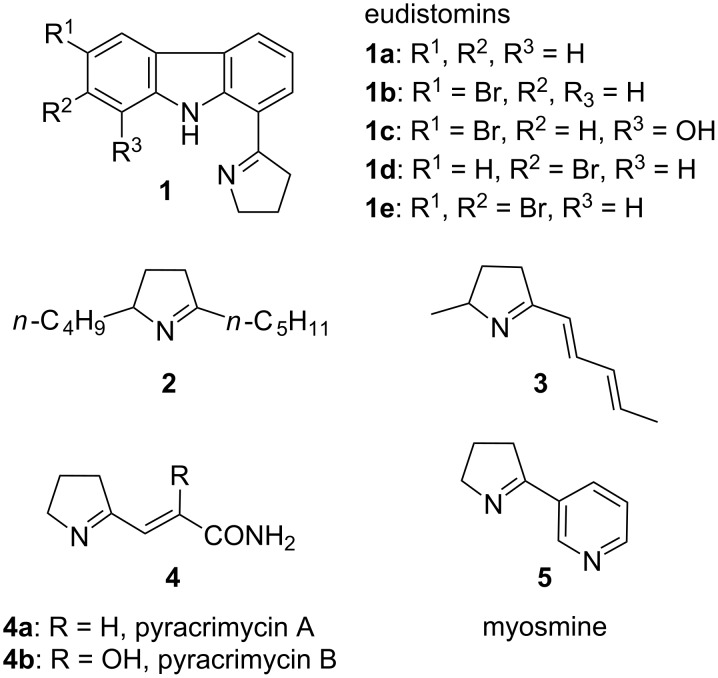
Natural products containing 2-substituted pyrroline residues in their core structural units.

**Figure 2:**
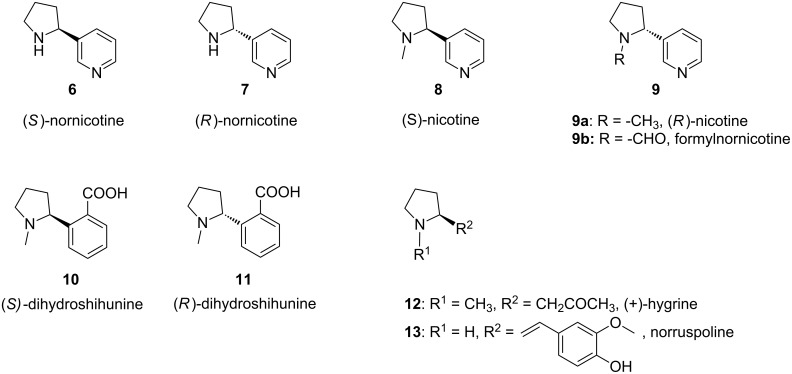
Natural products containing 2-substituted pyrrolidine residues in their core structural units.

2-Substituted pyrroline derivatives are important intermediates in organic synthesis and they can be used as the key starting materials in the preparation of substituted pyrroles, pyrrolines and pyrrolidines. The pyrrolines as well as pyrrolidines with substitution at the 2-position are important structural units in many natural products. A few of the natural products that contain 2-substituted pyrroline and 2-substituted pyrrolidine residues in their structural units are shown in Figure 1 and Figure 2, respectively.

## Results and Discussion

**Scheme 1:**
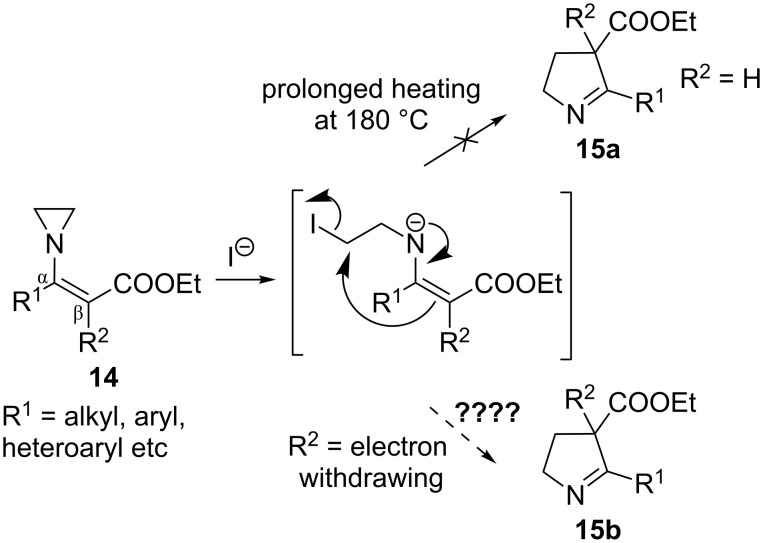
Iodide ion mediated ring expansion of *N-*vinylaziridines.

During the attempts of iodide ion mediated aziridine ring expansion reactions of *N*-vinyl substituted aziridines containing one electron withdrawing group on the β*-*carbon of the *N*-vinyl group and alkyl- or aryl groups on the α-carbon, the presumed intermediate formed upon ring opening of *N*-vinylaziridines by iodide ions did not result in a highly stabilized carbanion. Thus, reactions did not yield ring expansion products even at elevated reaction temperatures (above 180 °C), and instead reactions led to various by-products probably as a result of protonation and dehydrohalogenation of the presumed intermediates [28] (Scheme 1).

Junjappa and co-workers were successful in iodide ion mediated ring expansion of *N*-vinylaziridine *N*,*S*-acetals containing cyano- and ester groups on the β-carbon and obtained 2-thiomethylpyrroline derivatives [14,30]. This drew our attention to the synthesis of *N*-vinylaziridines containing multiple electron withdrawing groups on the β-carbon and alkyl- or aryl substituents on the α-carbon of the *N*-vinyl group, and to study the possibility of ring expansion to yield diversely substituted pyrrolines.

Thus, with the aim of broadening the scope of this *N*-vinylaziridine ring expansion for the synthesis of diversely substituted pyrrolines, we carried out iodide ion mediated ring expansion studies on *N*-vinyl substituted aziridines bearing multiple electron withdrawing substituents on the β-carbon of the *N*-vinyl group and various alkyl/aryl/heteroaryl substituents on the α-carbon of the *N*-vinyl group. Herein we report our successful attempts to overcome previously reported limitations of this methodology and propose a new approach for the synthesis of diethyl 5-alkyl/aryl/heteroaryl substituted 3,4-dihydro-2*H*-pyrrole-4,4-dicarboxylates.

**Scheme 2:**
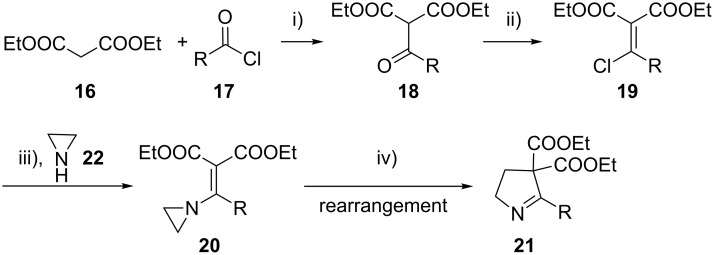
Synthesis of *N-*vinyl substituted aziridines and their ring expansion to pyrrolines. Reagents and conditions: i) MgCl_2_, CH_3_CN, TEA, 0 °C, then rt overnight; ii) POCl_3_, *n*-Bu_3_N, reflux; iii) THF, rt; iv) NaI, acetone, rt, overnight.

The synthesis of 2-[(aziridin-1-yl)-1-alkyl/aryl/heteroaryl-methylene]malonic acid diethyl esters was carried out by nucleophilic displacement by aziridine of the chloro atom from electron-poor activated 2-(1-alkyl/aryl/heteroaryl-1-chloromethylene)malonates **19**. The chloro alkenyl malonates **19** were synthesized in two steps from diethyl malonate and acid chlorides. The acylation of diethyl malonate was carried out with various acyl chlorides in the presence of anhydrous magnesium chloride and triethylamine, as per the reaction conditions developed by Rathke and Cowan [31], to give diethyl 2-acylmalonates **18**. The latter were then chlorinated using phosphorus oxychloride in the presence of tri *n*-butylamine at reflux, by the conditions reported by Hormi [32], to give 2-(1-alkyl/aryl/heteroaryl-1-chloromethylene)malonates **19**. The chloro displacement in **19** with aziridine (**22**) was carried out at room temperature with three equivalents of aziridine in THF and gave excellent yields of *N-*vinyl substituted aziridines **20** containing alkyl/aryl or heteroaryl substitution on the α-carbon and two ester groups on the β-carbon of *N*-vinylaziridine. The ring expansion reaction of *N*-vinyl substituted aziridines was performed under mild conditions by treatment of the aziridines with anhydrous sodium iodide in acetone at room temperature to give 5-alkyl/aryl/heteroaryl substituted 3,4-dihydro-2*H*-pyrrole-4,4-dicarboxylates **21** in very good to excellent yields (81–93%). The schematic representation of this methodology is shown in Scheme 2.

The details of the synthesis of various 2-[(aziridin-1-yl)-1-alkyl/aryl/heteroaryl-methylene]malonic acid diethyl esters are shown in Table 1.

**Table 1:** Synthesis of *N*-vinylaziridines from diethyl malonate and acyl chlorides.

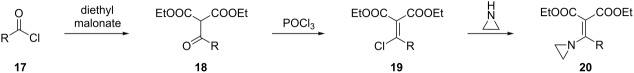						

Entry	Acid chloride **17**	**18**/Yield^a^	**19**/Yield^a^	Aziridine coupling with 2-(1-chloro alkenyl) malonates, **19**		

				Reaction time	Yield^a^	Product **20**

1	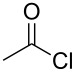 **17a**	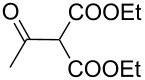 **18a**/80%	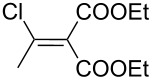 **19a**/65%	8 h	89%	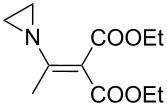 **20a**
2	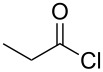 **17b**	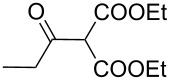 **18b**/85%	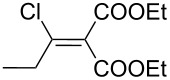 **19b**/70%	8 h	90%	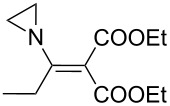 **20b**
3	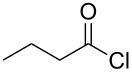 **17c**	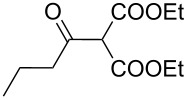 **18c**/75%	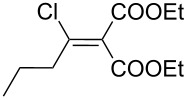 **19c**/70%	9 h	93%	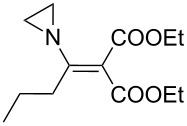 **20c**
4	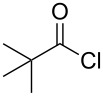 **17d**	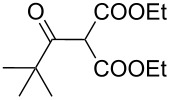 **18d**/90%	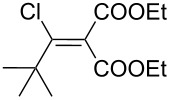 **19d**/69%	10 h	84%	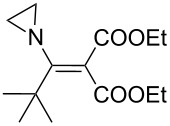 **20d**
5	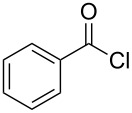 **17e**	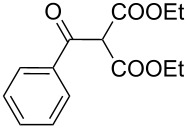 **18e**/85%	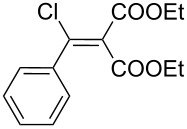 **19e**/81%	13 h	85%	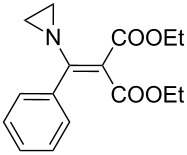 **20e**
6	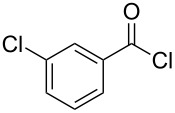 **17f**	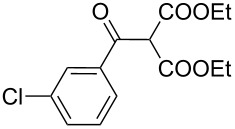 **18f**/89%	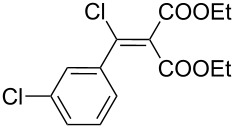 **19f**/76%	11 h	81%	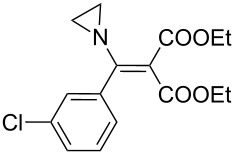 **20f**
7	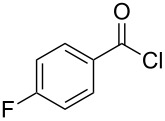 **17g**	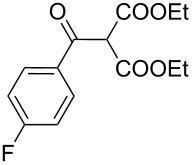 **18g**/78%	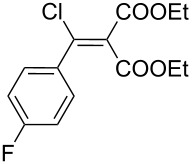 **19g**/70%	12 h	88%	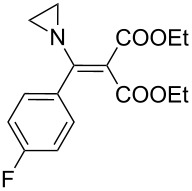 **20g**
8	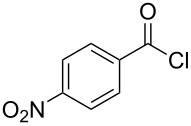 **17h**	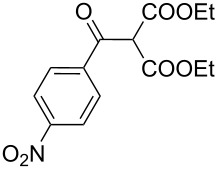 **18h**/79%	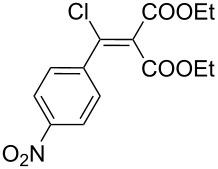 **19h**/77%	11 h	82%	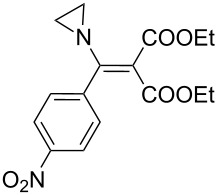 **20h**
9	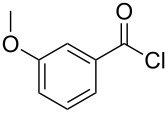 **17i**	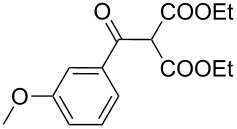 **18i**/85%	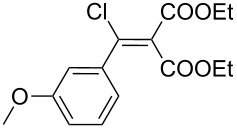 **19i**/65%	13 h	79%	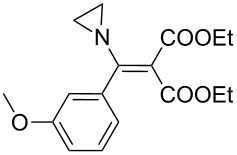 **20i**
10	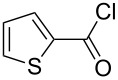 **17j**	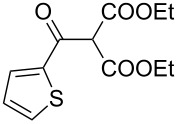 **18j**/81%	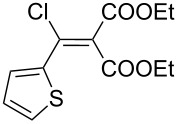 **19j**/70%	9 h	86%	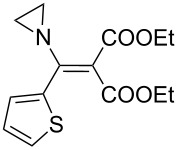 **20j**

^a^Isolated yields after column chromatography.

During the studies of iodide ion mediated ring expansion of *N*-vinylaziridines (having substitutions on the α-carbon of the vinyl group) to pyrrolines, it was observed that the reaction rates were slow in the case of aryl substitution at the α*-*carbon in comparison to alkyl substitution. This is probably due to steric hindrance encountered by the in situ generated methylene carbanion during the nucleophilic displacement of the iodo leaving group (Table 2, entries 1 and 5).

**Table 2:** Iodide ion mediated ring expansion of *N*-vinylaziridines.

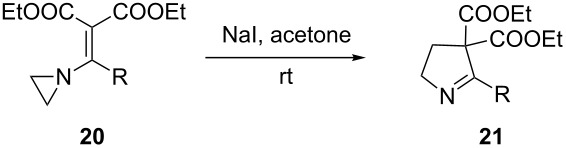				

Entry	Vinylaziridine **20**	Reaction time	Yield^a^	Pyrroline **21**

1	**20a**	12 h	90%	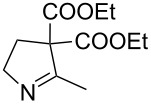 **21a**
2	**20b**	13 h	88%	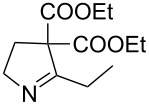 **21b**
3	**20c**	13 h	93%	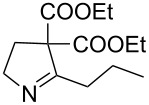 **21c**
4	**20d**	16 h	83%	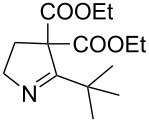 **21d**
5	**20e**	19 h	82%	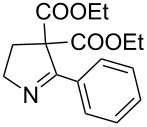 **21e**
6	**20f**	22 h	85%	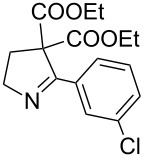 **21f**
7	**20g**	20 h	81%	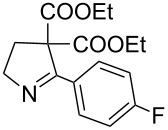 **21g**
8	**20h**	24 h	84%	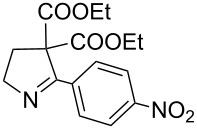 **21h**
9	**20i**	17.5 h	88%	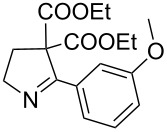 **21i**
10	**20j**	17h	90%	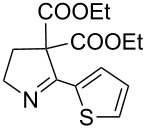 **21j**

^a^Isolated yields after purification.

It was also observed that in the presence of electron withdrawing groups on aryl rings, the rate of iodide ion mediated aziridine ring expansion to pyrrolines was slow compared to *N*-vinylaziridines bearing the aryl substituents with the electron donating group. The presence of the electron withdrawing group on the aryl ring at the α-carbon of vinylaziridines reduces the electron density on the methylene carbon of diethyl malonate and thus reduces the rate of iodo group displacement (Table 2, entries 5, 8 and 9).

**Scheme 3:**
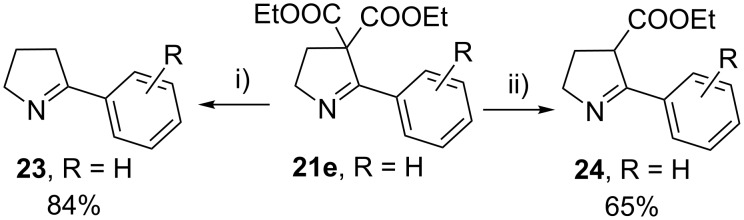
Hydrolytic decarboxylation. Reagents and conditions: i) DMSO, LiCl catalytic water, 140–150 °C, 3 h; ii) DMSO, LiCl, catalytic water, 100–110 °C, 12 h.

The hydrolytic decarboxylation of diethyl 3,4-dihydro-5-phenyl-2*H*-pyrrole-4,4-dicarboxylate (**21e**) was carried out in wet DMSO in the presence of lithium chloride at 140–150 °C (Krapcho’s method) for 3 h to give 2-phenylpyrroline (**23**) in about 84% yield. When this reaction was carried out at 100–110 °C for a period of 12 h, the mono ester **24** was isolated as the major product in 65% yield along with 10% of unreacted starting material and 10% of pyrroline derivative **23** (Scheme 3).

**Scheme 4:**
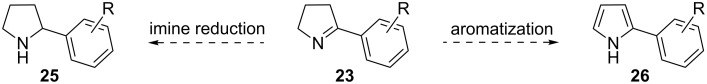
Reduction and aromatization of 2-substituted pyrrolines.

The imine bond reduction in pyrrolines to pyrrolidines can be carried out by established procedures in quantitative yields, either by reduction with sodium borohydride, or by catalytic hydrogenation using platinum on carbon [33–34]. The pyrrolines can be aromatized either by a two step procedure (i) NBS bromination and (ii) dehydrohalogenation in basic medium [35–37], or by dehydrogenation with Pd/C [38]. Thus, 2-substituted pyrrolines can be converted into biologically important pyrroles or pyrrolines having alkyl/aryl/heteroaryl substitution at the 2-position (Scheme 4).

**Scheme 5:**
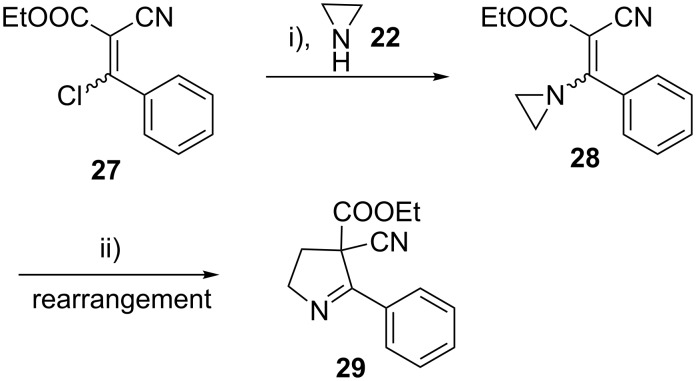
Reaction conditions: i) THF, rt, 3 h; ii) NaI, acetone, rt, overnight.

To see the generality of the aziridine ring expansion reaction with electron withdrawing groups other than ester, the *N*-vinylaziridine bearing one cyano group and one ester group, on the β*-*carbon of the *N*-vinyl group, was synthesized by the reaction of ethyl 3-chloro-2-cyano-3-phenylacrylate (**27**) with aziridine (**22**). The iodide ion mediated ring expansion of this vinylaziridine was carried out successfully to give the pyrroline derivative in 88% isolated yield (Scheme 5).

**Scheme 6:**
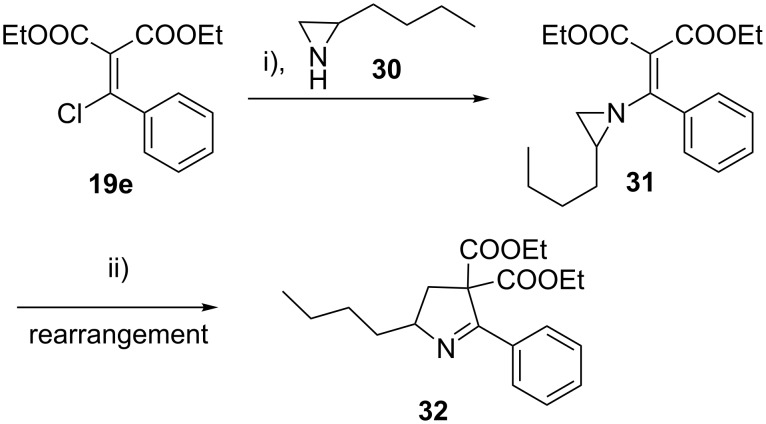
Reaction conditions: i) THF, TEA, rt; ii) NaI, acetone, rt, overnight.

Similarly, chloro displacement in β*-*chloro alkenyl malonate **19e** with 2-butylaziridine (**30**) gave 2-substituted *N*-vinylaziridine derivative **31**, which upon iodide ion mediated aziridine ring expansion gave the 2,5-disubstituted pyrroline derivative **32** in 82% isolated yield by regioselective aziridine ring opening and subsequent cyclization (Scheme 6).

## Conclusion

In conclusion, we have successfully demonstrated the iodide ion mediated aziridine ring expansion of *N-*vinylaziridines, bearing an alkyl or aryl/heteroaryl substitution on the α-carbon of the vinyl group, to afford novel 5-alkyl/aryl/heteroaryl substituted diethyl 3,4-dihydro-2*H*-pyrrole-4,4-dicarboxylates in good yields. The impact of the various electron donating and electron withdrawing substituents, as well as hindered and less bulky substituents on iodide ion mediated aziridine ring expansion, was also studied. Several novel diethyl 5-alkyl/aryl/heteroaryl-3,4-dihydro-2*H*-pyrrole-4,4-dicarboxylate derivatives were synthesized using this novel method. We also demonstrated the conversion of these diesters to the corresponding 2-substituted pyrrolines and 2-substituted-4,5-dihydro-3-carbethoxy-pyrroline derivatives which can be very useful synthetic intermediates for the synthesis of various pyrroline and pyrrolidine derivatives. The synthesis of some natural products using this approach is currently underway.

## Supporting Information

File 1 General information, experimental procedures, spectral data of compounds **18f**–**18j**, **19b**,**19c**, **19f**–**19g**, **19i**, **20a**–**20j**, **21a**–**21j**, **23**, **24**, **28**, **29**, **31**, **32**, spectra of **20a**, **20c**, **20d**, **20f**, **20g**, and **20h** (^1^H NMR, ^13^C NMR, IR, MS).

File 2 ^1^H NMR, ^13^C NMR, IR, and mass spectra of **21b**, **21d**, **21g**, **21h**, **21i**, **21j**, **23**, **24**, **28**, **29**, **31**, **32** and HRMS spectra of **21a**–**21j**, **23**, **24**, **29**, and **32**.
