# Supplementary material for: Synthesis of novel 5-alkyl/aryl/heteroaryl substituted diethyl 3,4-dihydro-2H-pyrrole-4,4-dicarboxylates by aziridine ring expansion of 2-[(aziridin-1-yl)-1-alkyl/aryl/heteroaryl-methylene]malonic acid diethyl esters
Source: Beilstein J Org Chem. 2011 Jun 20;7:831–8. doi: 10.3762/bjoc.7.95 (PMC3135249; doi:10.3762/bjoc.7.95)
Supplement: File 1 — General information, experimental procedures, spectral data of compounds 18f–18j, 19b,19c, 19f–19g, 19i, 20a–20j, 21a–21j, 23, 24, 28, 29, 31, 32, spectra of 20a, 20c, 20d, 20f, 20g, and 20h (1H NMR, 13C NMR, IR, MS). [file Beilstein_J_Org_Chem-07-831-s001.pdf]

Supporting Information

for

Synthesis of novel 5-alkyl/aryl/heteroaryl substituted diethyl 2*H*-pyrrole-4,4(3*H*)-dicarboxylates by aziridine ring expansion of 2-[(aziridin-1-yl)-1-alkyl/aryl/heteroaryl-methylene]malonic acid diethyl esters

Satish S. More*[1], T. Krishna Mohan[1], Y. Sateesh Kumar[1], U. K. Syam Kumar[1] and Navin B. Patel[2]

Address: [1]Technology Development centre, Custom Pharmaceutical Services, Dr. Reddy's Laboratories Ltd., Miyapur, Hyderabad -500 049, India and [2]Department of Chemistry, Veer Narmad South Gujarat University, Surat, India

Email: Satish S. More* - satishsm@drreddys.com

* Corresponding author

General information, experimental procedures, spectral data of compounds **18f–18j**, **19b**,**19c**, **19f–19g**, **19i**, **20a–20j**, **21a–21j**, **23**, **24**, **28**, **29**, **31**, **32**, spectra of **20a**, **20c**, **20d**, **20f**, **20g**, and **20h** (^{1}H NMR, ^{13}C NMR, IR, MS).

Table of contents

Content **Page**

1. General information S4
2. General procedures S5
   2.1. General procedure for preparation of *N*-vinylaziridines **20a–20j** S5
   2.2. General procedure for the iodide ion mediated ring expansion reactions of *N*-vinylaziridines S5
3. Spectral data of novel diethyl 2-acylmalonates S6
   3.1. Spectral data of Diethyl 2-(3-chlorobenzoyl)malonate (**18f**) S6
   3.2. Diethyl 2-(4-fluorobenzoyl)malonate (**18g**) S6
   3.3. Diethyl 2-(3-methoxybenzoyl)malonate (**18i**) S6
4. Spectral data of known diethyl 2-acylmalonates for which no spectral characterization was reported in literature S7
   4.1. Diethyl 2-(4-nitrobenzoyl)malonate (**18h**) S7
   4.2. Diethyl 2-(thiophene-2-carbonyl)malonate (**18j**) S7
5. Spectral data of novel 2-chloromethylenemalonates S7
   5.1. Diethyl 2-(chloro (3-chlorophenyl)methylene)malonate (**19f**) S7
   5.2. Diethyl 2-(chloro(4-fluorophenyl)methylene)malonate (**19g**) S8
   5.3. Diethyl 2-(chloro(3-methoxyphenyl)methylene)malonate (**19i**) S8
6. Spectral data of known 2-chloromethylenemalonic acid diethyl ester derivatives for which no spectral characterization was reported in literature S8
   6.1. Diethyl 2-(1-chloropropylidene)malonate (**19b**) S8
   6.2. Diethyl 2-(1-chlorobutylidene)malonate (**19c**) S8
7. Spectral data of 2-(aziridin-1-yl-1-alkyl/ aryl/heteroaryl methylene)malonates (**20a**–**20j**) S9
   7.1. 2-(1-Aziridin-1-yl-ethylidene)malonic acid diethyl ester (**20a**) S9
   7.2. 2-(1-Aziridin-1-yl-propylidene)malonic acid diethyl ester (**20b**) S9
   7.3. 2-(1-Aziridin-1-yl-butylidene)malonic acid diethyl ester (**20c**) S9
   7.4. 2-(1-Aziridin-1-yl-2,2-dimethyl-propylidene)malonic acid diethyl ester (**20d**) S10
   7.5. 2-(Aziridin-1-yl-phenylmethylene)malonic acid diethyl ester (**20e**) S10
   7.6. 2-[Aziridin-1-yl-(3-chlorophenyl)methylene]malonic acid diethyl ester (**20f**). S10
   7.7. 2-[Aziridin-1-yl-(4-fluorophenyl)methylene]malonic acid diethyl ester (**20g**) S10

7.8. 2-[Aziridin-1-yl-(4-nitrophenyl)methylene]malonic acid diethyl ester (**20h**) . S11
7.9. 2-[Aziridin-1-yl-(3-methoxyphenyl)methylene]malonic acid diethyl ester (**20i**)........ S11
7.10. 2-[(Aziridin-1-yl)-(thiophen-2-yl)methylene]malonic acid diethyl ester (**20j**) S11
8. Spectral data of diethyl 5-alkyl/aryl/heteroaryl substituted 3,4-dihydro-2*H*-pyrrole-4,4-dicarboxylates (**21a**–**21j**) S12
8.1. Diethyl 3,4-dihydro-5-methyl-2*H*-pyrrole-4,4-dicarboxylate (**21a**) S12
8.2. Diethyl 3,4-dihydro-5-ethyl-2*H*-pyrrole-4,4-dicarboxylate (**21b**) S12
8.3. Diethyl 3,4-dihydro-5-propyl-2*H*-pyrrole-4,4-dicarboxylate (**21c**) S12
8.4. Diethyl 3,4-dihydro-5-*tert*-butyl-2*H*-pyrrole-4,4-dicarboxylate (**21d**) S12
8.5. Diethyl 3,4-dihydro-5-phenyl-2*H*-pyrrole-4,4-dicarboxylate (**21e**) S13
8.6. Diethyl 5-(3-chlorophenyl)-3,4-dihydro-2*H*-pyrrole-4,4-dicarboxylate (**21f**) . S13
8.7. Diethyl 3,4-dihydro-5-(4-fluorophenyl)-2*H*-pyrrole-4,4-dicarboxylate (**21g**) . S13
8.8. Diethyl 3,4-dihydro-5-(4-nitrophenyl)-2*H*-pyrrole-4,4-dicarboxylate (**21h**) ... S14
8.9. Diethyl 3,4-dihydro-5-(3-methoxyphenyl)-2*H*-pyrrole-4,4-dicarboxylate (**21i**) S14
8.10. Diethyl 3,4-dihydro-5-(thiophen-2-yl)-2*H*-pyrrole-4,4-dicarboxylate (**21j**) S14
9. Spectral data of **23**, **24**, **28**, **29**, **30**, **31** and **32**. S15
9.1. 5-phenyl-3,4-dihydro-2*H*-pyrrole (**23**) S15
9.2. Ethyl 5-phenyl-3,4-dihydro-2*H*-pyrrole-4-carboxylate (**24**) S15
9.3. Ethyl 3-(aziridin-1-yl)-2-cyano-3-phenylacrylate (**28**) S15
9.4. Ethyl 4-cyano-5-phenyl-3,4-dihydro-2*H*-pyrrole-4-carboxylate (**29**) S15
9.5. 2-Butylaziridine (**30**) S16
9.6. Diethyl 2-[(2-butylaziridin-1-yl)phenylmethylene]malonate (**31**) S16
9.7. Diethyl 2-butyl-3,4-dihydro-5-phenyl-2*H*-pyrrole-4,4-dicarboxylate (**32**) S16
10. References S17
11. Spectra of 2-[(aziridin-1-yl)-1-alkyl/aryl/heteroaryl-methylene]malonic acid diethyl esters (*N*-vinyl aziridines) - **20a**, **20c**, **20d**, **20f**–**20h**........ S18

1. General information

All the required acid chlorides were freshly distilled prior to use. Aziridine was synthesized from ethanolamine and purified by fractional distillation. Laboratory grade (LR grade) solvents and reagents were used in the reactions. Reactions were monitored by TLC, using Merck aluminium-backed plates precoated with silica (0.25 mm, 60, F254). The plates were visualized under UV light and developed using a solution of basic $KMnO_4$. Chromatographic purification of products was carried out by gravity column chromatography on silica gel (60–120 mesh), purchased from SRL. Infrared spectra were recorded on a Perkin–Elmer 1650 Fourier transform spectrometer. NMR spectra were measured in $CDCl_3$, (all with TMS as internal standard) on Varian Gemini 200 MHz FT and 400 MHz FT magnetic resonance spectrometers. Chemical shifts (δ) are reported in ppm, and coupling constants (*J*) in Hz. The following abbreviations were used for multiplicities: s = singlet, d = doublet, t = triplet, q = quartet, m = multiplet. MS spectra were recorded on an HP-5989A quadrapole mass spectrometer.

The synthesis of diethyl acyl malonates **18** was carried by the method of Rathke and Cowan [1] and the physical and spectral data were compared with the literature values [1,2]. Compound **18h** [3] and **18j** [4] have been previously reported, however, since no spectral characterization was given, the spectral data were recorded and results reported below. Compound **18f**, **18g**, and **18i** were novel and were characterized by MS and NMR and IR spectroscopy.

The chlorination of diethyl 2-acylmalonates **18** was carried out by the method of Hormi [5] and the physical and spectral data of 2-(1-alkyl/aryl/heteroaryl-1-chloromethylene)malonates **19** were compared with the literature reports [6-8].

Compounds **19f**, **19g**, and **19i** were novel and were characterized by MS, NMR and IR spectroscopy. Compound **19b** and **19c** have been previously reported [9], however, since no spectral characterization was given, the spectral data were recorded and results reported below.

The synthesis of *N*-vinylaziridines **20** was carried out on a maximum of 24 mmol and minimum of 15 mmol scale whereas their rearrangement to pyrroline derivatives **21** was carried out on a maximum of 21 mmol and a minimum of 10 mmol scale.

Compound **23** was reported as perchlorate salt [10], but we isolated **23** in the form of a free base. Compound **24,** although reported in literature [11], was not completely characterized. We have carried out characterization of **24** by NMR and MS and HRMS and the spectral results of **24** were found to be similar to its methyl ester analogue [12].

The synthesis of ethyl 3-chloro-2-cyano-3-phenylacrylate (**27**) was carried out by a known procedure via the acylation of ethyl cyanoacetate with benzoyl chloride and subsequent chlorination of ethyl 2-benzoylcyanoacetate with phosphorus oxychloride [13]. The synthesis of 2-butylaziridine **30** was carried by the general procedure reported in a patent [14] from (±) norleucinol instead of (*S*)-(+)-leucinol.

2. General procedures

2.1. General procedure for preparation of *N*-vinylaziridines 20a–20j

The chloro alkenyl malonate derivative (16.1 mmol) and THF (40.0 mL) were placed in a round bottom flask and cooled to 0–10 °C. Aziridine (48.2 mmol) was added slowly over 15 minutes through a syringe to the above mixture. The reaction mixture was then raised to room temperature and stirred for 8–13 h. After disappearance of the starting chloro compound (TLC), the reaction was quenched with water (80 mL). The reaction mixture was extracted twice with 80 mL dichloromethane. The combined extracts were washed twice with 80 mL 10% sodium chloride solution. The organic layer was dried over Na_2SO_4 and concentrated under vacuum to afford the *N*-vinylaziridines. The products were sufficiently pure for the subsequent reactions; however, the crude products were purified by chromatography on silica gel (60–120 mesh) using a mixture of hexanes and ethyl acetate (90:10) as eluent, and the spectral data recorded for the column purified products, which were used for the next step (for yields see Table 1).

2.2. General procedure for the ring expansion of *N*-vinylaziridines to synthesize pyrrolines 21a–21j

Anhydrous sodium iodide (4.5 g, 30 mmol) was added to a solution of the *N*-vinylaziridine derivative (15 mmol) in acetone (40.0 mL) under a nitrogen atmosphere and the reaction mixture stirred for 12–24 h at room temperature. After disappearance of the *N*-vinylaziridine (TLC), the reaction mixture was diluted with water (80 mL) and extracted three times with 80 mL DCM. The combined DCM layers were washed twice with 80 mL of 10% sodium chloride solution, dried over Na_2SO_4 and concentrated under vacuum to afford the crude pyrroline derivatives which were purified by column chromatography on silica gel (60–120 mesh) with a mixture of hexanes and ethyl acetate (95:5) as eluent to afford the pure pyrrolines **21**.

3. Spectral data of novel diethyl acyl malonates

3.1. Diethyl 2-(3-chlorobenzoyl)malonate (18f)

M.F.: $C_{14}H_{15}ClO_5$, Mol. Wt: 298.72

IR (neat, cm^{-1}): 3651, 3070, 2984, 1754, 1734, 1698, 1571, 1424, 1369, 1301, 1249, 1151, 1095, 1031, 797, 744, 682, 616; ^{1}H NMR ($CDCl_3$, 400 MHz) δ: 13.4 and 5.21 (s, 1H), 7.88 (s, 1H), 7.769–7.762 (m, 1H), 7.57–7.55 (m, 1H), 4.29–4.0 (m, 4H), 1.25 and 1.05 (t, J = 7.2 Hz, 6H); ^{13}C NMR ($CDCl_3$, 50 MHz) δ: 187 and 173, 164.39, 136.9, 134.3, 133.3, 130.11, 128.49, 126.48, 125.7 and 61.39, 62.52 and 61.84, 14.06 and 13.88; MS (ESI): m/z = 299.1 $[M + H]^+$.

3.2. Diethyl 2-(4-fluorobenzoyl)malonate (18g)

M.F.: $C_{14}H_{15}FO_5$, Mol. Wt: 282.26

IR (neat, cm^{-1}): 3070, 2990, 2876, 1751, 1733, 1691, 1594, 1508, 1478, 1447, 1413, 1371, 1296, 1230, 1185, 1160, 1034, 1006, 907, 852, 817, 635, 580; ^{1}H NMR ($CDCl_3$, 400 MHz) δ: 7.96–7.92 (m, 2H), 7.16 (t, J = 8.6 Hz, 2H), 13.4 & 5.22 (s, 1H), 4.28 (q, J = 7.0 Hz, 4H), 1.25 (t, J = 7.4 Hz, 6H); ^{13}C NMR ($CDCl_3$, 50 MHz) δ: 187.3, 168.7, 164.6 & 163.5, 131.7, 131.3 & 131.1, 116.3 & 115.8, 62.48 & 61.88, 61.7 & 61.3, 13.9, MS (ESI): m/z = 283.1 $[M + H]^+$.

3.3. Diethyl 2-(3-methoxybenzoyl)malonate (18i)

M.F.: $C_{15}H_{18}O_6$, Mol. Wt: 294.30

IR (neat, cm^{-1}): 3077, 2983, 2839, 1754, 1736, 1693, 1598, 1583, 1487, 1450, 1431, 1369, 1293, 1234, 1178, 1095, 1037, 868, 789, 686; ^{1}H NMR ($CDCl_3$, 400 MHz) δ: 13.4 and 5.26 (s, 1H), 7.47–7.0 (m, 4H), 4.27 (q, J = 7.0 Hz, 4H), 3.85 (s, 3H), 1.25 (t, 6H, 7.2 Hz); ^{13}C NMR ($CDCl_3$, 100 MHz) δ: 171.6, 166.6, 159.5, 130.6, 129.4, 122.5, 120.3, 114.4, 61.4, 55.3, 41.6, 13.9 MS (ESI): m/z = 295.2 $[M + H]^+$.

4. Spectral data of known diethyl 2-acylmalonates for which no spectral characterization was reported before in literature

4.1. Diethyl 2-(4-nitrobenzoyl)malonate (18h)

M.F.: $C_{14}H_{15}NO_7$, Mol. Wt: 309.27

IR (neat, cm^{-1}): 3112, 2985, 2874, 1754, 1732, 1701, 1649, 1605, 1588, 1529, 1466, 1370, 1348, 1297, 1252, 1147, 1084, 1036, 855, 767, 687; ^{1}H NMR ($CDCl_3$, 400 MHz) δ : 13.4 and 5.5 (s,s, 1H), 8.34 and 8.27 (d, *J* = 8.8 Hz, and d, *J* = 8.8 Hz, 2H), 8.08 and 7.75 (d, *J* = 7.2 Hz, and d, *J* = 8.8 Hz, 2H), 4.39–4.07 (m, 4H), 1.08–1.38 (m, 6H); ^{13}C NMR ($CDCl_3$, 50 MHz) δ: 187.6 and 164.1, 172.2 and 170.5, 166.5 and 165.2, 150.6 and 149.0, 139.8 and 139.8, 129.5 and 128.7, 124.0 and 123.4, 101.9 and 41.6, 62.7 and 62.1, 61.5 and 61.4, 14.0 and 13.9, MS (ESI): *m*/*z* = 310.1 $[M + H]^+$.

4.2. Diethyl 2-(thiophene-2-carbonyl)malonate (18j)

M.F.: $C_{12}H_{14}O_5S$, Mol. Wt: 270.30

IR (neat, cm^{-1}): 3460, 3106, 2985, 1735, 1670, 1519, 1446, 1413, 1305, 1245, 1179, 1035, 854, 736, 616; ^{1}HNMR ($CDCl_3$, 400 MHz) δ: 13.26 and 5.14 (s, s, 1H), 7.69–7.73 (m, 2H), 7.14 (dd, *J*=3.8 Hz, *J*= 5.0 Hz, 1H), 4.18–4.32 (m, 4H), 1.24–1.32 (m, 6H); ^{13}C NMR ($CDCl_3$, 50 MHz) δ: 181.1, 164.2, 142.2, 135.3, 133.1, 128.3, 62.46 and 62.40, 13.8; MS (ESI): *m*/*z* = 271.1 $[M + H]^+$.

5. Spectral data of novel diethyl 2-chloromethylenemalonates

5.1. Diethyl 2-(chloro(3-chlorophenyl)methylene)malonate (19f)

M.F.: $C_{14}H_{14}Cl_2O_4$, Mol Wt: 316.03

IR (neat): 3454; 3067, 2983, 1732, 1621, 1567, 1472, 1446, 1390, 1367, 1249, 1208, 1079, 1019, 935, 864, 788, 717, 690; ^{1}H NMR (400 MHz, $CDCl_3$) δ: 7.42–7.29 (m, 4H ArH), 4.35 (q, *J* = 7.2 Hz, 2H), 4.09 (q, *J* = 6.9 Hz, 2H), 1.36 (t, *J* = 7.6, 3H), 1.07 (t, *J* = 8.0Hz, 3H); ^{13}C NMR (50 MHz, $CDCl_3$) δ: 163.0, 162.3, 145.3, 138.3, 134.1, 130.2, 129.4, 128.1, 127.8, 126.2, 62.1, 14.0; MS (ESI): *m*/*z* = 339.0 $[M + Na]^+$.

5.2. Diethyl 2-(chloro(4-fluorophenyl)methylene)malonate (19g)

M.F.: $C_{14}H_{14}ClFO_4$, Mol. Wt: 300.71.

IR (neat): 3452; 3109, 2985, 1732, 1601, 1507, 1368, 1301, 1253, 1227, 1160, 1079, 1015, 908, 841. ^{1}H NMR (400 MHz, $CDCl_3$) δ: 7.45–7.40 (m, 2H), 7.10–7.05 (m, 2H), 4.35 (q, *J* = 7.0 Hz, 2H), 4.06 (q, *J* = 7.3 Hz, 2H), 1.36 (t, *J* = 7.0 Hz, 3H), 1.08 (t, *J* = 7.2 Hz, 3H); ^{13}C NMR (50 MHz, $CDCl_3$) δ: 166.1, 163.1, 162.5 & 161.1, 146.1, 132.8 & 130.4, 127.2, 115.5, & 115.11, 61.9 & 61.7, 13.9 & 13.6; MS (ESI): *m*/*z* = 301.0 $[M + H]^+$.

5.3. Diethyl 2-(chloro(3-methoxyphenyl)methylene)malonate (19i)

M.F.: $C_{15}H_{17}ClO_5$, Mol. Wt: 312.75

IR (neat): 3453, 3071, 2983, 1732, 1597, 1485, 1465, 1390, 1368, 1290, 1228, 1174, 1164, 1078, 1039, 1023, 949, 921, 865, 788, 761, 695; ^{1}H NMR (400 MHz, $CDCl_3$) δ: 7.30–7.27(d, *J* = 3.2 Hz, 1H), 7.01–6.93 (m, 2H), 4.35 (q, *J* = 7.3 Hz, 2H), 4.07 (q, *J* = 7.2 Hz, 2H), 3.8 (s, 3H), 1.36 (t, *J* = 7.0 Hz, 3H), 1.05 (t, *J* = 6.8 Hz, 3H);^{13}C NMR (50 MHz, $CDCl_3$) δ:163.2, 162.8, 159.2, 146.9, 138.0, 129.2, 127.1, 120.3, 116.2, 113.3, 61.9, 61.7, 55.3, 14.0, 13.6; MS (ESI): *m*/*z* = 335.1 $[M + Na]^+$.

6. Spectral data of known 2-chloromethylenemalonic acid diethyl ester derivatives for which no spectral characterization was reported in literature

6.1. Diethyl 2-(1-chloropropylidene)malonate (19b)

M.F.: $C_{10}H_{15}ClO_4$, Mol. Wt: 234.68

IR (neat): 2982, 2940, 1727, 1626, 1461, 1389, 1367, 1286, 1258, 1230, 1044, 1062, 905, 866, 755, 667; ^{1}H NMR (400 MHz, $CDCl_3$) δ: 4.32 (q, *J* = 6.8 Hz, 2H), 4.24 (q, *J* = 6.4 Hz, 2H), 2.92 (q, *J* = 7.2 Hz, 2H), 1.33 (t, *J* = 7.4 Hz, 3H) 1.29 (t, *J* = 7.2 Hz, 3H), 1.23 (t, *J* = 7.4 Hz, 3H);^{13}C NMR (50 MHz, $CDCl_3$) δ: 164.1, 162.0, 155.7, 125.8, 61.6 and 61.5, 30.4, 13.9, 12.0;MS (ESI): *m*/*z* = 235.1 $[M + H]^+$.

6.2. Diethyl 2-(1-chlorobutylidene)malonate (19c)

M.F.: $C_{11}H_{17}ClO_4$, Mol. Wt: 248.70

IR (neat): 3441, 2967, 2875, 1735, 1625, 1465, 1388, 1367, 1274, 1245, 1223, 1141, 1086, 1055, 1022, 921, 865, 759, 665; ^{1}H NMR (400 MHz, $CDCl_3$) δ: 4.30 (q, *J* = 7.0 Hz,

2H), 4.22 (q, J = 7.2 Hz, 2H), 2.88-2.92 (m, 2H), 1.69–1.75 (m, 2H), 1.27–1.34 (m, 6H), 0.98 (t, J = 7.4 Hz, 3H); ^{13}C NMR (50 MHz, $CDCl_3$) δ: 164.2, 162.1, 154.3, 126.6, 61.6, 38.4, 21.0, 13.9; MS (ESI): m/z = 271.1 $[M + Na]^+$.

7. Spectral data of 2-(aziridin-1-yl-1-alkyl/aryl/heteroaryl methylene)malonates (20a–20j)

7.1. 2-(1-Aziridin-1-yl-ethylidene)malonic acid diethyl ester (20a)

M.F.: $C_{11}H_{17}NO_4$, Mol. Wt: 227.26

IR (neat): 2981, 1704, 1646, 1591, 1446, 1381, 1225, 1182, 1142, 1061, 973, 868, 773; ^{1}H NMR ($CDCl_3$, 400 MHz) δ: 4.27 (q, J = 7.2 Hz, 2H), 4.19 (q, J = 7.06 Hz, 2H), 2.24 (s, 3H), 2.17 (s, 4H), 1.26 (t, J = 7.2 Hz, 3H), 1.22 (t, J = 7.2 Hz, 3H); ^{13}C NMR ($CDCl_3$, 50 MHz) δ: 165.9, 165.3, 109.9, 60.6, 60.3, 28.8, 20.0, 14.1; MS (ESI): m/z = 228.1 $[M + H]^+$.

7.2. 2-(1-Aziridin-1-yl-propylidene)malonic acid diethyl ester (20b)

M.F.: $C_{12}H_{19}NO_4$, Mol. Wt: 241.28

IR (neat, cm^{-1}): 3405, 2981, 2939, 1707, 1587, 1464, 1383, 1367, 1260, 1221, 1179, 1142, 1095, 1065, 1034, 941, 814, 676; ^{1}H NMR ($CDCl_3$, 400 MHz) δ: 4.26 (q, J = 7.2 Hz, 2H), 4.20 (q, J = 7.0 Hz, 2H), 2.56 (dd, J = 6.0, 7.8 Hz, 2H), 2.18 (s, 4H), 1.2–1.4 (m, 9H); ^{13}C NMR ($CDCl_3$, 50 MHz) δ: 169.7, 165.9, 109.1, 60.6, 60.4, 28.3, 26.8, 14.2, 14.1, 12.9; MS (ESI): m/z = 242.2 $[M + H]^+$.

7.3. 2-(1-Aziridin-1-yl-butylidene)malonic acid diethyl ester (20c)

M.F.: $C_{13}H_{21}NO_4$, Mol. Wt: 255.31

IR (neat, cm^{-1}): 3070, 2978, 2874, 1705, 1586, 1464, 1378, 1241, 1218, 1178, 1141, 1096, 1063, 1039, 985, 868, 811, 756, 667; ^{1}H NMR ($CDCl_3$, 400 MHz) δ: 4.28 (q, J = 7.0 Hz, 2H), 4.20 (q, J = 7.0 Hz, 2H), 2.58–2.54 (m, 2H), 2.18 (s, 4H), 1.69–1.65 (m, 2H), 1.30 (t, J = 7.6 Hz, 3H), 1.27 (t, J = 7.2 Hz, 3H), 0.989 (t, J = 7.4 Hz, 3H); ^{13}C NMR ($CDCl_3$, 50 MHz) δ: 168.4, 166.0, 165.8, 109.6, 60.5, 60.4, 35.2, 28.5, 21.9, 14.17, 14.11, 14.0; MS (ESI): m/z = 256.2 $[M + H]^+$, 278.2 $[M + Na]^+$.

7.4. 2-(1-Aziridin-1-yl-2,2-dimethylpropylidene)malonic acid diethyl ester (20d)

M.F.: $C_{14}H_{23}NO_4$, Mol. Wt: 269.34

IR (neat, cm^{-1}): 3069, 2979, 2874, 1712, 1557, 1471, 1399, 1365, 1260, 1224, 1198, 1145, 1095, 1059, 957, 869, 818, 772, 692; ^{1}H NMR ($CDCl_3$, 400 MHz) δ: 4.21 (q, *J* = 7.0 Hz, 4H), 2.18 (s, 4H), 1.34(s, 9H), 1.27 (t, *J* = 7.2 Hz, 6H); ^{13}C NMR ($CDCl_3$, 50 MHz) δ: 173.8, 166.6, 111.1, 60.6, 38.5, 30.6, 29.7, 13.9; MS (ESI): *m*/*z* = 270 $[M + H]^+$, 292.2 $[M + Na]^+$.

7.5. 2-(Aziridin-1-yl-phenylmethylene)malonic acid diethyl ester (20e)

M.F.: $C_{16}H_{19}NO_4$, Mol. Wt: 289.34

IR (neat, cm^{-1}): 3061, 2981, 2902, 1708, 1570, 1489, 1469, 1444, 1369, 1280, 1240, 1205, 1143, 1089, 1053, 939, 920, 860, 759, 700, 624; ^{1}H NMR ($CDCl_3$, 400 MHz) δ: 7.41–7.32 (m, 5H), 4.32–4.26 (m, 2H), 3.95–3.90 (m, 2H), 2.21 (s, 4H), 1.33 (t, *J* = 7.2 Hz, 3H) 0.94 (t, *J* = 7.2 Hz, 3H); ^{13}C NMR ($CDCl_3$, 100 MHz) δ: 165.9, 165.7, 164.13, 137.3, 128.8, 127.9, 127.2, 110.7, 60.6 and 60.58, 31.1, 14.1,13.5 MS (ESI): *m*/*z* = 290.2 $[M + H]^+$, 312.2 $[M + Na]^+$.

7.6. 2-[Aziridin-1-yl-(3-chlorophenyl)methylene]malonic acid diethyl ester (20f)

M.F.: $C_{16}H_{18}ClNO_4$, Mol. Wt: 323.77

IR (neat, cm^{-1}): 3423, 3067, 2981, 1715, 1602, 1581, 1473, 1413, 1370, 1284, 1240, 1207, 1145, 1091, 1055, 891, 864, 804, 784;^{1}H NMR ($CDCl_3$, 400 MHz) δ: 7.36–7.21 (m, 4H, ArH), 4.29 (q, *J* = 7.2 Hz, 2H), 3.98 (q, *J* = 7.0 Hz, 2H), 2.21 (s, 4H), 1.32 (t, *J* = 3.8 Hz, 3H), 1.01 (t, *J* = 7.0 Hz, 3H);^{13}C NMR ($CDCl_3$, 50 MHz) δ: 165.5, 164.1, 163.5, 138.9, 134.0, 129.4, 129.0, 127.5, 125.6, 116.1, 111.3, 60.8, 31.0, 30.1, 14.2, 13.68; MS (ESI): *m*/*z* = 346.1 $[M + Na]^+$.

7.7. 2-[Aziridin-1-yl-(4-fluorophenyl)methylene]malonic acid diethyl ester (20g)

M.F.: $C_{16}H_{18}FNO_4$, Mol. Wt: 307.32

IR (neat, cm^{-1}): 3073, 2938, 2874, 1715, 1605, 1579, 1507, 1474, 1370, 1277, 1207, 1145, 1089, 1054, 934, 864, 841, 789;^{1}H NMR ($CDCl_3$, 400 MHz) δ: 7.36–7.32 (m, 2H), 7.06–7.03 (m, 2H), 4.30 (q, *J* = 7.2 Hz, 2H), 3.97 (q, *J* = 7.0 Hz, 2H), 2.20 (s, 4H), 1.33 (t, *J* = 7.4 Hz, 3H), 1.01 (t, *J* = 7.2 Hz, 3H); ^{13}C NMR ($CDCl_3$, 50 MHz) δ: 165.8, 165.4 & 164.3, 160.4, 133.3, 129.5 & 129.4, 115.4 & 114.9, 111.1, 60.8, 31.1, 14.2, 13.7; MS (ESI): *m*/*z* = 308.2 $[M + H]^+$.

7.8. 2-[Aziridin-1-yl-(4-nitrophenyl)methylene]malonic acid diethyl ester (20h)

M.F.: $C_{16}H_{18}N_2O_6$, Mol. Wt: 334.32

IR (neat, cm^{-1}): 3077, 2983, 2873, 1714, 1604, 1581, 1523, 1347, 1279, 1241, 1208, 1145, 1090, 1055, 857, 745, 700;^{1}H NMR ($CDCl_3$, 400 MHz) δ: 8.26 (dt, *J*= 2.0 Hz, *J*= 8.8 Hz, 2H), 7.52 (dt, *J*= 2.0 Hz, *J*= 8.8 Hz, 2H), 4.34 (q, *J* = 7.0Hz, 2H), 3.99 (q, *J* = 7.0 Hz, 2H), 2.20 (s, 3H), 1.36 (t, *J* = 7.0 Hz, 3H), 1.03 (t, *J* = 7.0 Hz, 3H); ^{13}C NMR ($CDCl_3$, 50 MHz) δ: 164.8, 164.4, 162.4, 147.9, 143.5, 128.7, 123.4, 111.8, 61.2, 61.0, 30.6, 29.7, 14.2, 13.8, MS (ESI): *m*/*z* = 335.1 [M + 1] , 357.1 $[M + Na]^+$.

7.9. 2-[Aziridin-1-yl-(3-methoxyphenyl)methylene]malonic acid diethyl ester (20i)

M.F.: $C_{17}H_{21}NO_5$, Mol. Wt: 319.35

IR (neat, cm^{-1}): 3072, 2981, 2938, 2837, 1714, 1574, 1465, 1370, 1290, 1177, 1143, 1093, 1053, 869, 787, 692; ^{1}H NMR ($CDCl_3$, 400 MHz) δ: 7.28–7.24 (m, 1H), 6.92–6.89 (m, 3H), 4.30 (q, *J* = 7.2 Hz, 2H), 3.96 (q, *J* = 7.2 Hz, 2H), 3.79 (s, 3H), 2.22 (s, 3H), 1.33 (t, *J* = 7.2 Hz, 3H), 0.98 (t, *J* = 7.4 Hz, 3H); ^{13}C NMR ($CDCl_3$, 100 MHz) δ: 166.0, 165.4, 164.0, 159.2, 138.6, 129.1, 119.6, 114.88, 112.5, 110.8, 60.7, 60.6, 60.1, 55.2, 31.3, 14.1, 13.6; MS (ESI): *m*/*z* = 320.2 $[M + H]^+$.

7.10. 2-[(Aziridin-1-yl)-(thiophen-2-yl)methylene]malonic acid diethyl ester (20j)

M.F.: $C_{14}H_{17}NO_4S$, Mol. Wt: 295.35

IR (neat, cm^{-1}): 3637, 3103, 2981, 2610, 1710, 1574, 1370, 1278, 1221, 1144, 1052, 859, 713; ^{1}H NMR ($CDCl_3$, 400 MHz) δ: 7.42 (dd, *J* = 1.2, *J* = 5.2 Hz, 1H), 7.20 (dd, *J* = 1.0, *J* = 3.6 Hz, 1H), 6.98 (dd, *J* = 3.6, 5.2 Hz, 1H), 4.28 (q, *J* = 7.2 Hz 2H), 4.07 (q, *J* = 7.0 Hz, 2H), 2.29 (s, 4H), 1.31 (t, *J* = 7.0 Hz, 3H), 1.10 (t, *J* = 7.0 Hz, 3H); ^{13}C NMR ($CDCl_3$, 50 MHz) δ: 166.3, 163.4, 158.3, 137.8, 128.3, 127.9, 126.4, 111.3, 61.1, 60.6, 32.4, 14.1, 13.7; MS (ESI): *m*/*z* = 296.1 $[M + H]^+$, 318.1 $[M + Na]^+$.

8. Spectral data of diethyl 5-alkyl/aryl/heteroaryl substituted 3,4-dihydro-2*H*-pyrrole-4,4-dicarboxylates (21a–21j)

8.1. Diethyl 3,4-dihydro-5-methyl-2*H*-pyrrole-4,4-dicarboxylate (21a)

M.F.: $C_{11}H_{17}NO_4$, Mol. Wt: 227.26

IR (neat): 2982, 2936, 2874, 1731, 1651, 1595, 1446, 1367, 1263, 1178, 1093, 1060, 1023, 973, 927, 861, 796; ^{1}H NMR ($CDCl_3$, 400 MHz) δ: 4.25 (q, *J* = 7.0 Hz, 4H), 3.87–3.83 (m, 2H), 2.56 (t, *J* = 6.8 Hz, 2H), 2.20 (s, 3H), 1.29 (t, *J* = 7.0 Hz, 6H); ^{13}C NMR ($CDCl_3$, 50 MHz) δ: 168.4, 168.0, 71.3, 61.9, 58.8, 33.7, 18.0, 13.9; MS (ESI): *m*/*z* = 228 $[M + H]^+$, 246 $[M + Na]^+$; HRMS calculated for [$C_{11}H_{17}NO_4$ + H]$^+$: 228.36, found 228.1231.

8.2. Diethyl 3,4-dihydro-5-ethyl-2*H*-pyrrole-4,4-dicarboxylate (21b)

M.F.: $C1_2H_{19}NO_4$, Mol. Wt: 241.28

IR (neat, cm^{-1}): 3407, 2981, 2940, 1731, 1646, 1678, 1463, 1447, 1367, 1267, 1179, 1098, 991, 861, 666; ^{1}H NMR ($CDCl_3$, 400 MHz) δ: 4.22 (q, *J* = 7.2 Hz, 4H), 3.88 (t, *J* = 2.2 Hz, 2H), 2.57–2.48 (m, 4H), 1.28 (t, *J* = 6.8 Hz, 6H), 1.20 (t, *J* = 7.2 Hz, 3H); ^{13}C NMR ($CDCl_3$, 50 MHz) δ: 172.3, 168.6, 71.4, 61.9, 58.8, 33.9, 24.7, 13.9, 10.7; MS (ESI): *m*/*z* = 242 $[M + H]^+$; HRMS calculated for [$C_{12}H_{19}NO_4$ + H]$^+$: 242.1392, found 242.1388.

8.3. Diethyl 3,4-dihydro-5-propyl-2*H*-pyrrole-4,4-dicarboxylate (21c)

M.F.: $C_{13}H_{21}NO_4$, Mol. Wt: 255.31

IR (neat, cm^{-1}): 3393, 3303, 3079, 2966, 2875, 1731, 1648, 1545, 1445, 1370, 1218, 1179, 1157, 1096, 1026, 861, 756, 666; ^{1}H NMR ($CDCl_3$, 400 MHz) δ: 4.24 (q, *J* = 7.2 Hz, 4H), 3.91–3.86 (m, 2H), 2.54 (t, *J* = 7.2 Hz, 2H), 2.48–2.43 (m, 2H), 1.71 (q, *J* = 7.4 Hz, 2H), 1.29 (t, *J* = 7.2 Hz, 6H), 0.96 (t, *J* = 7.4 Hz, 3H); ^{13}C NMR ($CDCl_3$, 100 MHz) δ: 171.1, 168.6, 71.5, 61.7, 58.9, 35.1, 33.6, 33.3, 28.4, 19.6, 13.9, 13.8, 13.7, MS (ESI): *m*/*z* = 256 $[M + H]^+$; HRMS calculated for [$C_{13}H_{21}NO_4$ + H]$^+$: 256.1549, found 256.1551.

8.4. Diethyl 3,4-dihydro-5-*tert*-butyl-2*H*-pyrrole-4,4-dicarboxylate (21d)

M.F.: $C_{14}H_{23}NO_4$, Mol. Wt: 269.34

IR (neat, cm^{-1}): 3445.84, 2981.42, 2871.32, 1731.88, 1622.02, 1481.34, 1463.83, 1393.26, 1365.44, 1304.88, 1260.48, 1177.68, 1084.71, 1025.92, 999.27, 963.15, 864.89 and 772.29.^{1}H NMR ($CDCl_3$, 400 MHz) δ: 4.24 (q, *J* = 7.2 Hz, 4H), 3.85 (t, *J* = 6.6

Hz, 2H), 2.59 (t, *J* = 6.8 2H), 1.30 (t, *J* = 7.0 Hz, 3H), 1.28 (t, *J* = 6.8 Hz, 6H), 1.25 (s, 9H); ^{13}C NMR ($CDCl_3$, 50 MHz) δ: 178.5, 169.3, 70.0, 61.7, 58.1, 37.9, 37.7, 29.6, 13.8; MS (ESI): *m*/*z* = 270 $[M + H]^+$; HRMS calculated for $[C_{14}H_{23}NO_4 + H]^+$: 270.1705, found 270.1703.

8.5. Diethyl 3,4-dihydro-5-phenyl-2*H*-pyrrole-4,4-dicarboxylate (21e)

M.F.: $C_{16}H_{19}NO_4$, Mol. Wt: 289.33

IR (neat, cm^{-1}): 3419, 2981, 1732, 1446, 1261, 1178, 1085, 1018, 759, 694; ^{1}H NMR ($CDCl_3$, 400 MHz) δ: 7.87–7.85 (m, 2H), 7.39–7.32 (m, 3H), 4.23–4.15 (m, 4H), 4.10 (t, *J* = 6.8 2H), 2.77 (t, *J* = 6.8 Hz, 2H), 1.16 (t, *J* = 7.0 Hz, 6H);^{13}C NMR ($CDCl_3$, 50 MHz) δ: 169.0, 167.9, 133.1, 130.1, 128.6, 127.8, 70.0, 62.0, 59.1, 37.0, 13.7; MS (ESI): *m*/*z* = 290 $[M + H]^+$; HRMS calculated for $[C_{16}H_{19}NO_4 + H]^+$: 290.1392, found 290.1396.

8.6. Diethyl 5-(3-chlorophenyl)-3,4-dihydro-2*H*-pyrrole-4,4-dicarboxylate (21f)

Mol. Wt: $C_{16}H_{18}ClNO_4$, Mol. Wt: 323.77

IR (neat, cm^{-1}): 3325, 3066, 2981, 1730, 1645, 1541, 1473, 1369, 1265, 1178, 1024, 858, 806, 752, 682; ^{1}H NMR ($CDCl_3$, 400 MHz) δ: 7.9 (t, 1H , *J* = 1.8), 7.75 (dt, *J* = 1.6, *J* = 7.6, 1H), 7.38–7.36 (m, 1H), 7.28 (m, 1H), 4.22 (m, 4H), 4.1 (t, *J* = 7.2 Hz, 2H), 2.77 (t, *J* = 7.0 Hz, 2H), 1.20 (t, *J* = 7.2 Hz, 6H); ^{13}C NMR ($CDCl_3$, 50 MHz) δ: 168.7, 166.8, 146.3, 134.9, 133.9, 130.1, 129.0, 128.8, 126.8, 166.1, 70.1, 62.2, 59.1, 36.9, 30.1, 21.4, 13.8; MS (ESI): *m*/*z* = 324/326 $[M + H]^+$; HRMS calculated for $[C_{16}H_{18}ClNO_4 + H]^+$: 324.1003, found 324.0992.

8.7. Diethyl 3,4-dihydro-5-(4-fluorophenyl)-2*H*-pyrrole-4,4-dicarboxylate (21g)

M.F.: $C_{16}H_{18}FNO_4$, Mol. Wt: 307.32

IR (neat, cm^{-1}): 3450, 3073, 2983, 2869, 1732, 1602, 1590, 1510, 1446, 1390, 1367, 1261, 1179, 1085, 1014, 846, 813, 758, 590; ^{1}H NMR ($CDCl_3$, 400 MHz) δ: 7.90 (m, 2H), 7.07–7.01 (m, Hz, 2H), 4.24–4.17 (m, 4H), 4.09 (t, *J* = 6.8 Hz, 2H), 2.77 (t, *J* = 6.8 Hz, 2H), 1.18 (t, *J* = 7.2 Hz, 6H); ^{13}C NMR ($CDCl_3$, 100 MHz) δ: 169.3, 167.1 & 166.3, 162.1, 130.3 & 130.2, 129.2 &129.1, 115.6 &115.2, 61.7, 50.0, 38.0, 28.0,13.9; MS (ESI): *m*/*z* = 308 $[M + H]^+$; HRMS calculated for $[C_{16}H_{18}FNO_4 + H]^+$: 308.1298, found 308.1305.

8.8. Diethyl 3,4-dihydro-5-(4-nitrophenyl)-2*H*-pyrrole-4,4-dicarboxylate (21h)

M.F.: $C_{16}H_{18}N_2O_6$, Mol. Wt: 334.32

IR (neat, cm^{-1}): 3437, 3075, 2983, 2866, 1753, 1597, 1517, 1342, 1318, 1262, 1176, 1081, 1024, 854, 742, 690; ^1H NMR ($CDCl_3$, 400 MHz) δ: 8.22 (d, *J* = 8.8 Hz, 2H), 8.07 (d, *J* = 8.8 Hz, 2H), 4.25–4.21 (m, 6H), 2.80 (t, *J* = 6.8 Hz, 2H), 1.20 (t, *J* = 7.0 Hz, 6H); ^{13}C NMR ($CDCl_3$, 50 MHz) δ: 168.6, 166.3, 148.62, 139.1, 129.7, 123.0, 70.2, 62.4, 59.6, 36.8, 13.9; MS (ESI): *m*/*z* = 335 $[M + H]^+$; HRMS calculated for $[C_{16}H_{18}N_2O_6 + H]^+$ 335.1243, found 335.1251.

8.9. Diethyl 3,4-dihydro-5-(3-methoxyphenyl)-2*H*-pyrrole-4,4-dicarboxylate (21i)

M.F.: $C_{17}H_{21}NO_5$, Mol. Wt: 319.35

IR (neat, cm^{-1}): 3448, 3076, 2981, 2939, 2837, 1729, 1600, 1579, 1488, 1464, 1366, 1320, 1262, 1178, 1085, 1020, 863, 789, 693; ^1H NMR ($CDCl_3$, 400 MHz) δ: 7.48 (s, 1H), 7.39 (d, *J* = 8.0Hz, 1H), 7.25 (d, *J* = 8.4 Hz, 1H), 6.96 (dd,*J* = 2.2 Hz, *J* = 8.0 Hz, 1H), 4.29–4.23 (m, 4H), 4.10 (t, *J* = 6.8 Hz, 2H), 3.82 (s, 3H), 2.77 (t, *J* = 6.4 Hz, 2H), 1.18 (t, *J* = 7.2 Hz, 6H); ^{13}C NMR ($CDCl_3$, 100 MHz) δ: 169.3, 167.2, 159.6, 135.6, 129.4, 118.5, 117.7, 112.0, 61.6, 55.3, 50.0, 38.0, 28.1, 13.9; MS (ESI): *m*/*z* = 320 $[M + H]^+$; HRMS calculated for $[C_{17}H_{21}NO_5 + H]^+$ 320.1498, found 320.1491.

8.10. Diethyl 3,4-dihydro-5-(thiophen-2-yl)-2*H*-pyrrole-4,4-dicarboxylate (21j)

M.F.: $C_{14}H_{17}NO_4S$, Mol. Wt: 295.35

IR (neat, cm^{-1}): 3453, 3105, 2982, 1731, 1601, 1429, 1316, 1262, 1180, 1085, 1005, 848, 754; ^1H NMR (400 MHz, $CDCl_3$) δ: 7.45 (dd, , *J* = 1.0 Hz, *J* = 4.2 Hz, 1H), 7.38 (dd, *J* = 1.0 Hz, *J* = 3.8 Hz 1H), 7.01 (dd, *J* = 3.6 Hz, 5.2 Hz, 1H), 4.28–4.16 (m, 4H), 4.19 (t, *J* = 3.4 Hz, 2H), 2.77 (t, *J* = 6.4 Hz, 2H), 1.21 (t, *J* = 7.0 Hz, 6H); ^{13}C NMR (50 MHz, $CDCl_3$) δ: 168.6, 162.2, 137.4, 130.2, 129.1, 127.2, 70.4, 62.1, 59.2, 36.5, 13.8; MS (ESI): *m*/*z* = 296 $[M + H]^+$; HRMS calculated for $[C_{14}H_{17}NO_4S + H]^+$ 296.0957, found 296.0963.

9. Spectral data of 23, 24, 28, 29, 31 and 32

9.1. 5-phenyl-3,4-dihydro-2*H*-pyrrole (23)

M.F.: $C_{10}H_{11}N$, Mol. Wt: 145.09

IR (neat, cm^{-1}): 3390, 3057, 2960, 2860, 1616, 1573, 1494, 1446, 1340, 1311, 1178, 1076, 1047, 1026, 988, 966, 921; ^{1}H NMR ($CDCl_3$, 400 MHz) δ: 7.85–7.82 (m, 2H), 7.42–7.36 (m, 3H), 4.07–4.03 (m, 2H), 2.96–2.91 (m, 2H), 2.06–1.98 (m, 2H); ^{13}C NMR ($CDCl_3$, 100 MHz) δ: 173.1, 134.5, 130.1, 128.2, 127.4, 61.3, 34.8, 22.5; MS (ESI): *m*/*z* = 146.1 $[M + H]^+$; HRMS calculated for $[C_{10}H_{11}N + H]^+$:146.0970, found 146.0972.

9.2. Ethyl 5-phenyl-3,4-dihydro-2*H*-pyrrole-4-carboxylate (24)

M.F.: $C_{13}H_{15}NO_2$, Mol. Wt: 217.26

IR ($CHCl_3$, cm^{-1}): 2980, 1730,1617, 1446, 1368, 1327, 1254, 1219, 1157, 1044; ^{1}H NMR ($CDCl_3$, 400 MHz) δ: 7.87–7.85 (dd, *J* =1.8 Hz, *J* = 2H), 7.42–7.38 (m, 3H), 4.20–4.06 (m, 5H), 2.38–2.32 (m, 2H), 1.14 (t, *J* = 7.0); ^{13}C NMR ($CDCl_3$, 100 MHz) δ: 171.9, 169.1, 133.1, 130.3, 128.2, 127.6, 60.85, 60.81, 53.3, 29.47, 29.42, 29.1,29.05, 13.8, 13.7; MS (ESI): *m*/*z* = 218.2 $[M + H]^+$; HRMS calculated for $[C_{13}H_{15}NO_2 + H]^+$:218.1181, found 218.1180.

9.3. Ethyl 3-(aziridin-1-yl)-2-cyano-3-phenylacrylate (28)

M.F.: $C_{14}H_{14}N_2O_2$, Mol. Wt: 242.27

IR (neat, cm^{-1}): 3019, 2401, 2215, 1712, 1581, 1538, 1488, 1473, 1403, 1283, 1249, 1216, 1174, 1135, 1108, 1062, 1038, 1018, 850; ^{1}H NMR ($CDCl_3$, 400 MHz) δ: 7.56–7.2 (m, 5H), 4.29 and 4.09 (q, *J* = 7.0 Hz and q, J = 7.2 Hz, 2H), 2.48 and 2.44 (s, s, 4H), 1.37 and 1.17 (t, *J* = 7.0 Hz and t, *J* = 7.2 Hz, 3H); ^{13}C NMR ($CDCl_3$, 100 MHz) δ: 176.8 and 176.0, 162.68, 136.1 and 135.8, 130.67 and 130.0, 128.6 and 128.1, 127.4 and 127.1, 117.88 and 117.24, 87.8, 87.6, 61.0 and 60.9, 32.7 and 30.0, 14.2 and 13.9; MS (ESI): *m*/*z* = 243.1 $[M + H]^+$.

9.4. Ethyl 4-cyano-5-phenyl-3,4-dihydro-2*H*-pyrrole-4-carboxylate (29)

M.F.: $C_{14}H_{14}N_2O_2$, Mol. Wt: 242.27

IR (neat, cm^{-1}): 2983, 2937, 2869, 2244, 2210, 1742, 1667, 1626, 1577, 1496, 1368, 1320, 1252, 1195, 1097, 1073, 1008, 854, 779, 693; ^{1}H NMR ($CDCl_3$, 400 MHz) δ: 7.93 (d, *J* = 7.6 Hz, 2H), 7.52–7.42 (m, 3H), 4.35–4.22 (m, 4H), 2.82–2.78 (m, 2H), 1.25–1.21

(m, 3H) ; ^{13}C NMR ($CDCl_3$, 100 MHz) δ: 166.6, 163.4, 131.3, 129.0, 128.6, 127.8, 116.9, 63.4, 60.4, 56.3, 37.9, 13.6; MS (ESI): *m*/*z* = 243.2 $[M + H]^+$; HRMS calculated for $[C_{14}H_{14}N_2O_2 + H]^+$: 243.1134, found 243.1133.

9.5. 2-Butylaziridine (30)

IR (neat, cm^{-1}): 2922, 2851, 1595, 1464, 1219, 772 ; ^{1}H NMR ($CDCl_3$, 400 MHz) δ: 1.94–1.91 (m, 1H), 1.75 (d, *J* = 5.6 Hz), 1.48–1.32 (m, 7H), 0.91 (t, *J* = 7.0 Hz, 3H) ^{13}C NMR ($CDCl_3$, 100 MHz) δ: 34.0, 30.3, 29.6, 25.0, 22.4, 13.9; MS (ESI): *m*/*z* = 100.1 $[M + H]^+$, 199.1 $[2M + H]^+$.

9.6. Diethyl 2-[(2-butylaziridin-1-yl)phenylmethylene]malonate (31)

M.F.: $C_{20}H_{27}NO_4$, Mol. Wt: 345.43

IR (neat, cm^{-1}): 3020, 2961, 2933, 2400, 1709, 1605, 1584, 1570, 1491, 1445, 1412, 1369, 1337, 1276, 1215, 1155, 1080, 1026, 928, 851; ^{1}H NMR ($CDCl_3$, 400 MHz) δ: 7.36–7.29 (m, 5H), 4.33–4.24 (m, 2H), 3.88–3.94 (m, 2H), 2.28–2.27 (d, *J* = 3.2 Hz, 1H), 2.20–2.15 (m, 2H), 1.62–1.57 (m, 2H), 1.33 (t, *J* = 7.2 Hz, 3H), 1.19–1.08 (m, 4H), 0.93 (t, *J* = 7.0 Hz, 3H), 0.79 (t, *J* = 3.2 Hz, 3H) ; ^{13}C NMR ($CDCl_3$, 100 MHz) δ: 166.0,166.9, 164.5, 137.5, 128.7, 127.9, 127.5, 110.2, 60.6 and 60.5, 41.6, 37.8, 32.0, 28.3, 22.2, 14.2, 13.8, 13.6; MS (ESI): *m*/*z* = 346.2 $[M + H]^+$.

9.7. Diethyl 2-butyl-3,4-dihydro-5-phenyl-2*H*-pyrrole-4,4-dicarboxylate (32)

M.F.: $C_{20}H_{27}NO_4$, Mol. Wt: 345.43

IR ($CHCl_3$, cm^{-1}): 2932, 1730, 1606.3, 1446, 1367, 1258, 1219, 1184, 1126, 1096, 1061; ^{1}H NMR ($CDCl_3$, 400 MHz) δ: 7.87–7.84 (m, 2H), 7.39–7.31(m, 3H), 4.24–4.13 (m, 5H), 2.96-2.91 (dd, *J* = 6.8 Hz, *J* = 13.6 Hz, 1H); 2.32–2.26 (dd, *J* = 7.8 Hz, *J* = 13.0 Hz, 1H), 1.89–1.84 (m, 1H), 1.56–1.37 (m, 5H), 1.19 (t, *J* = 7.2 Hz, 3H), 1.13 (t, *J* = 7.2 Hz, 3H), 0.94 (t, *J*= 7.2 Hz, 3H); ^{13}C NMR ($CDCl_3$, 100 MHz) δ: 169.7, 168.8, 166.3, 133.3, 130.0, 128.7, 127.8, 70.9, 70.6, 62.0, 61.9, 42.4, 35.6, 28.9, 22.7, 14.0, 13.8, 13.7; MS (ESI): *m*/*z* = 346.2 $[M + H]^+$; HRMS calculated for $[C_{20}H_{27}NO_4 + H]^+$: 346.2018, found 346.2006.

10. References

1. Rathke, M. W.; Cowan P. J. *J. Org. Chem*. **1985**, *50*, 2622. doi:10.1021/jo00215a003
2. Jung, J. C.; Watkins, E. B.; Avery, M. A. *Synth.Commun.* **2002**, *24*, 3767 – 3778 doi: 10.1081/SCC-120015395
3. Bankowska, Z.; Zadrozna, I.; Bochenska, J. *Polish Journal of Chemistry*; **1980**, *54*, 461 – 467.
4. Brown, E. J. D.; Harley-Mason, J. *J. Chem. Soc. [Section] C: Organic* **1966**, 1390-1394.
5. Hormi, O. *Org. Synth.* **1993**, *coll. vol. 8*, 247-250.
6. Viehe, H. G. *Angewandte Chemie.* 1971, *83*, 616 – 617. doi: 10.1002/ange.19710831606
7. Hormi, Osmo E. O. *Synth.Commun.* **1986**, *16*, 997-1002
8. Rappoport, Z.; Topol, A. *J. Chem. Soc.*, *Perkin Transactions 2: Physical Organic Chemistry* (1972-1999); **1975**, 863 – 874.
9. Goorge, A. D.; Mary, D.; Mary C. C. Pyrazolo-‘3,4-B! Compounds And Their Use As Phosphodiesterase Type 4 (PDE4) Inhibitors. WO Patent 2005/090348, September 29, 2005.
10. Stavinoha, J. L.; Mariano, P. S. *J. Am. Chem. Soc.* **1981,** *103,* 3136-3148.
11. Kumar, K. A.*;* Rai, *L.,* K. M.; Umesha, K. B. *Tetrahedron* **2001**, *57*, 6993-6996, doi:10.1016/S0040-4020(01)00612-3
12. Tsuge, O.; Kanemasa, S.; Matsuda, K. *J. Org. Chem.* **1986***, 51*, 1997-2004
13. Jalander, L. F. *Synth. Commun*. **1993**, *23*, 2293-2302.
14. Kolb, H. C.; Kanamarlapudi, R. C.; Richardson, P. F.; Khan, G. Large Scale Synthesis Of Optically Pure Aziridines. US Patent 0153771 A1, August 14, 2003.

12. Spectra of 2-[(aziridin-1-yl)-1-alkyl/aryl/heteroaryl-methylene]malonic acid diethyl esters (*N*-vinyl aziridines) - 20a, 20c, 20d, 20f–20h

^{1}H NMR spectrum of 20a

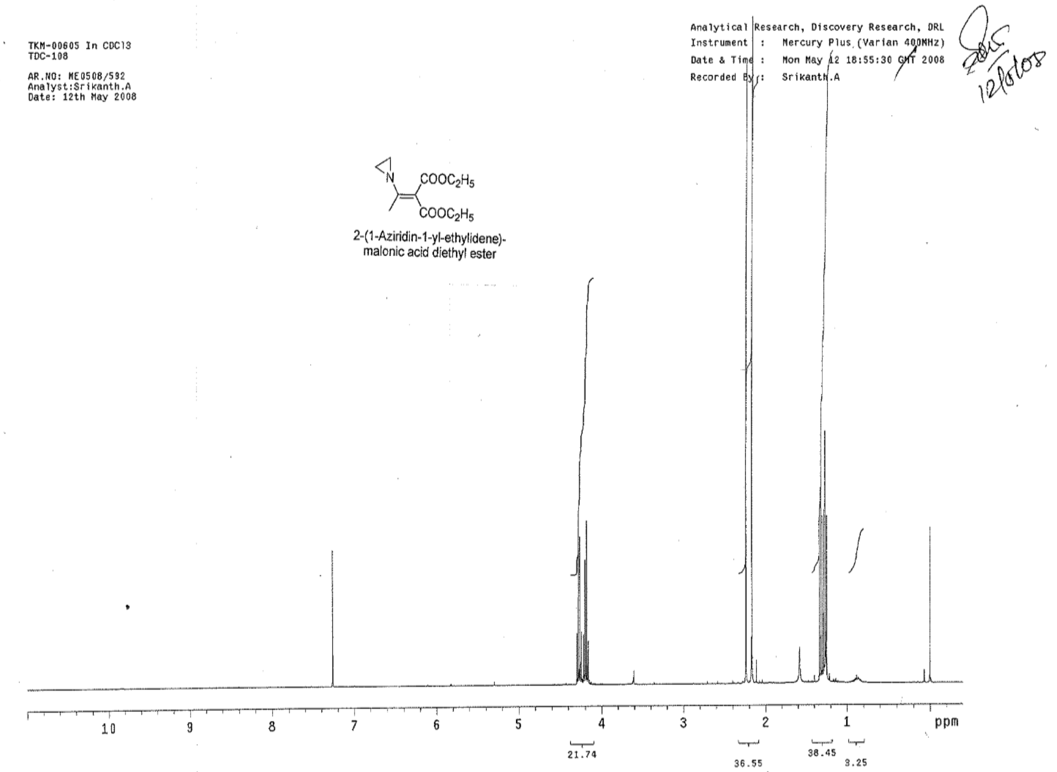

^{13}C NMR spectrum of 20a

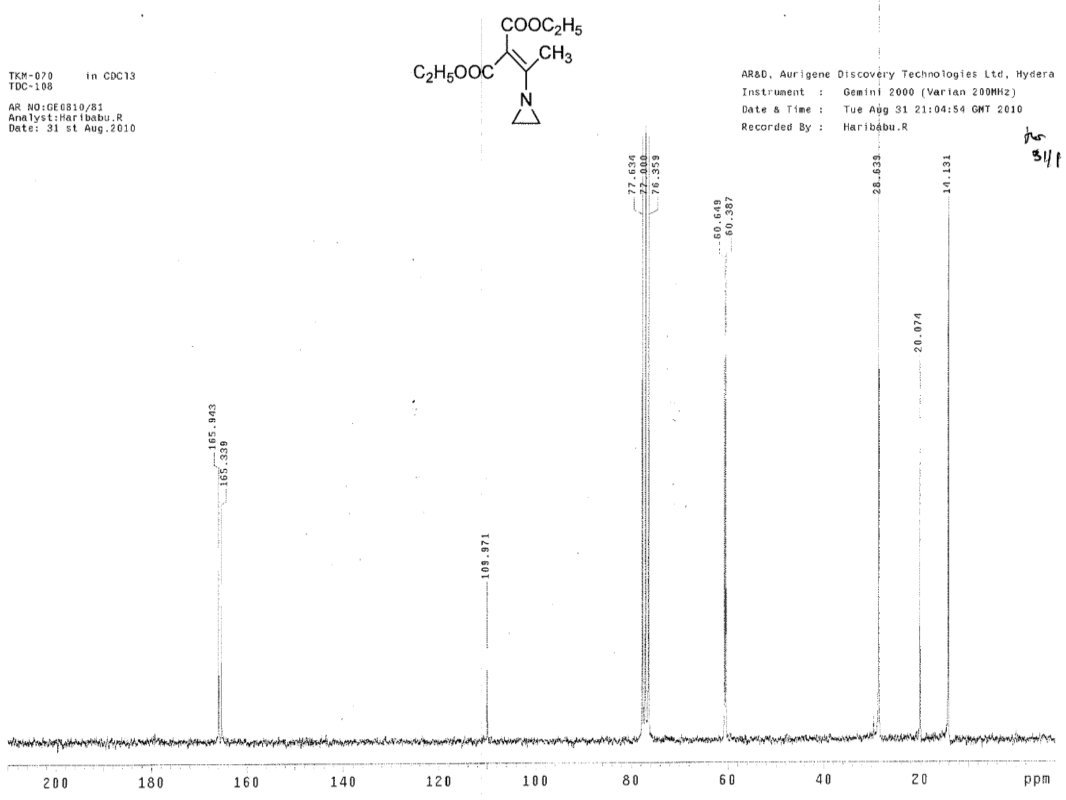

Mass spectrum of 20a

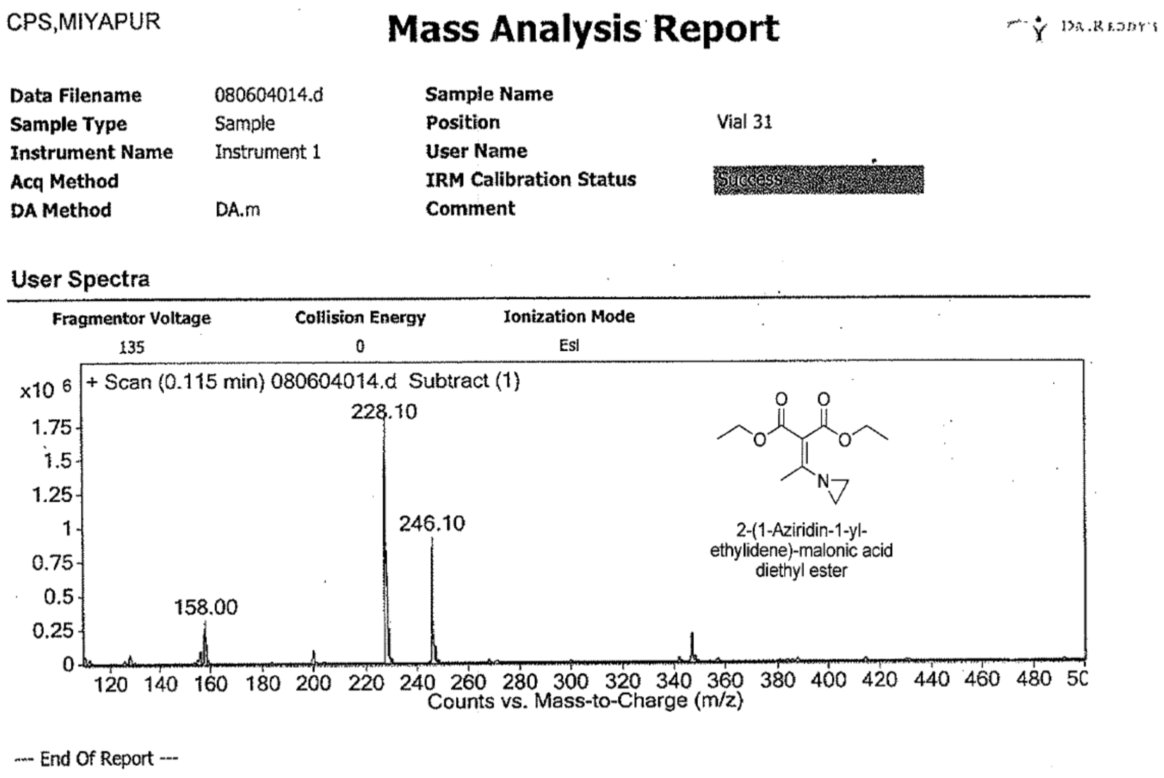

IR spectrum of 20a

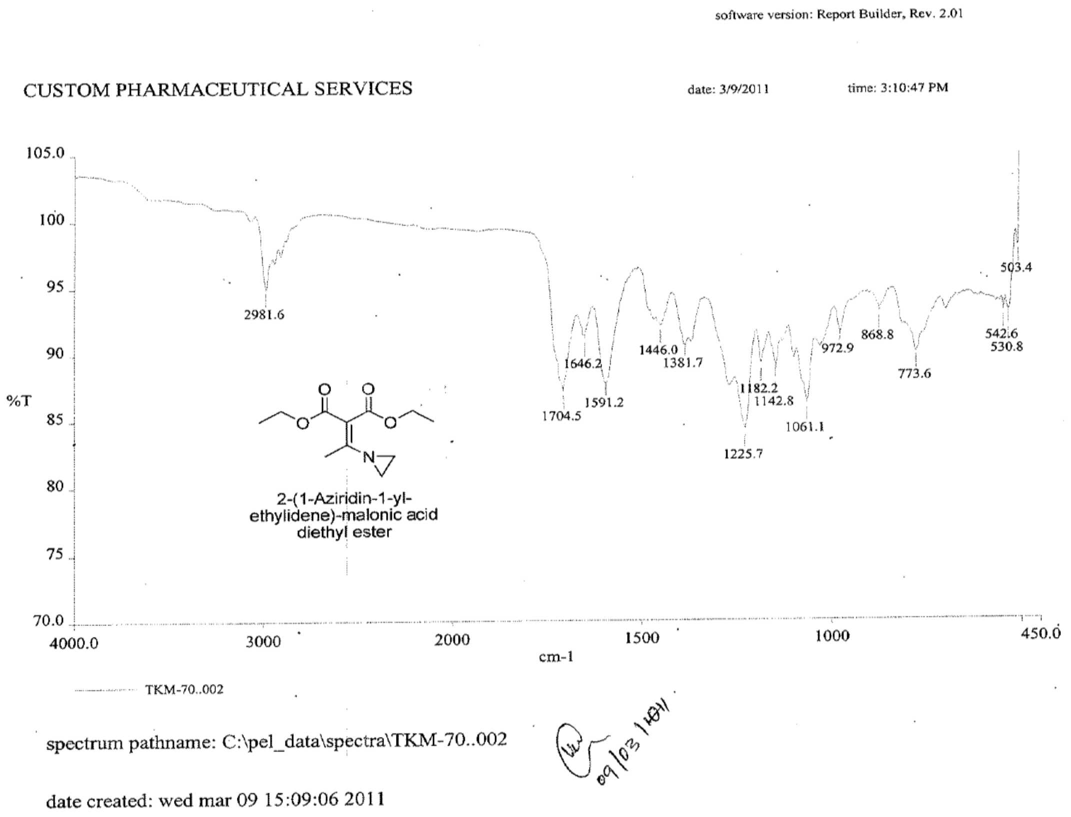

^{1}H NMR spectrum of 20c

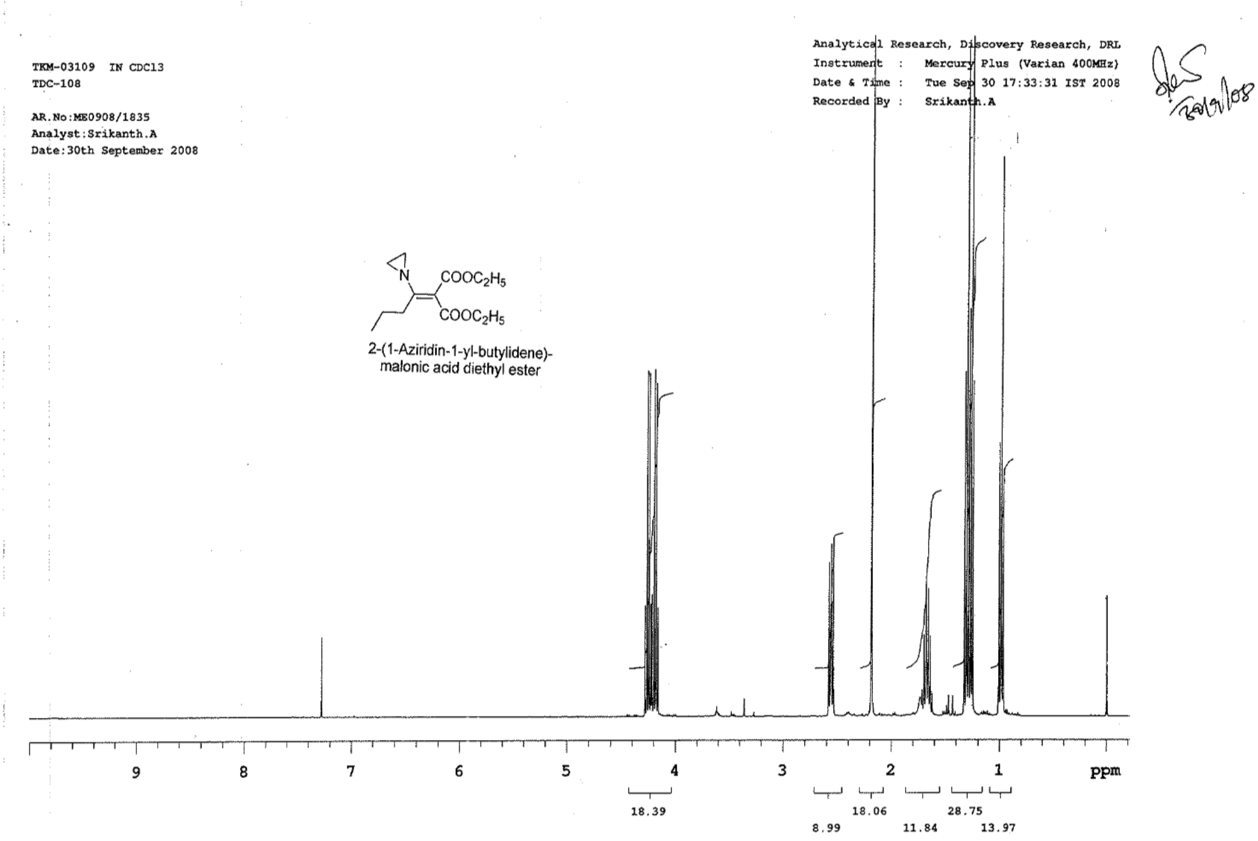

^{13}C NMR spectrum of 20c

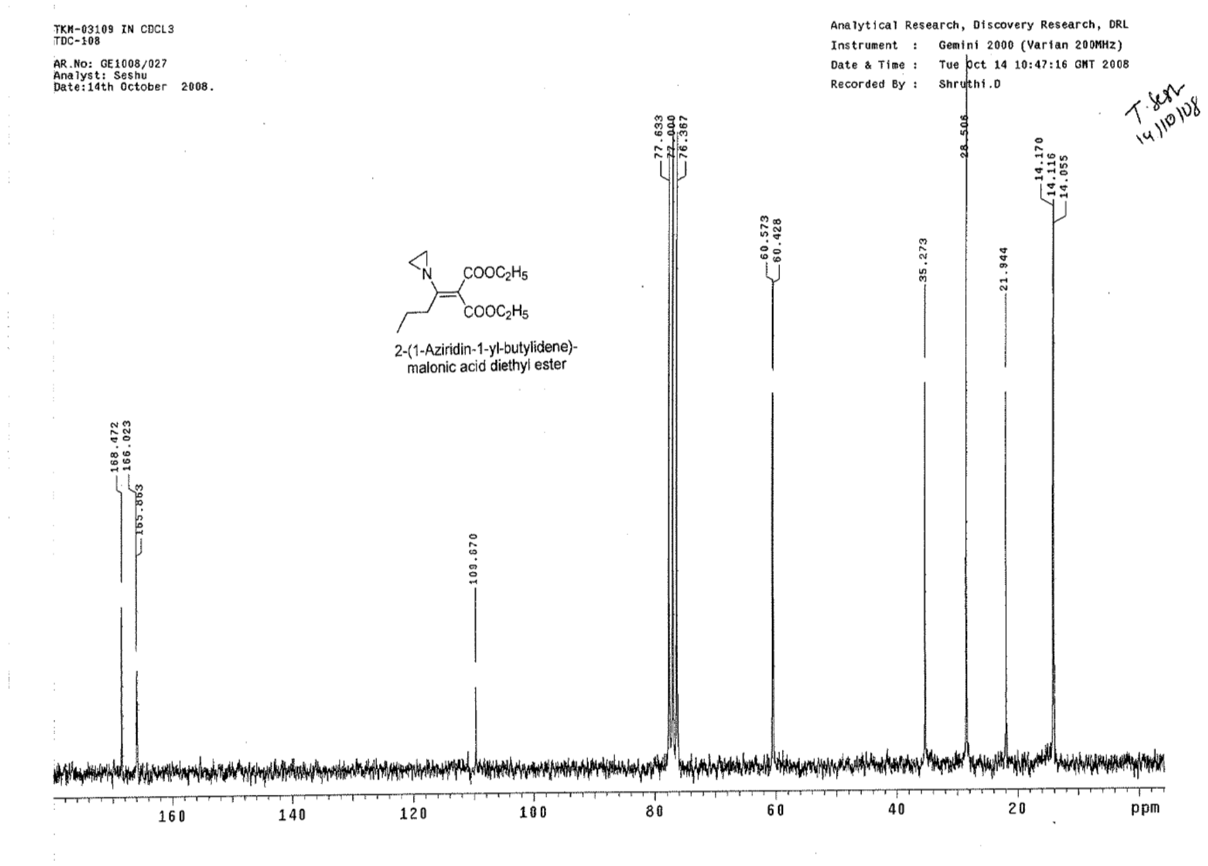

Mass spectrum of 20c

CPS,MIYAPUR

Mass Analysis Report

DR.REDDY'S

**Data Filename**	081001016.d	**Sample Name**	TKM-03109
**Sample Type**	Sample	**Position**	Vial 53
**Instrument Name**	Instrument 1	**User Name**	
**Acq Method**		**IRM Calibration Status**	Success
**DA Method**	DA.m	**Comment**	

User Spectra

Fragmentor Voltage	Collision Energy	Ionization Mode
100	0	Esi

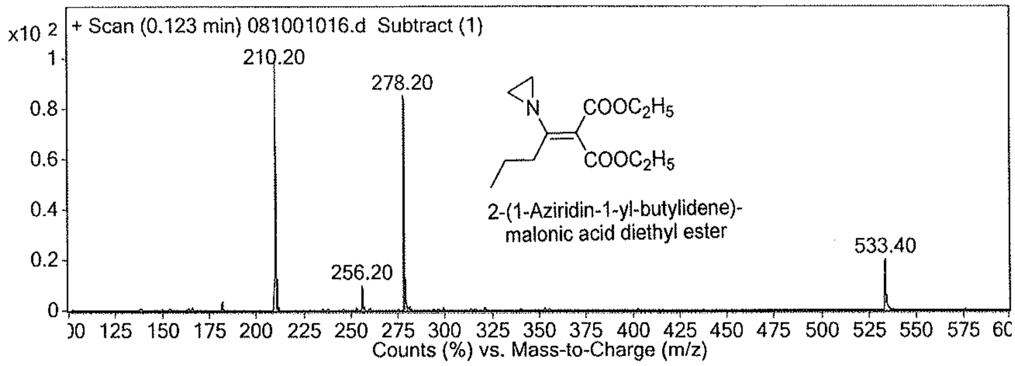

--- End Of Report ---

IR spectrum of 20c

DR.REDDY'S LABORATORIES LIMITED

TDC/CCS-ANALYTICAL RESEARCH

Date: 9/30/08

Time: 3:34:24 PM

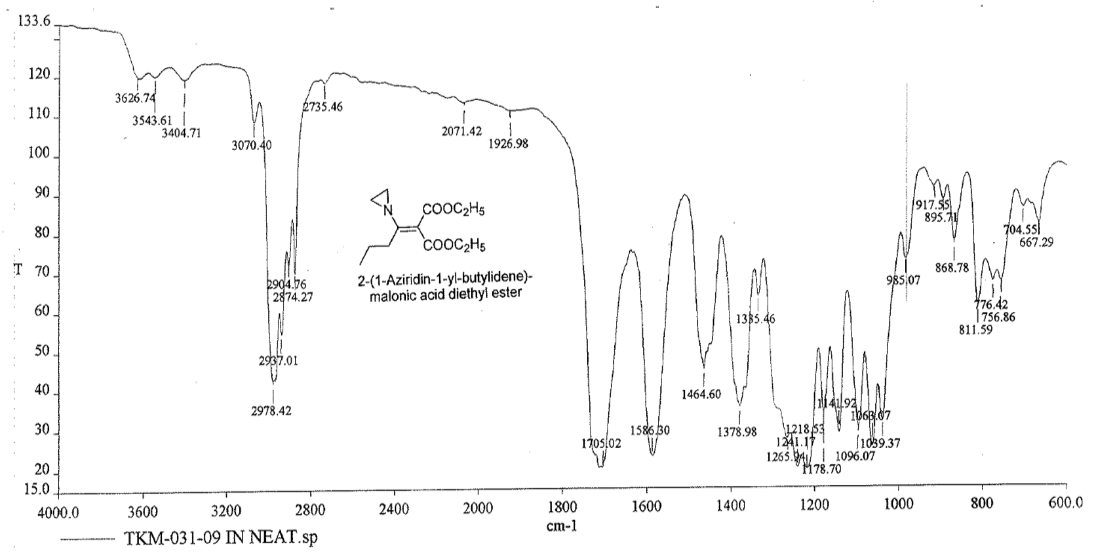

Analyst: APARNA

SIGN: 30/09/08

AR008130|SAM|116

^{1}H NMR spectrum of 20d

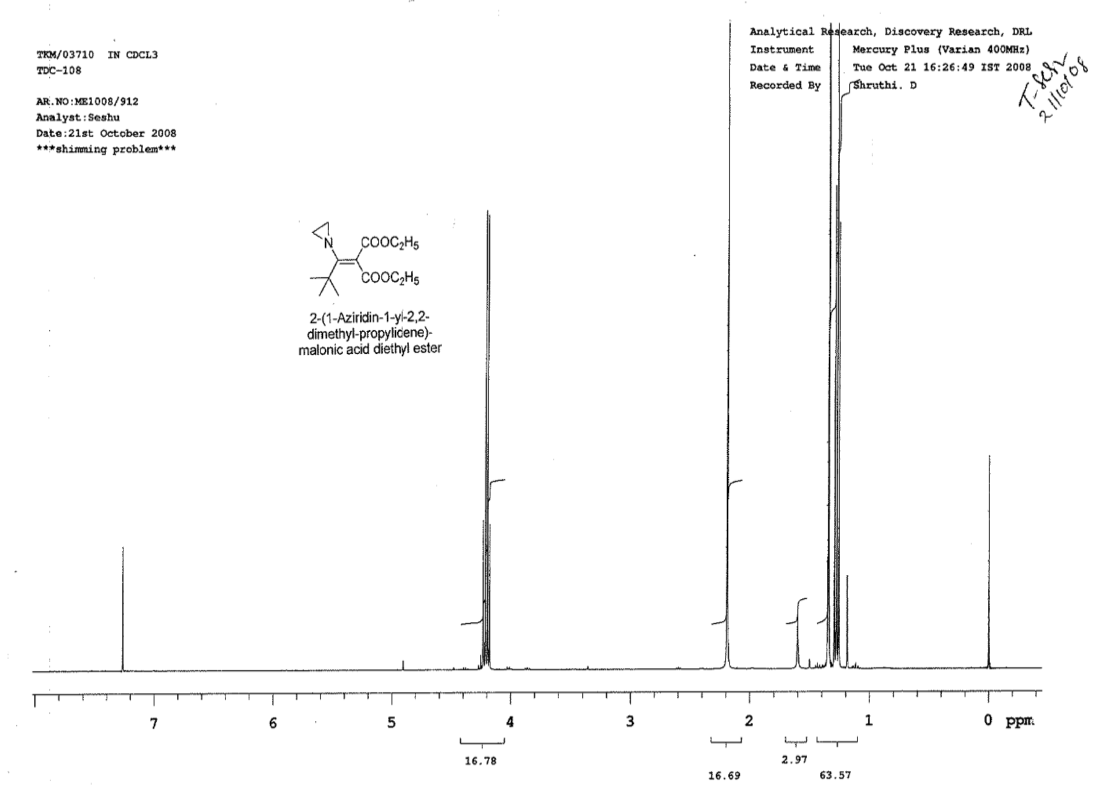

^{13}C NMR spectrum of 20d

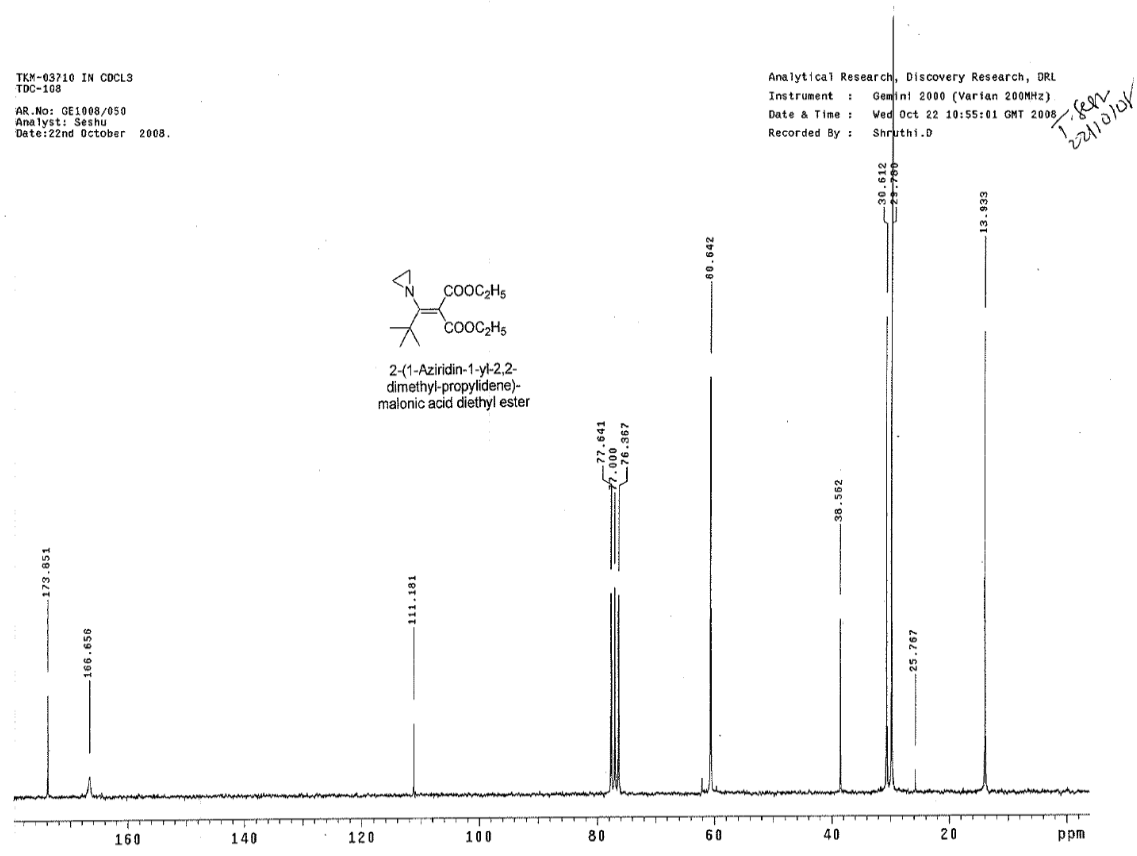

Mass spectrum of 20d

CPS,MIYAPUR

Mass Analysis Report

**Data Filename**	081022007.d	**Sample Name**	TKM-03710
**Sample Type**	Sample	**Position**	Vial 7
**Instrument Name**	Instrument 1	**User Name**	
**Acq Method**		**IRM Calibration Status**	Success
**DA Method**	DA.m	**Comment**	

User Spectra

Fragmentor Voltage	Collision Energy	Ionization Mode
135	0	Esl

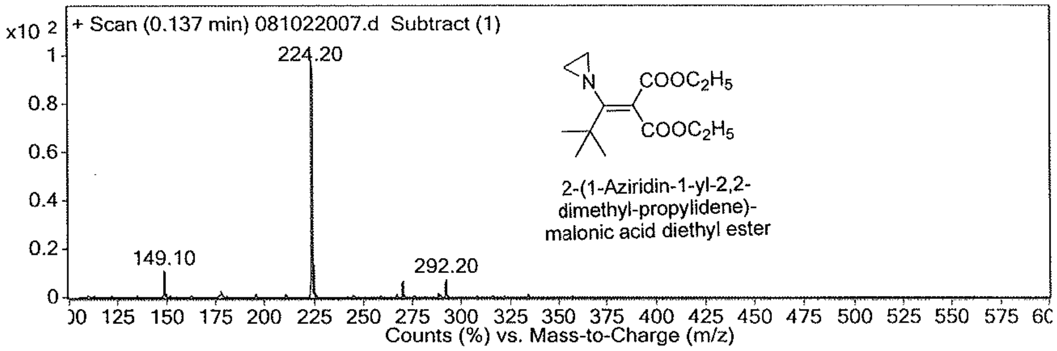

--- End Of Report ---

IR spectrum of 20d

DR.REDDY'S LABORATORIES LIMITED

TDC/CCS-ANALYTICAL RESEARCH .

Date: 10/21/08

Time: 3:08:40 PM

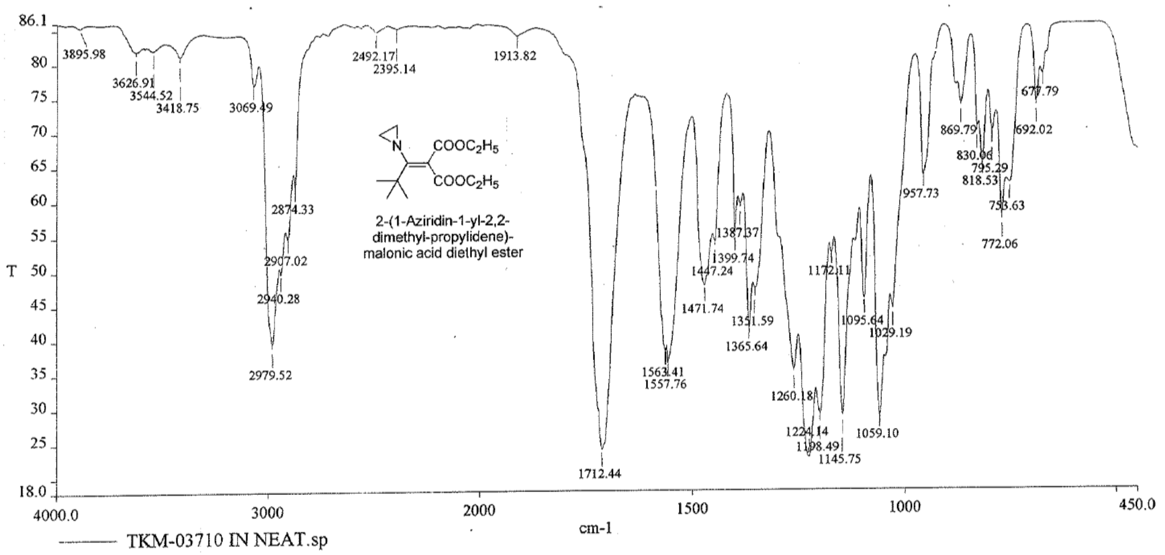

Analyst: SHYAM

SIGN: 21/10/08

^{1}H NMR spectrum of 20f

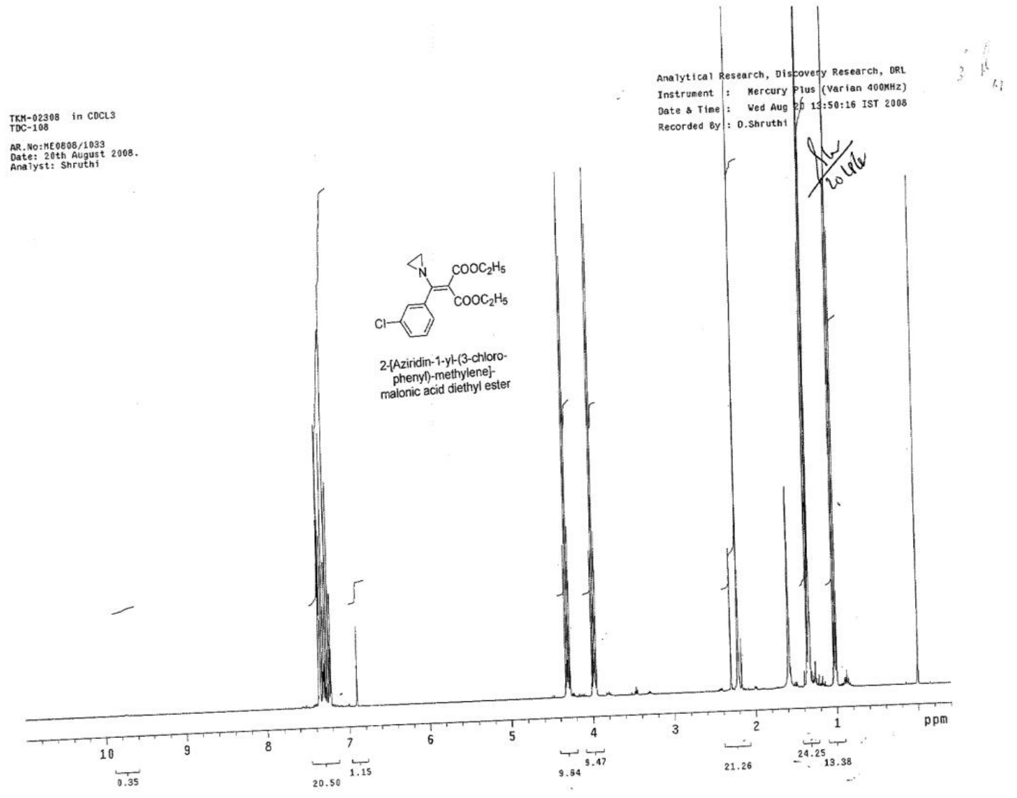

^{13}C NMR spectrum of 20f

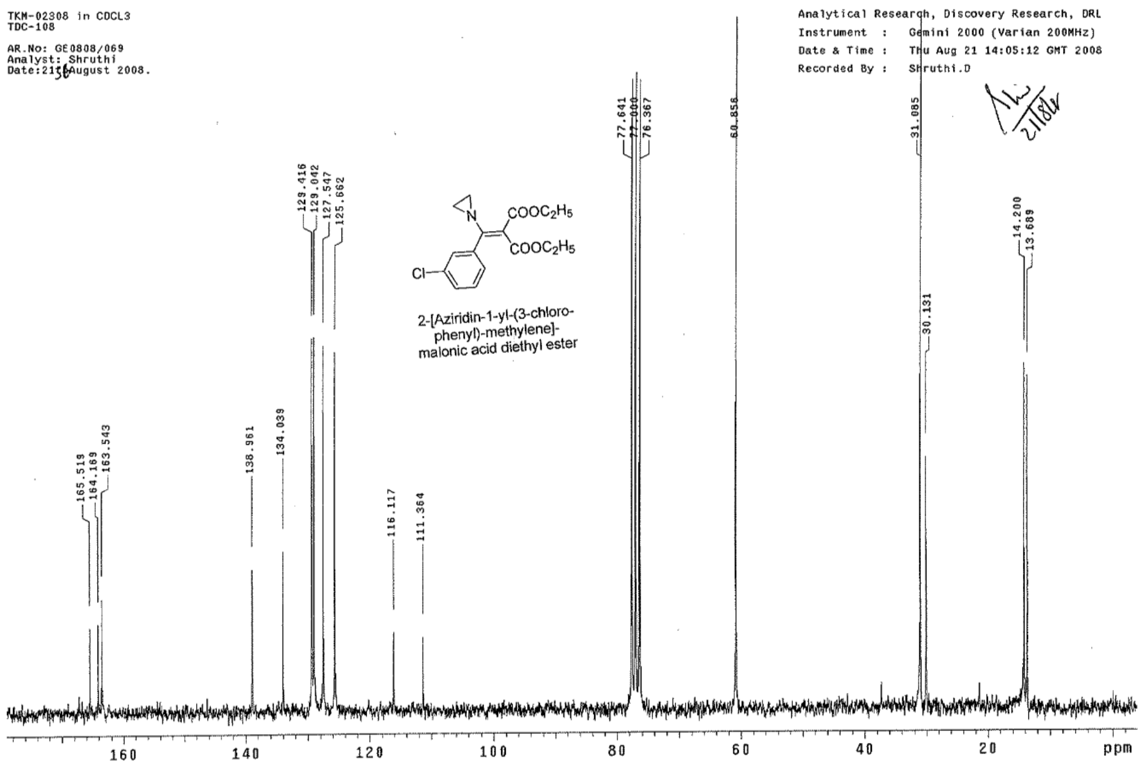

Mass spectrum of 20f

CPS,MIYAPUR

Mass Analysis Report

**Data Filename**	080821046.d	**Sample Name**	TKM-02308
**Sample Type**	Sample	**Position**	Vial 70
**Instrument Name**	Instrument 1	**User Name**	
**Acq Method**		**IRM Calibration Status**	Success
**DA Method**	DA.m	**Comment**	

User Spectra

Fragmentor Voltage	Collision Energy	Ionization Mode
135	0	Esi

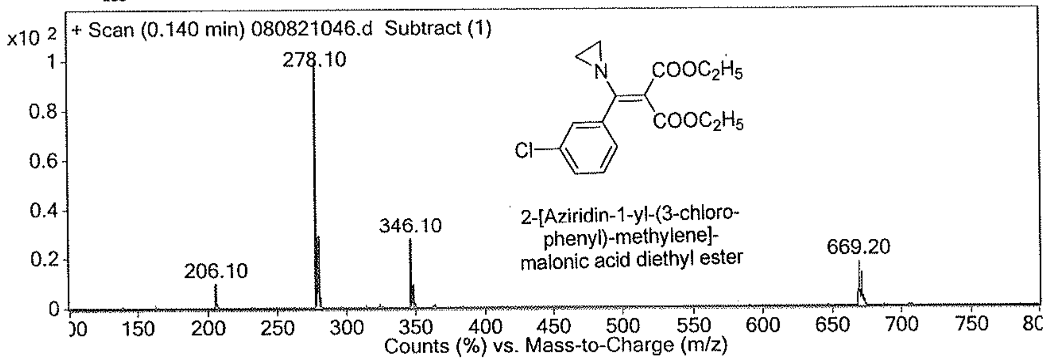

--- End Of Report ---

IR spectrum of 20f

DR.REDDY'S LABORATORIES LIMITED

TDC/CCS-ANALYTICAL RESEARCH .

Date: 8/20/08

Time: 10:25:12 AM

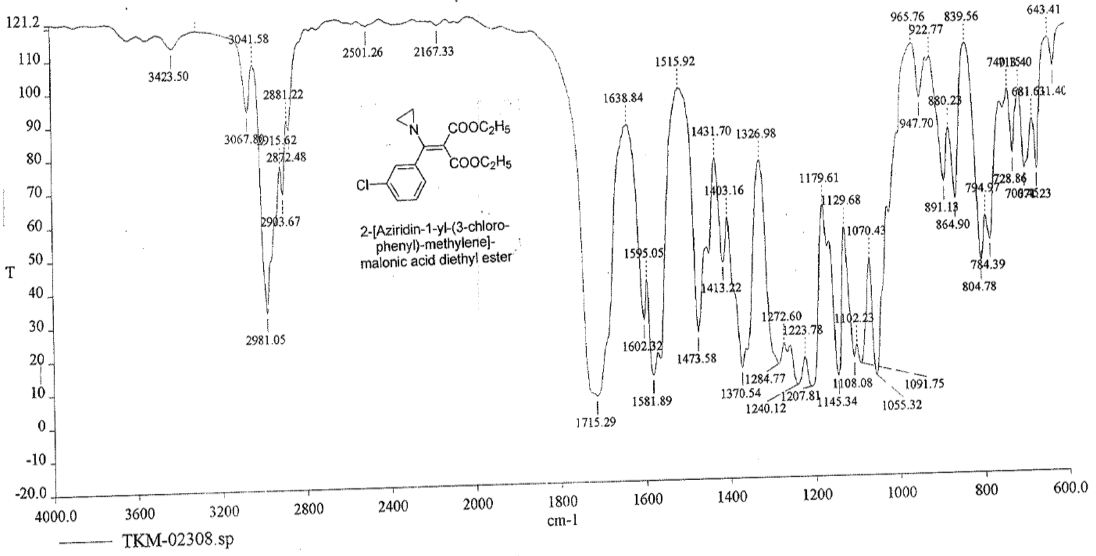

Analyst: SHYAM

SIGN:

20/08/08

^{1}H NMR spectrum of 20g

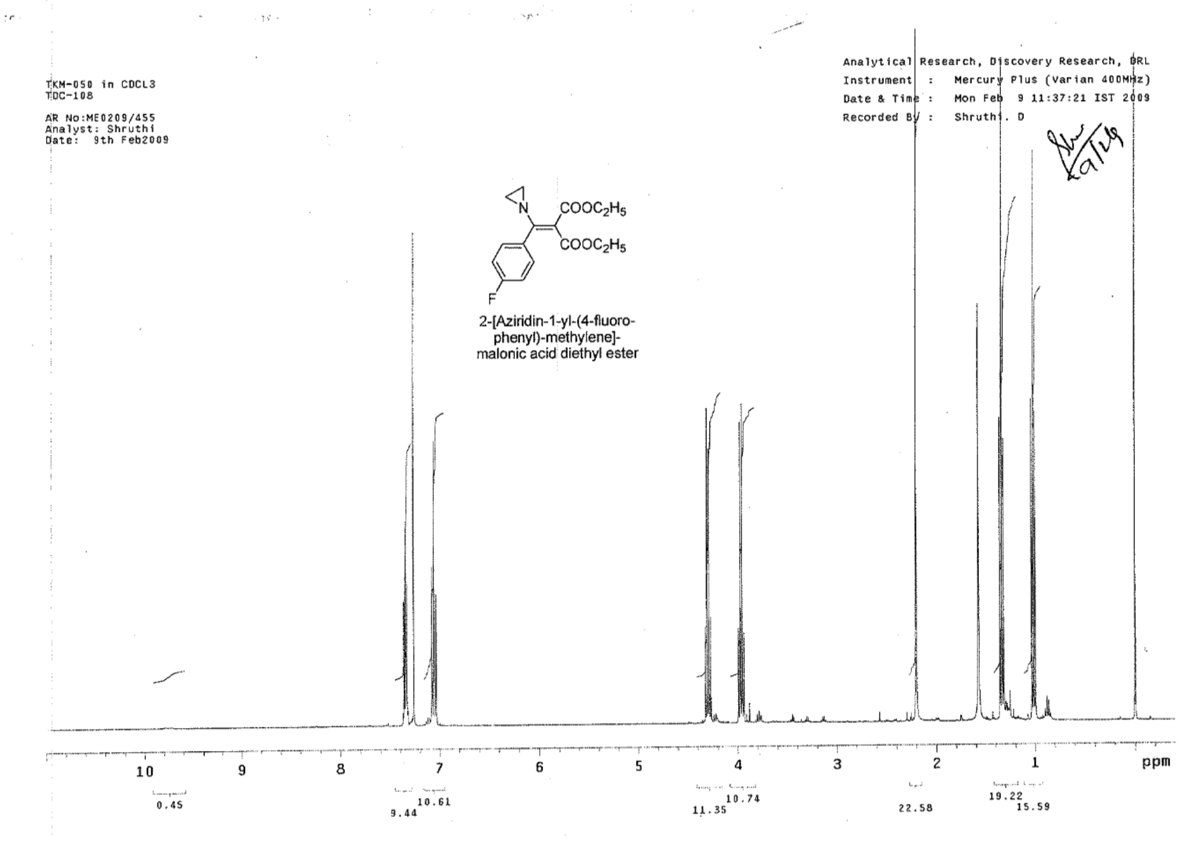

^{13}C NMR spectrum of 20g

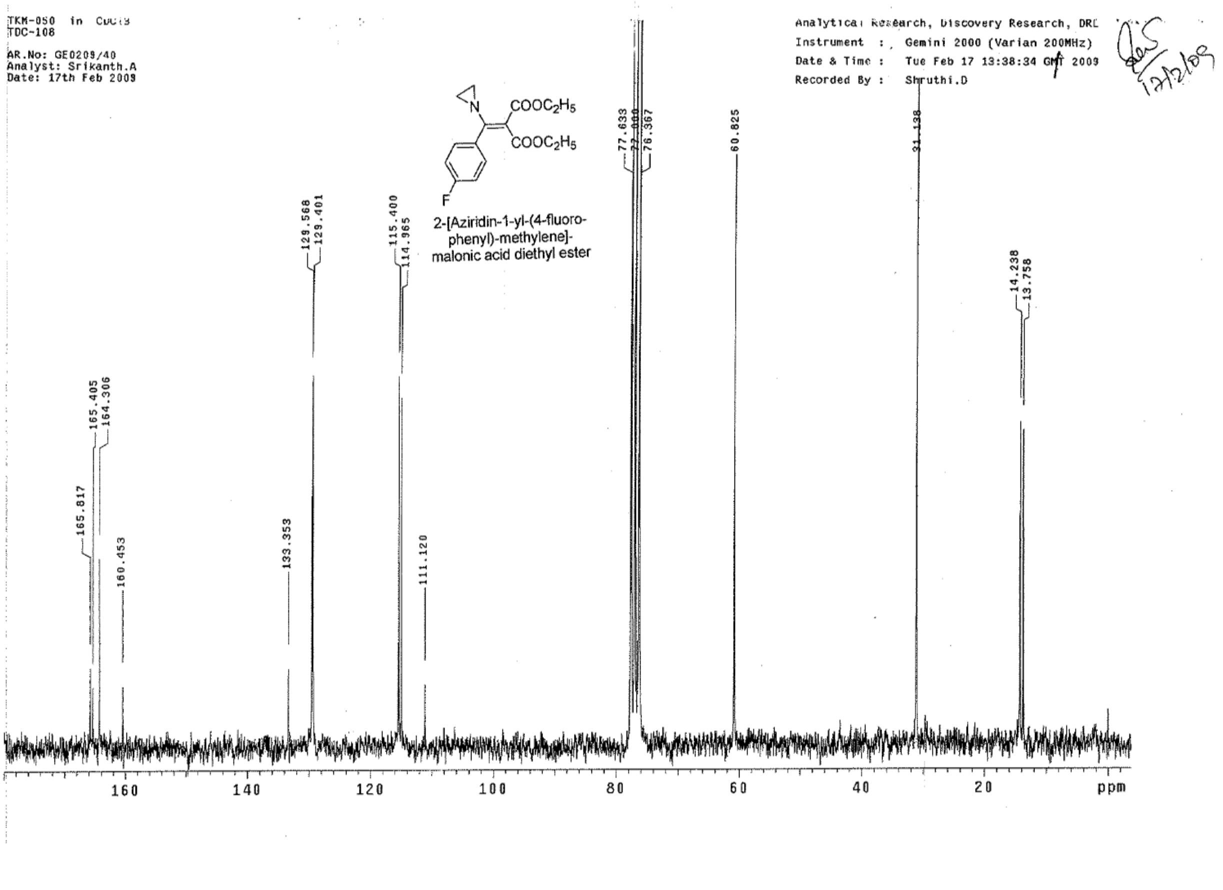

Mass spectrum of 20g

CPS,MIYAPUR

Mass Analysis Report

DR.REDDY'S

**Data Filename**	090209013.d	**Sample Name**	TKM-050
**Sample Type**	Sample	**Position**	Vial 11
**Instrument Name**	Instrument 1	**User Name**	
**Acq Method**	ESI.m	**IRM Calibration Status**	Success
**DA Method**	DA.m	**Comment**	

User Spectra

Fragmentor Voltage	Collision Energy	Ionization Mode
70	0	Esi

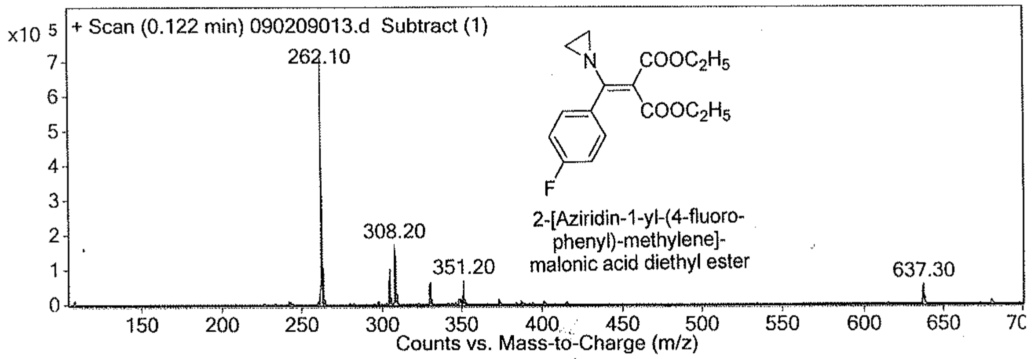

--- End Of Report ---

IR spectrum of 20g

DR.REDDY'S LABORATORIES LIMITED

TDC/CCS-ANALYTICAL RESEARCH

Date: 2/17/09

Time: 5:34:38 PM

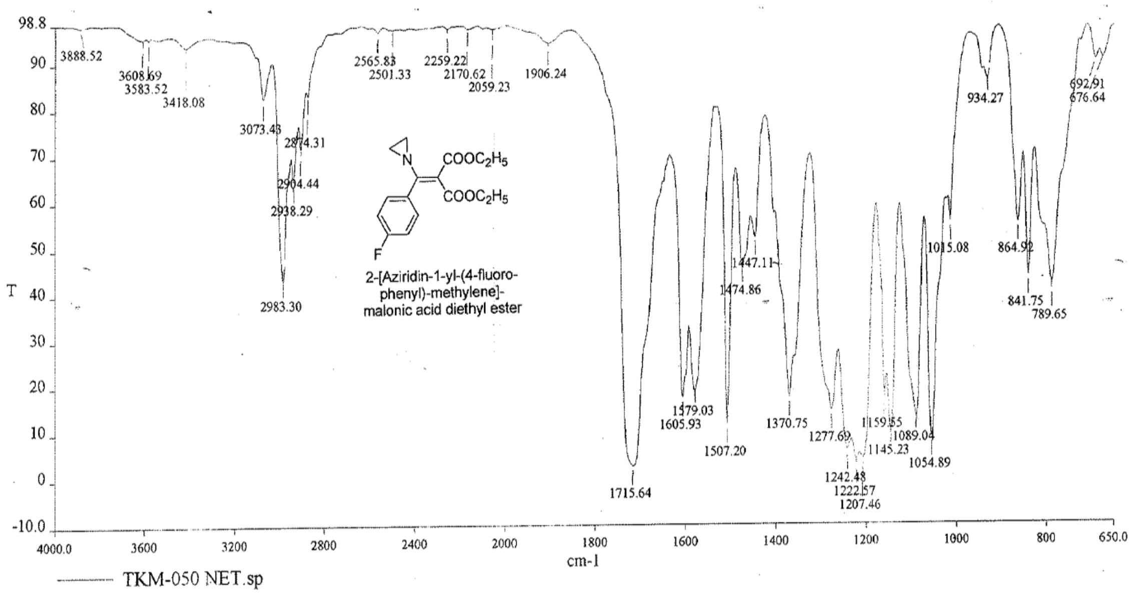

ARD09052/AP/084

Analyst: APARNA

SIGN:

12/02/09

^{1}H NMR spectrum of 20h

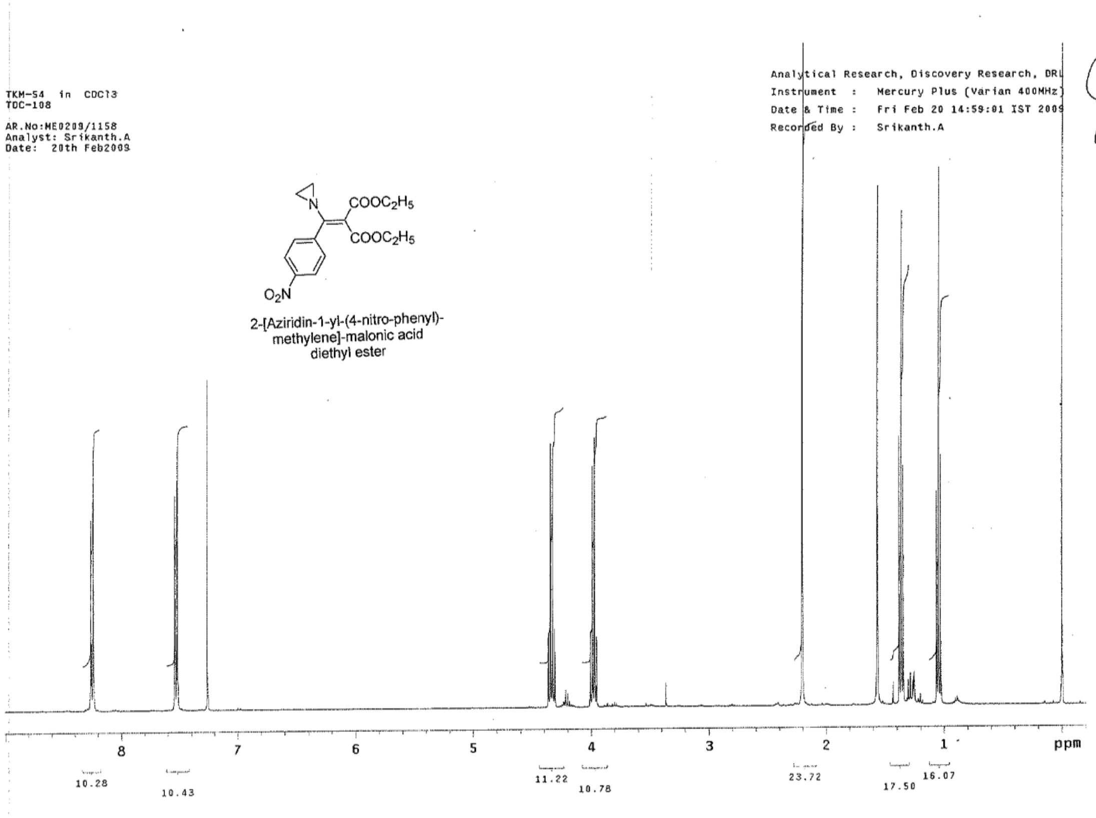

^{13}C NMR spectrum of 20h

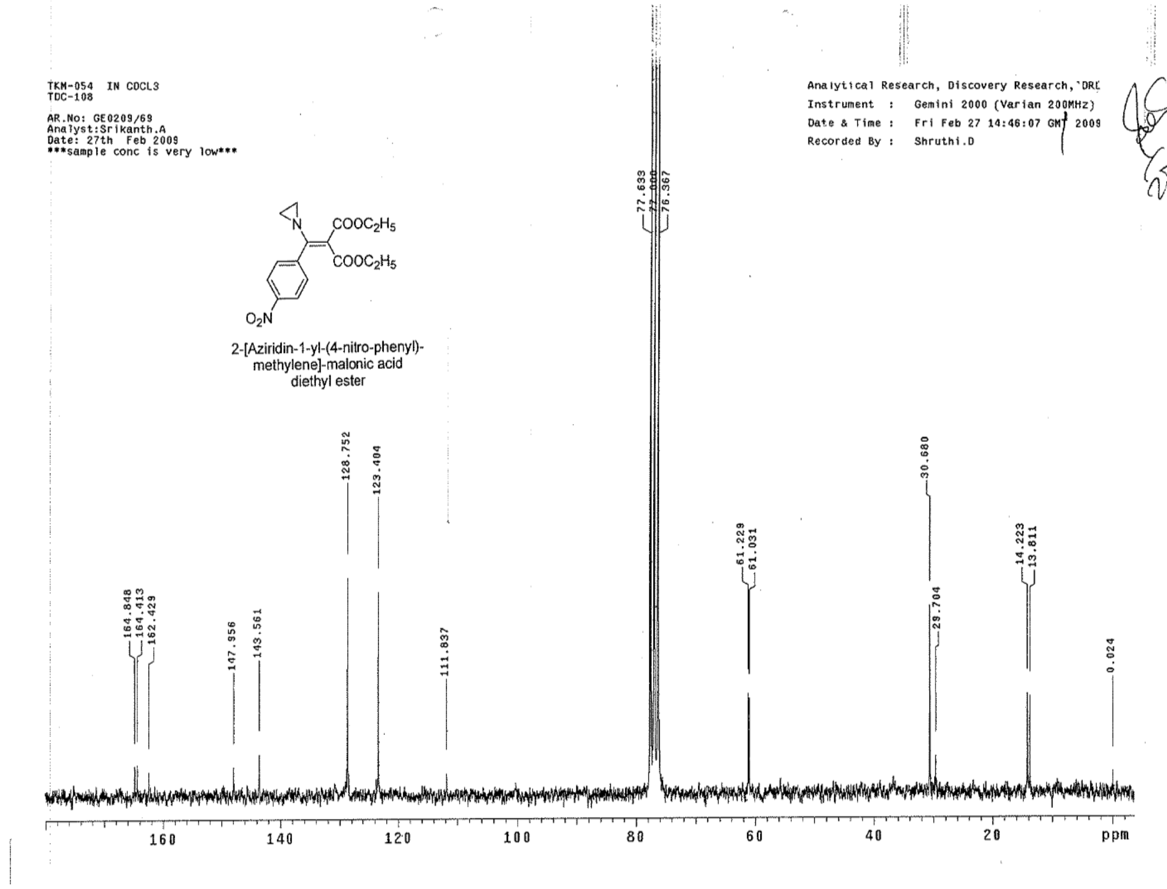

Mass spectrum of 20h

CPS,MIYAPUR

Mass Analysis Report

DR.REDDY'S

**Data Filename**	090218004.d	**Sample Name**	TKM-054
**Sample Type**	Sample	**Position**	Vial 4
**Instrument Name**	Instrument 1	**User Name**	
**Acq Method**		**IRM Calibration Status**	Success
**DA Method**	DA.m	**Comment**	

User Spectra

Fragmentor Voltage	Collision Energy	Ionization Mode
70	0	Esi

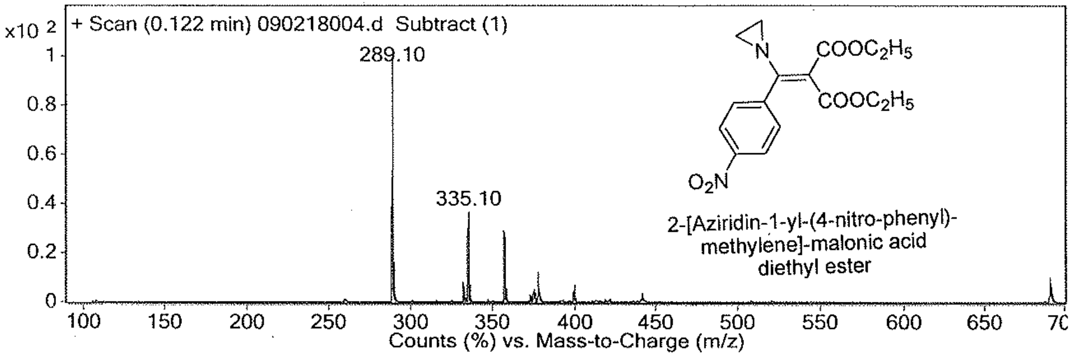

--- End Of Report ---

IR spectrum of 20h

DR.REDDY'S LABORATORIES LIMITED

TDC/CCS-ANALYTICAL RESEARCH

Date: 3/3/09

Time: 5:27:31 PM

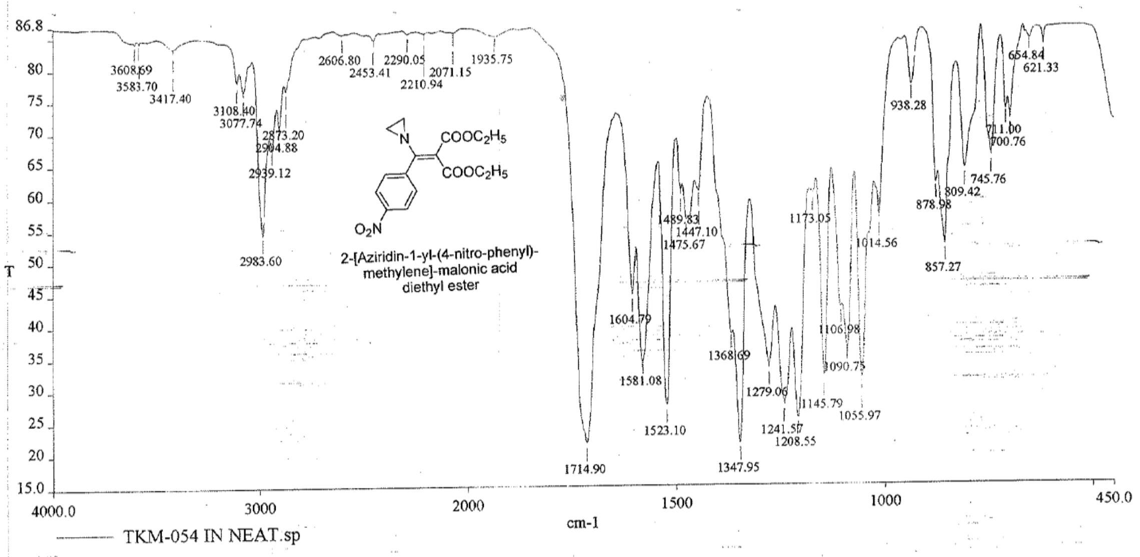

Analyst: SHYAM

SIGN:

03/03/09

AR090069/SAM/004
