# Supplementary material for: Synthesis of novel 5-alkyl/aryl/heteroaryl substituted diethyl 3,4-dihydro-2H-pyrrole-4,4-dicarboxylates by aziridine ring expansion of 2-[(aziridin-1-yl)-1-alkyl/aryl/heteroaryl-methylene]malonic acid diethyl esters
Source: Beilstein J Org Chem. 2011 Jun 20;7:831–8. doi: 10.3762/bjoc.7.95 (PMC3135249; doi:10.3762/bjoc.7.95)
Supplement: File 2 — 1H NMR, 13C NMR, IR, and mass spectra of 21b, 21d, 21g, 21h, 21i, 21j, 23, 24, 28, 29, 31, 32 and HRMS spectra of 21a–21j, 23, 24, 29, and 32. [file Beilstein_J_Org_Chem-07-831-s002.pdf]

Supporting Information

for

Synthesis of novel 5-alkyl/aryl/heteroaryl substituted diethyl 2*H*-pyrrole-4,4(3*H*)-dicarboxylates by aziridine ring expansion of 2-[(aziridin-1-yl)-1-alkyl/aryl/heteroaryl-methylene]malonic acid diethyl esters

Satish S. More*[1], T. Krishna Mohan[1], Y. Sateesh Kumar[1], U. K. Syam Kumar[1] and Navin B. Patel[2]

Address: [1]Technology Development centre, Custom Pharmaceutical Services, Dr. Reddy's Laboratories Ltd., Miyapur, Hyderabad -500 049, India and [2]Department of Chemistry, Veer Narmad South Gujarat University, Surat, India

Email: Satish S. More* - satishsm@drreddys.com

* Corresponding author

^{1}H NMR, ^{13}C NMR, IR, and mass spectra of **21b**, **21d**, **21g**, **21h**, **21i**, **21j**, **23**, **24**, **28**, **29**, **31**, **32** and HRMS spectra of **21a–21j**, **23**, **24**, **29**, and **32.**

Table of contents

	Content	Page
1.	Spectra (^{1}H NMR, ^{13}C NMR, MS and IR) **21b**, **21d**, **21g**, **21h**, **21i**, **21j**, **23**, **24**, **28**, **29**, **31**, and **32**	S2
2.	HRMS spectra of **21a–21j**, **23**, **24**, **29**, **32**	S26

^{1}H NMR spectrum of 21b

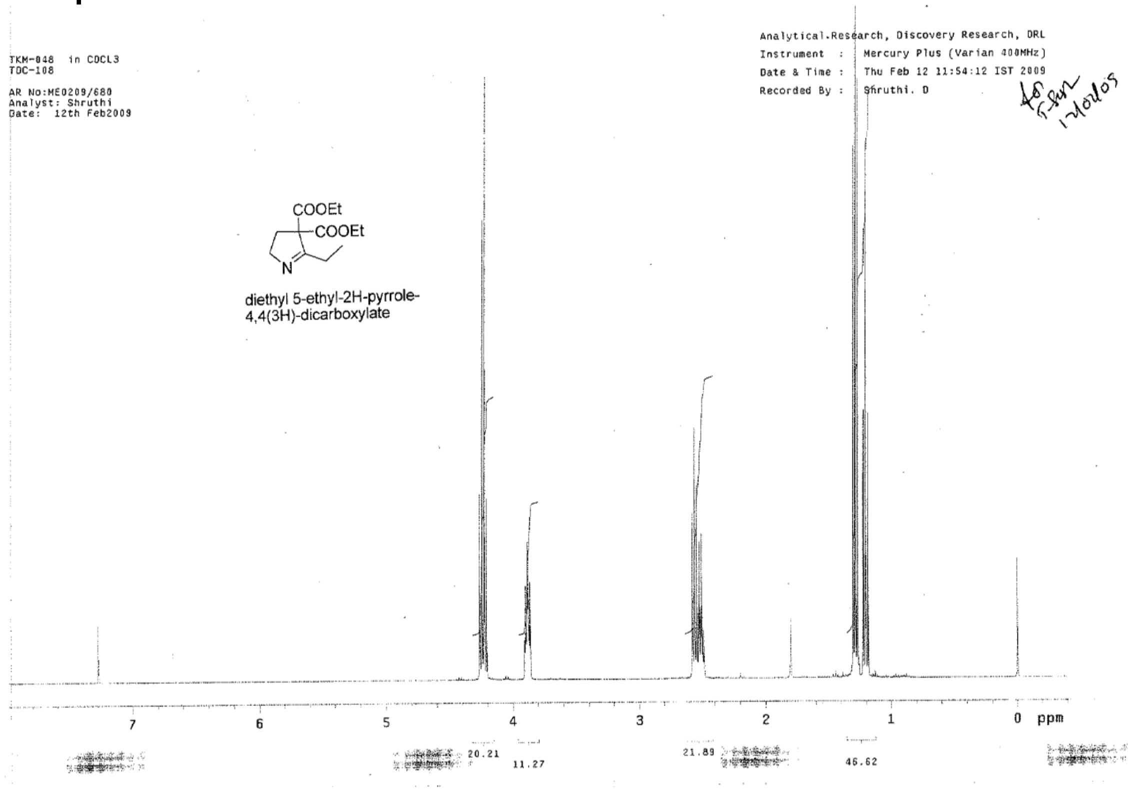

^{13}C NMR spectrum of 21b

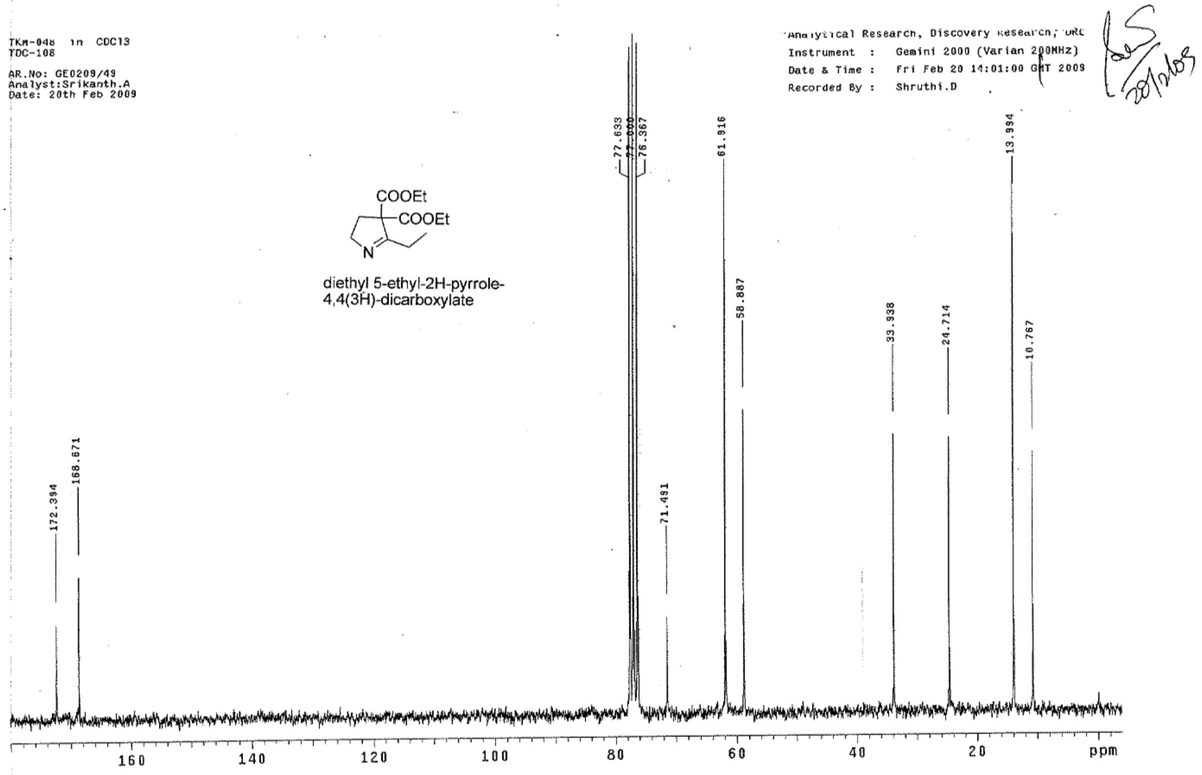

Mass spectrum of 21b

CPS,MIYAPUR

Mass Analysis Report

**Data Filename**	090206004.d	**Sample Name**	TKM-048
**Sample Type**	Sample	**Position**	Vial 74
**Instrument Name**	Instrument 1	**User Name**	
**Acq Method**		**IRM Calibration Status**	Success
**DA Method**	DA.m	**Comment**	

User Spectra

Fragmentor Voltage	Collision Energy	Ionization Mode
70	0	Esi

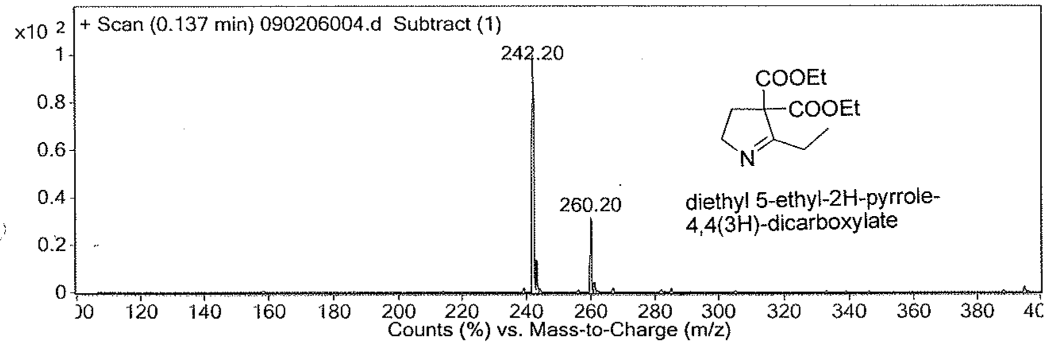

--- End Of Report ---

IR spectrum of 21b

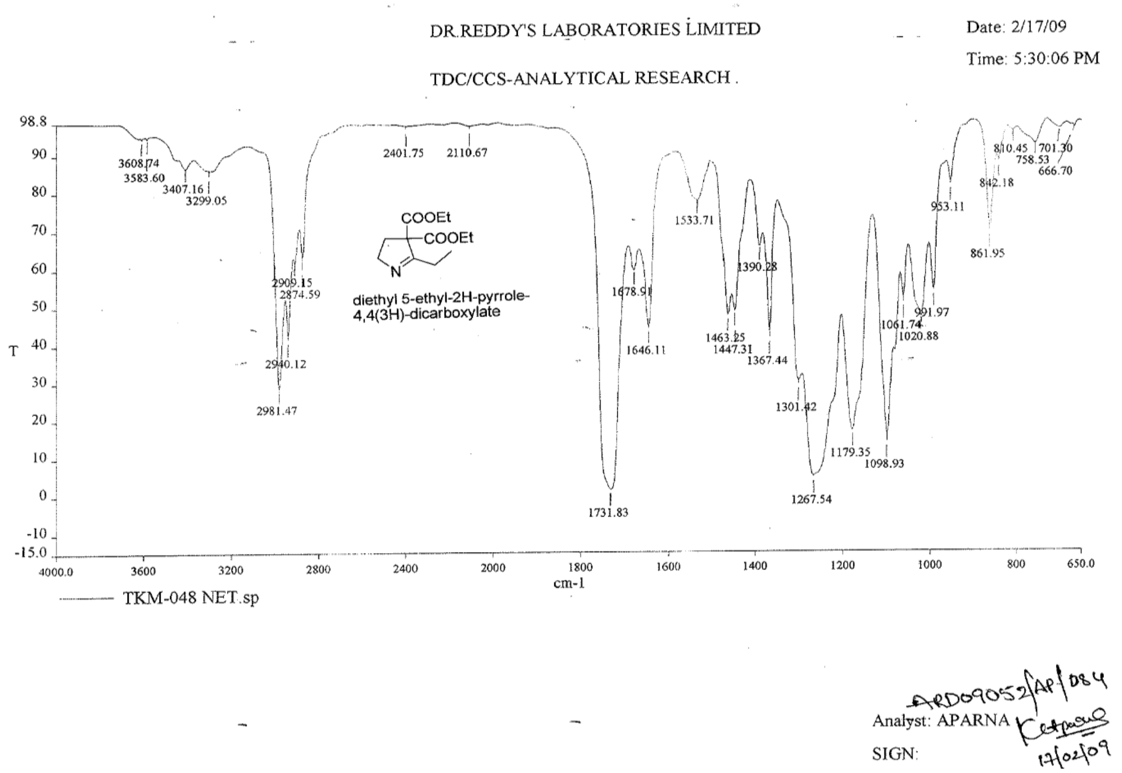

^{1}H NMR spectrum of 21d

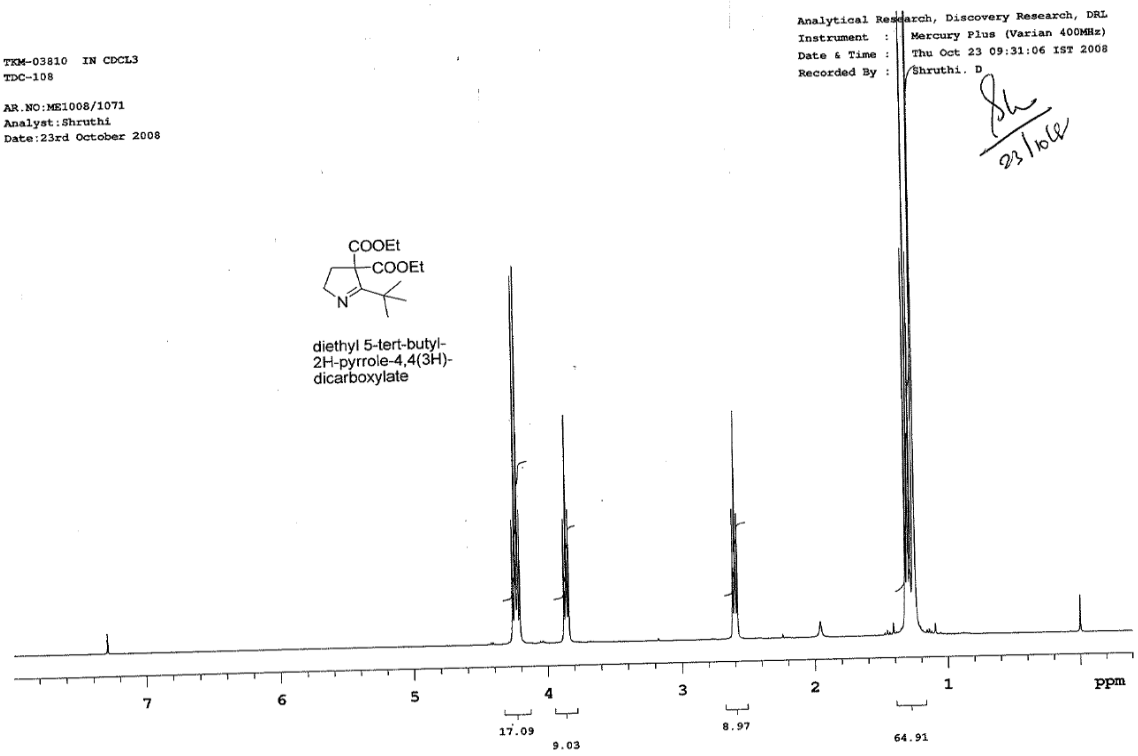

^{13}C NMR spectrum of 21d

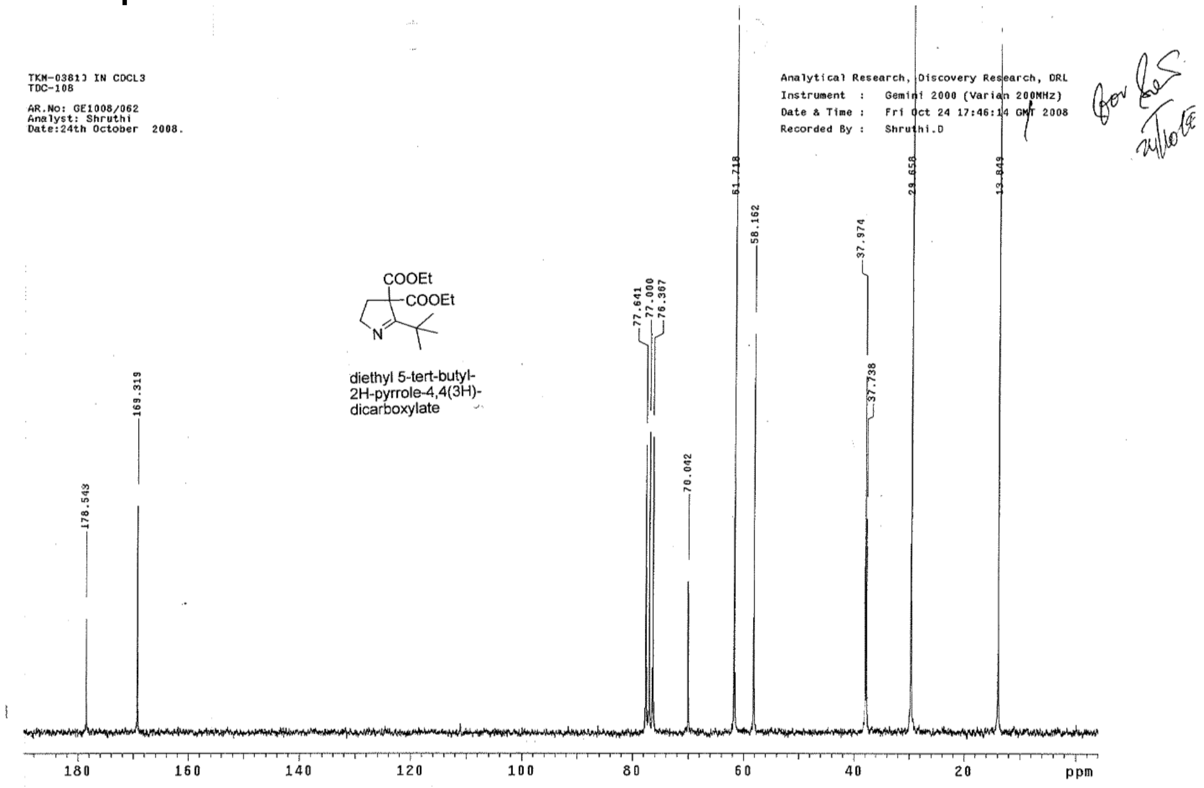

Mass Spectra of 21d

CPS,MIYAPUR

Mass Analysis Report

**Data Filename**	081022009.d	**Sample Name**	TKM-03810
**Sample Type**	Sample	**Position**	Vial 9
**Instrument Name**	Instrument 1	**User Name**	
**Acq Method**		**IRM Calibration Status**	Success
**DA Method**	DA.m	**Comment**	

User Spectra

Fragmentor Voltage	Collision Energy	Ionization Mode
135	0	Esi

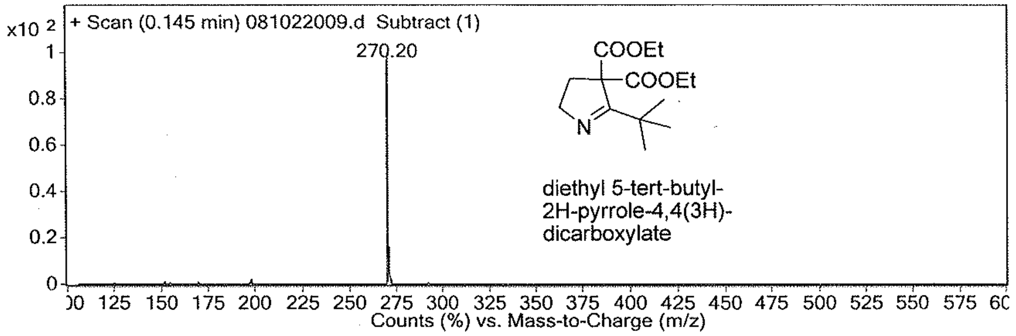

--- End Of Report ---

IR spectrum of 21d

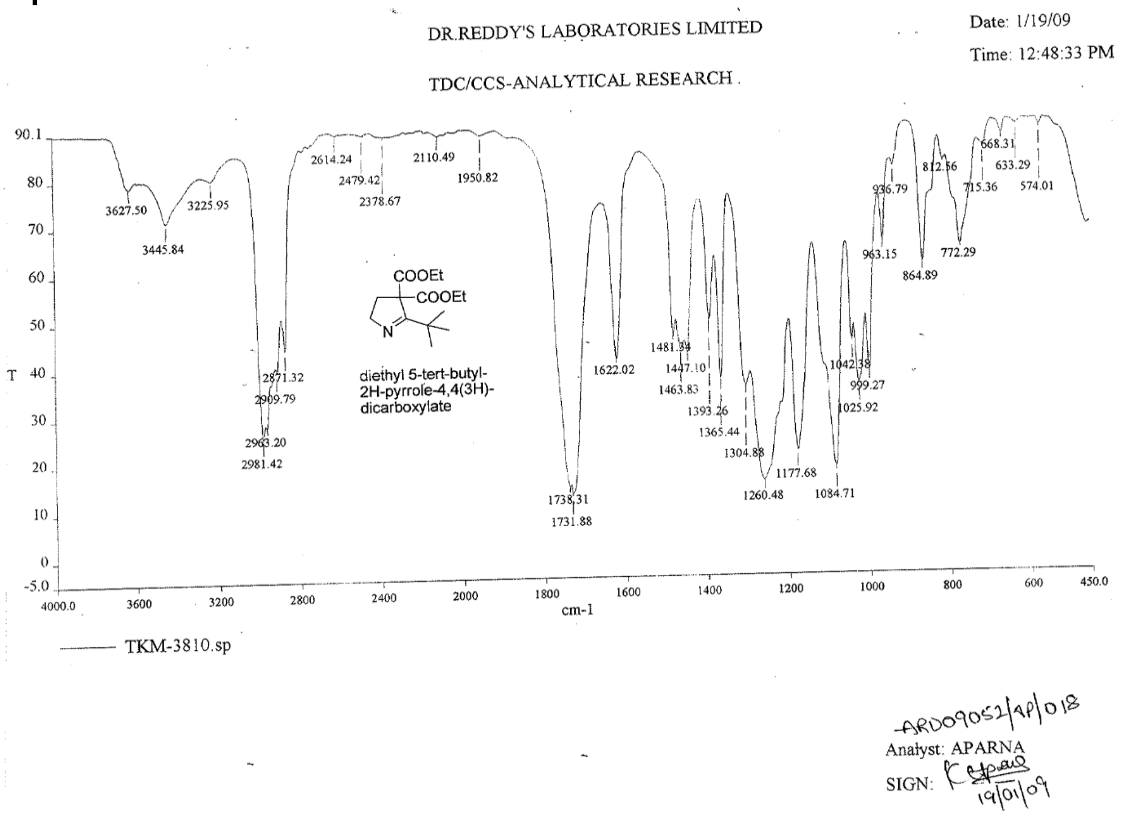

^{1}H NMR spectrum of 21g

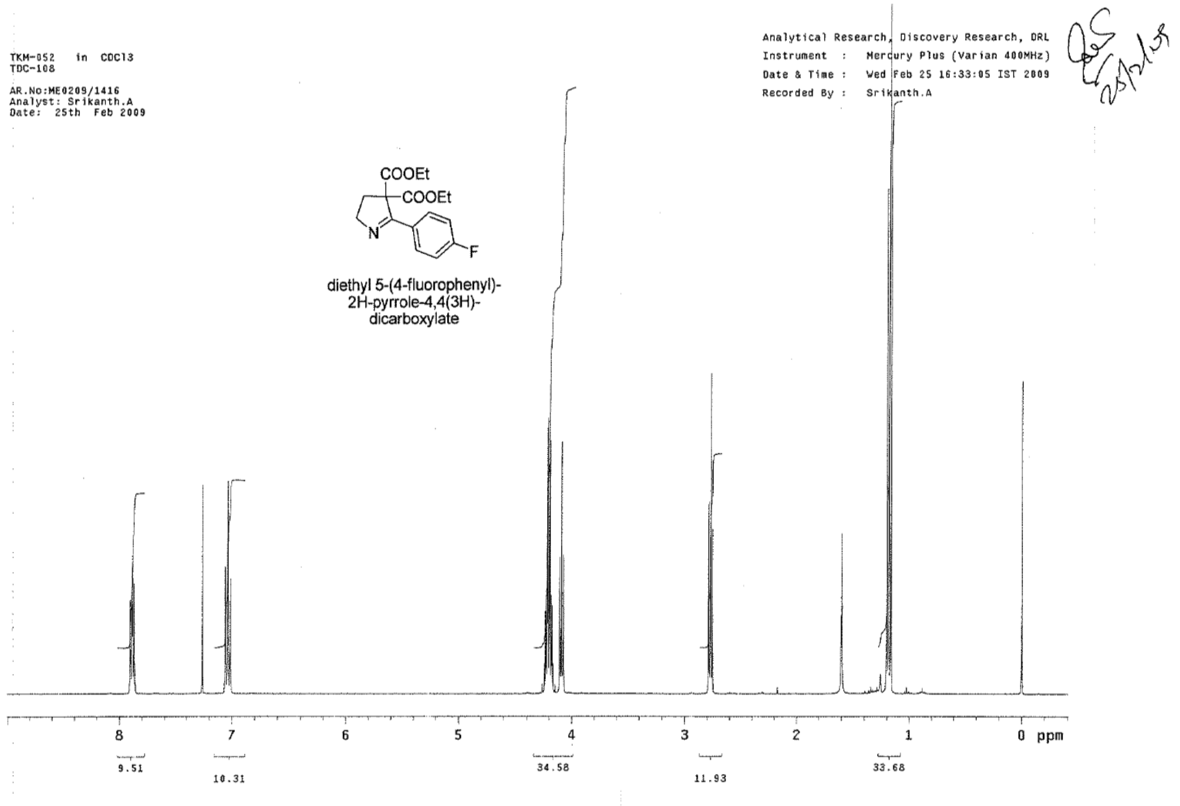

^{13}C NMR spectrum of 21g

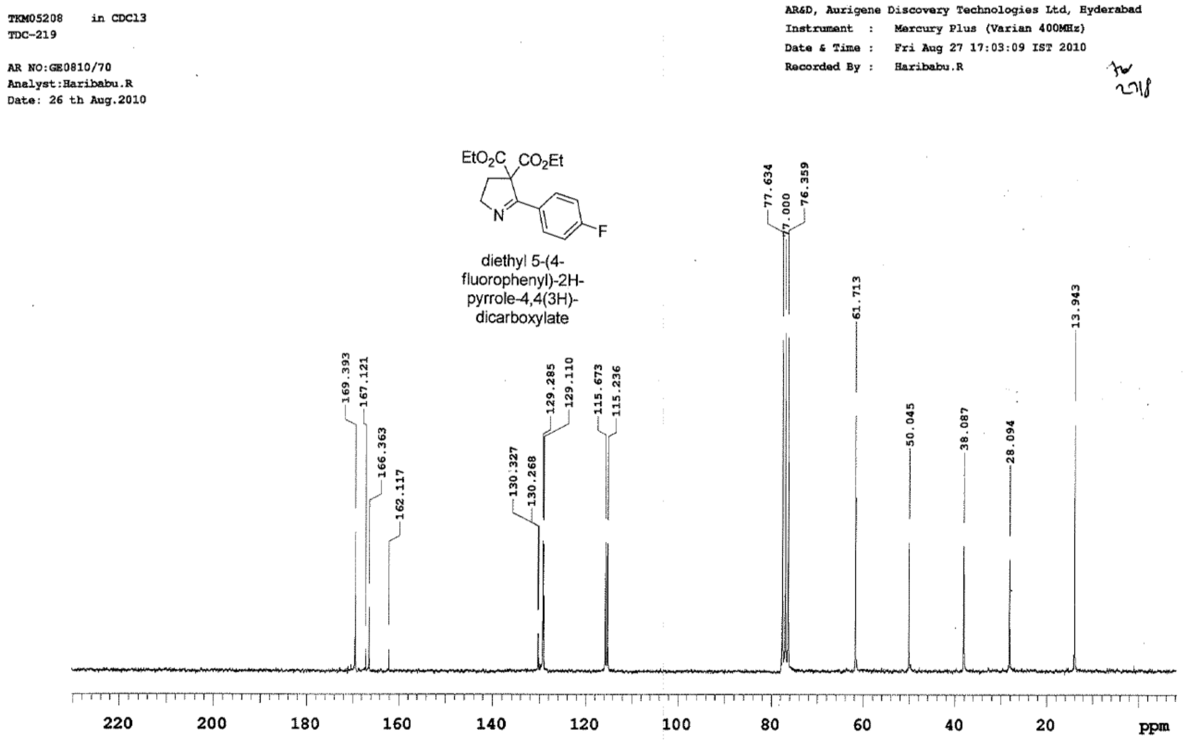

Mass spectrum of 21g

CPS,MIYAPUR

Mass Analysis Report

**Data Filename**	090250017.d	**Sample Name**	TKM-052
**Sample Type**	Sample	**Position**	Vial 39
**Instrument Name**	Instrument 1	**User Name**	
**Acq Method**	ESI.m	**IRM Calibration Status**	Success
**DA Method**	DA.m	**Comment**	

User Spectra

Fragmentor Voltage	Collision Energy	Ionization Mode
70	0	ESI

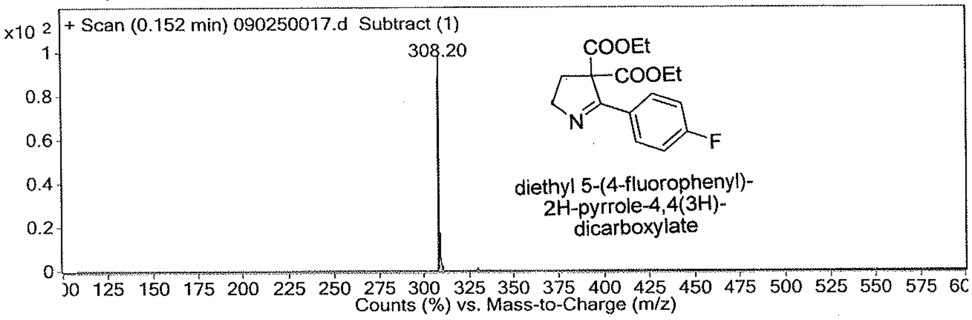

--- End Of Report ---

IR spectrum of 21g

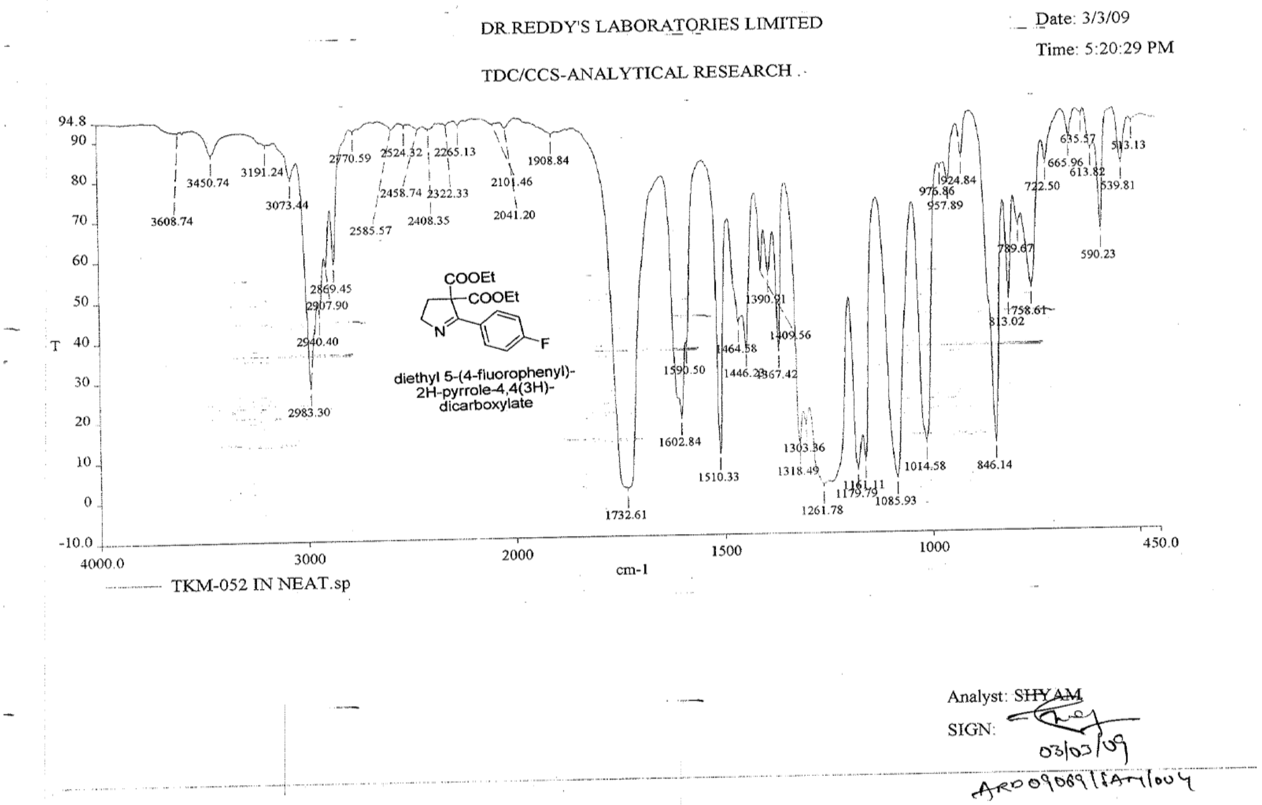

^{1}H NMR spectrum of 21h

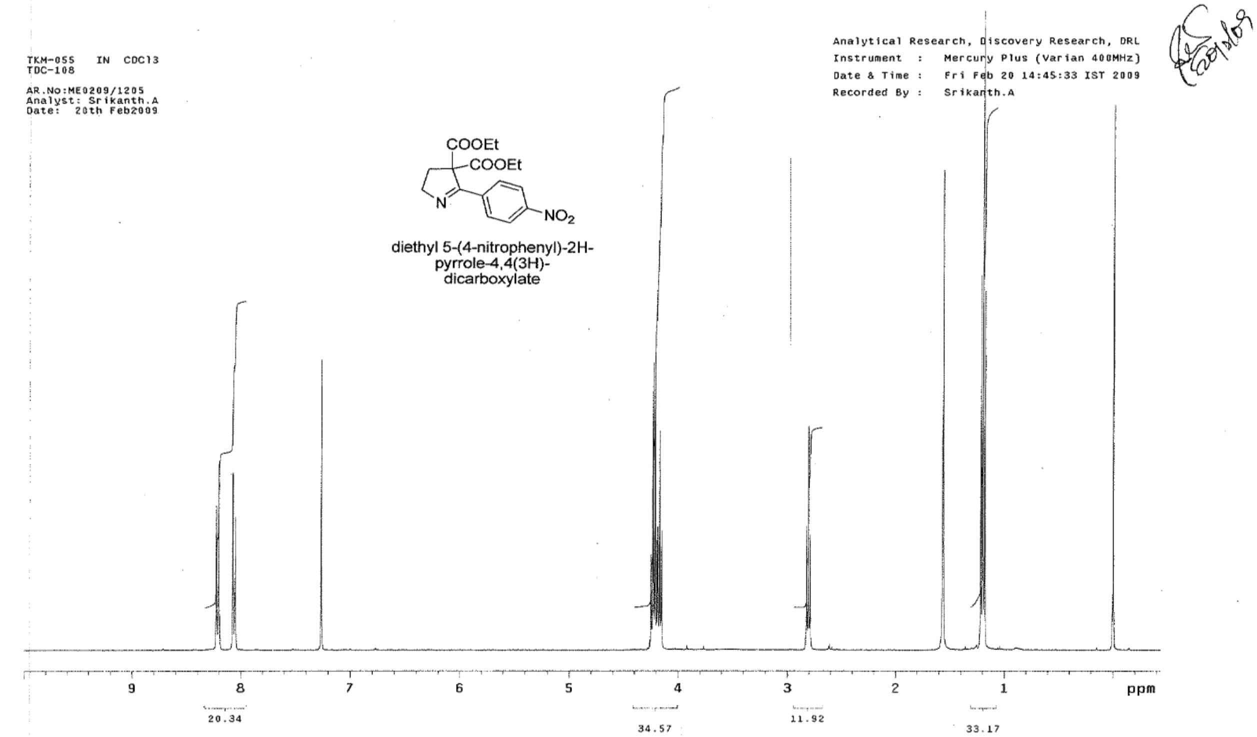

^{13}C NMR spectrum of 21h

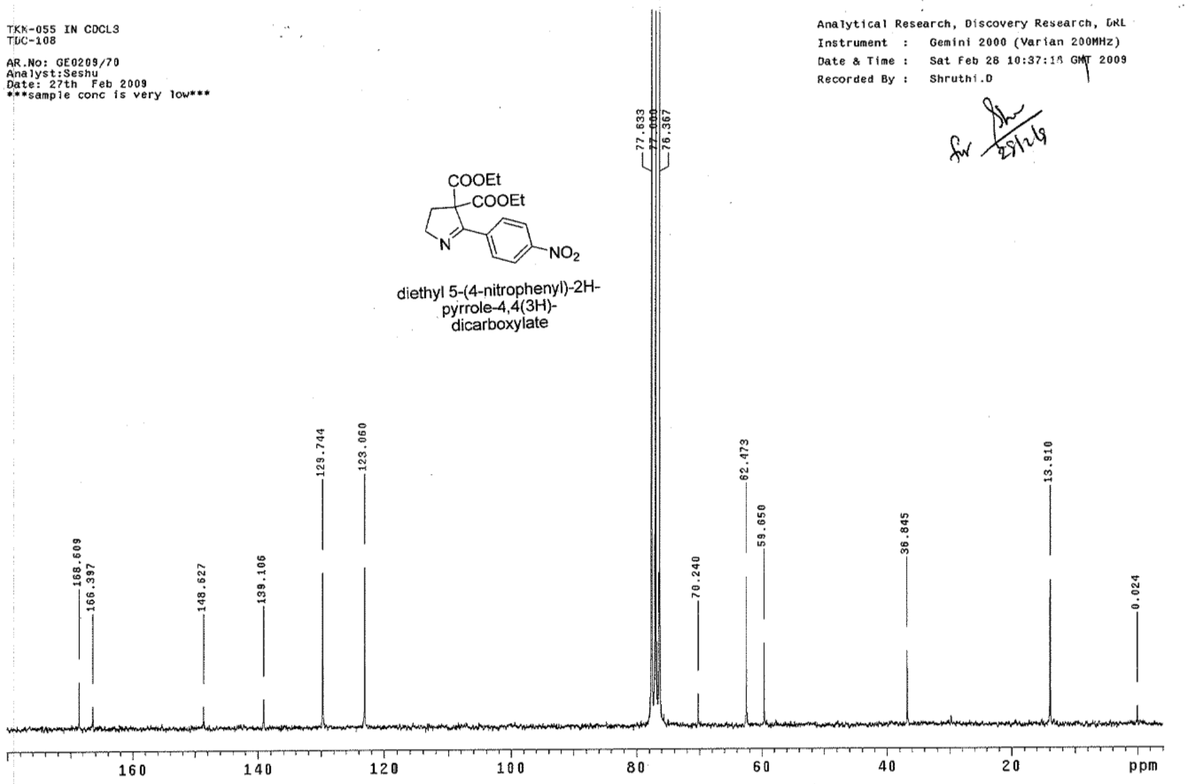

Mass spectrum of 21h

CPS,MIYAPUR

Mass Analysis Report

**Data Filename**	090220024.d	**Sample Name**	TKM-055
**Sample Type**	Sample	**Position**	Vial 22
**Instrument Name**	Instrument 1	**User Name**	
**Acq Method**	ESI.m	**IRM Calibration Status**	Success
**DA Method**	DA.m	**Comment**	

User Spectra

Fragmentor Voltage	Collision Energy	Ionization Mode
70	0	Esi

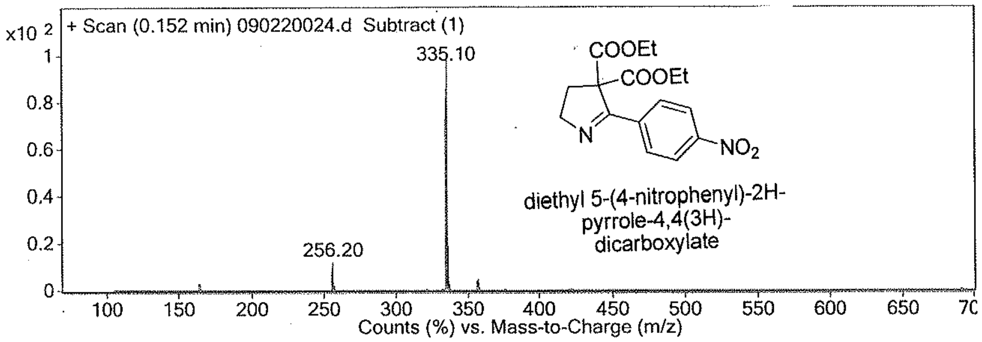

--- End Of Report ---

IR spectrum of 21h

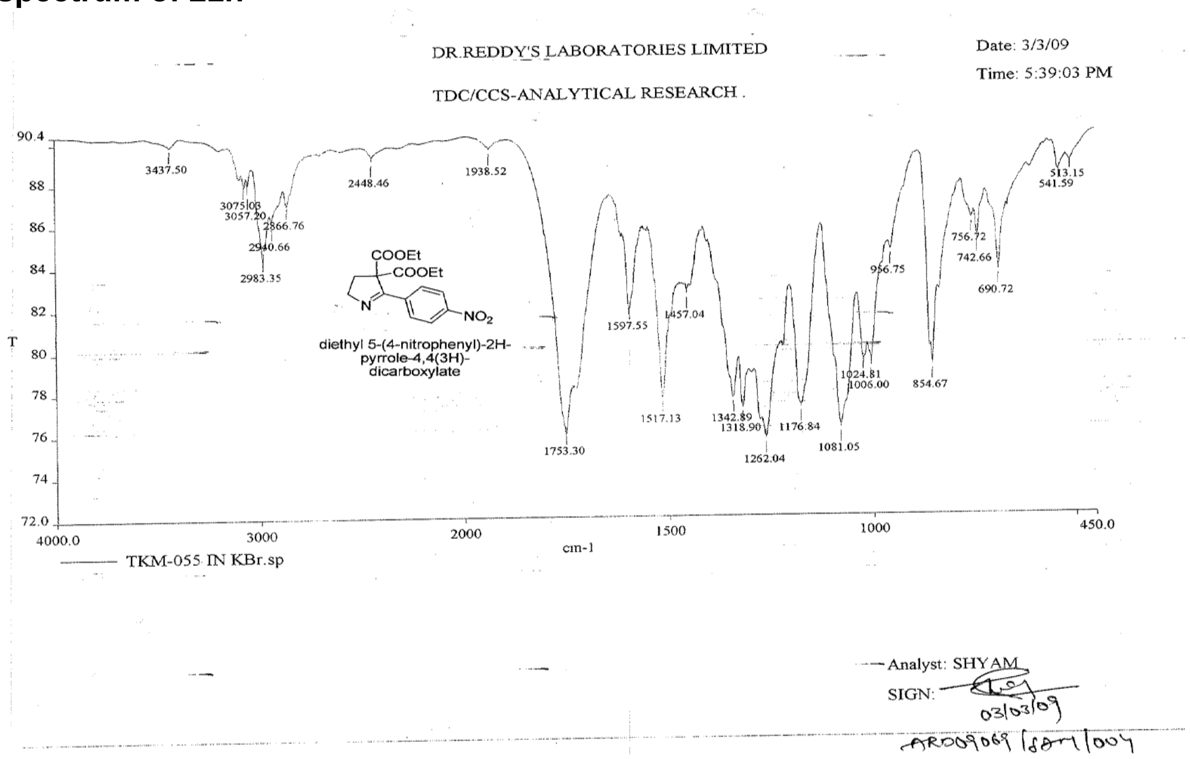

^{1}H NMR Spectra of 21i

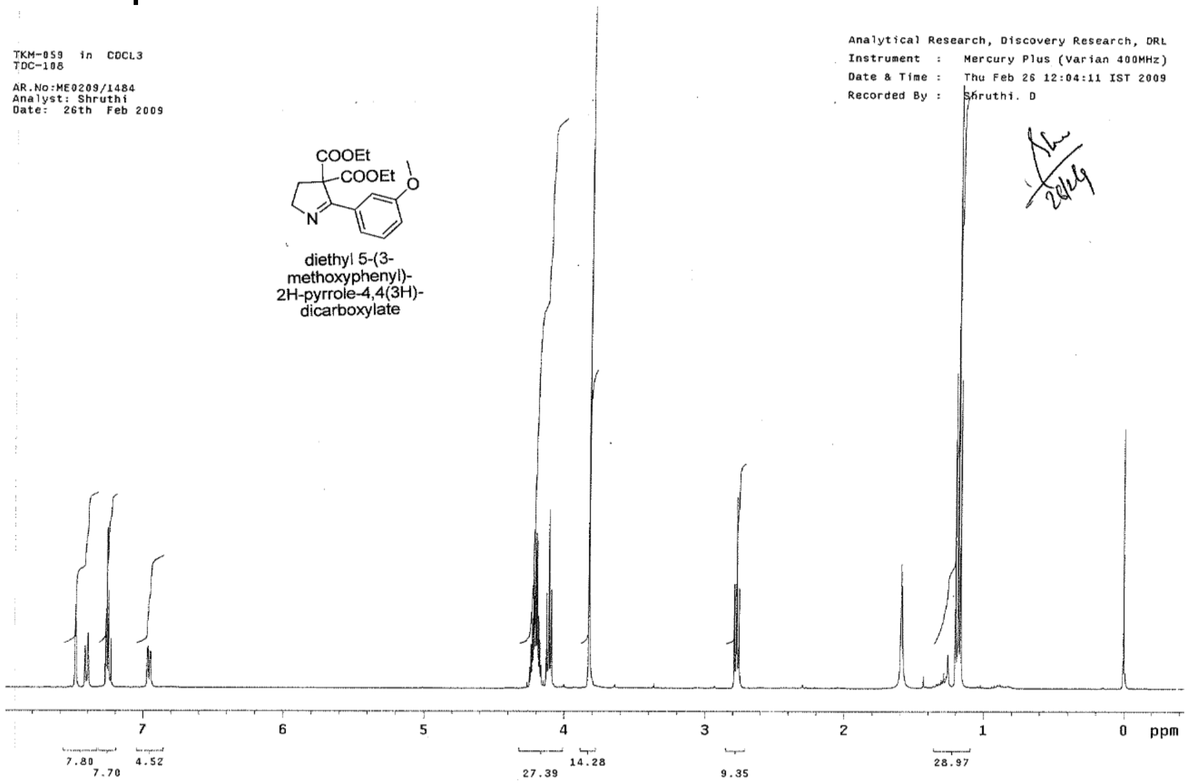

^{13}C NMR spectrum of 21i

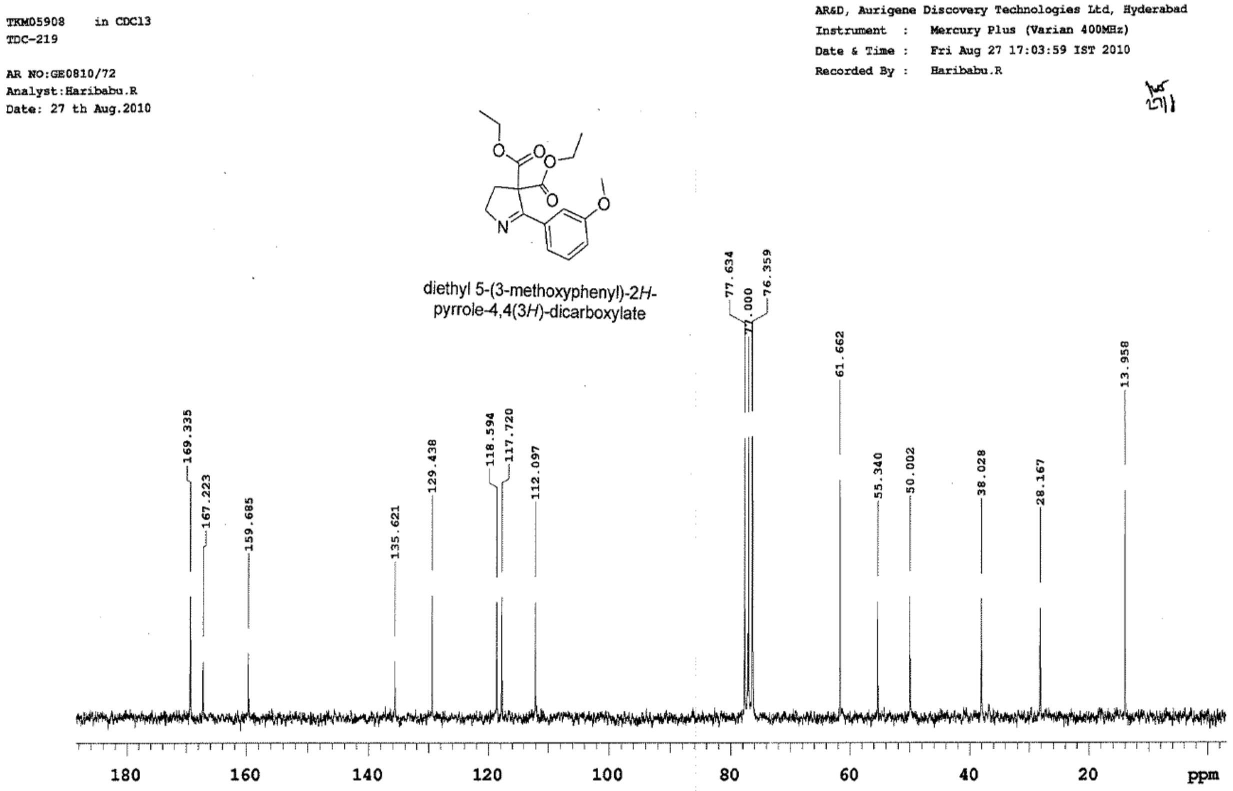

Mass spectrum of 21i

CPS,MIYAPUR

Mass Analysis Report

**Data Filename**	090226020.d	**Sample Name**	TKM-059
**Sample Type**	Sample	**Position**	Vial 30
**Instrument Name**	Instrument 1	**User Name**	
**Acq Method**	ESI.m	**IRM Calibration Status**	Success
**DA Method**	Amscrine.m	**Comment**	

User Spectra

Fragmentor Voltage	Collision Energy	Ionization Mode
70	0	Esi

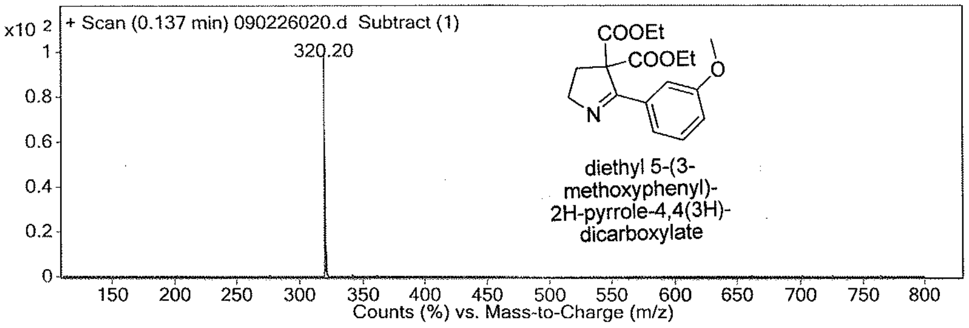

--- End Of Report ---

IR spectrum of 21i

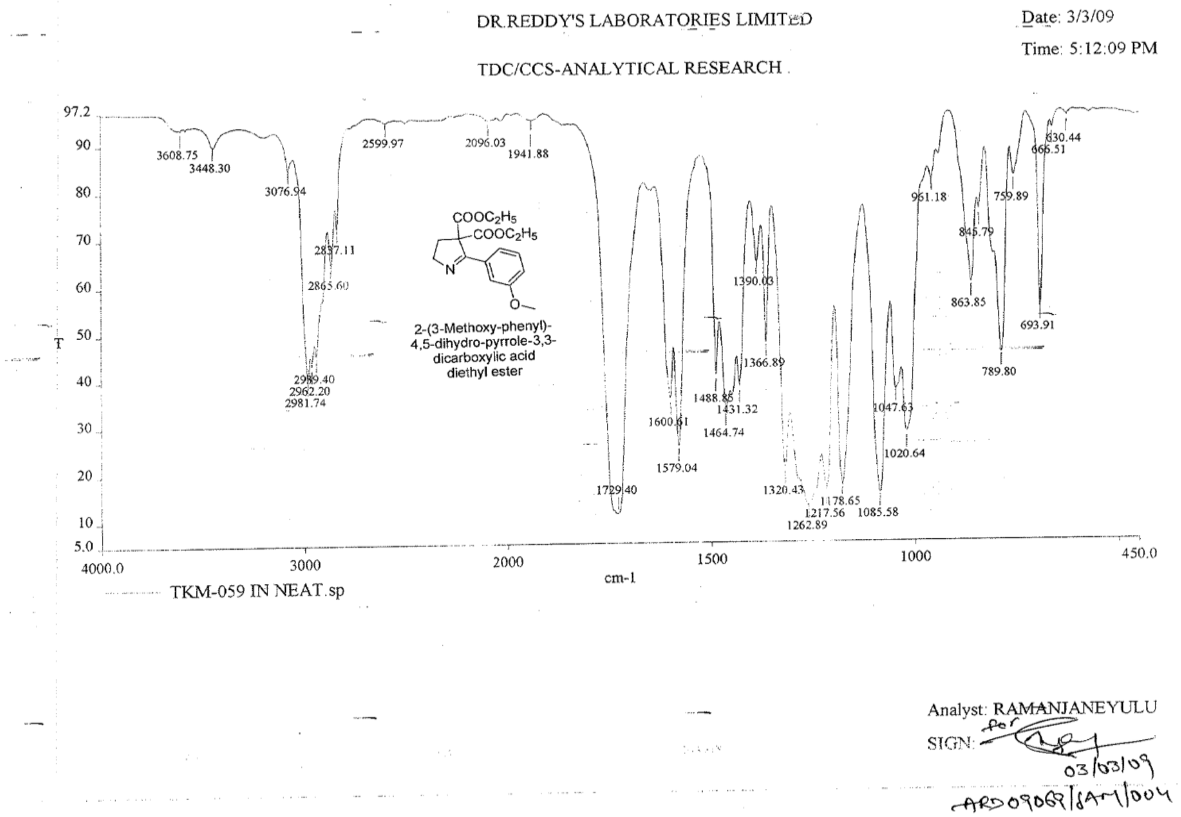

^{1}H NMR spectrum of 21j

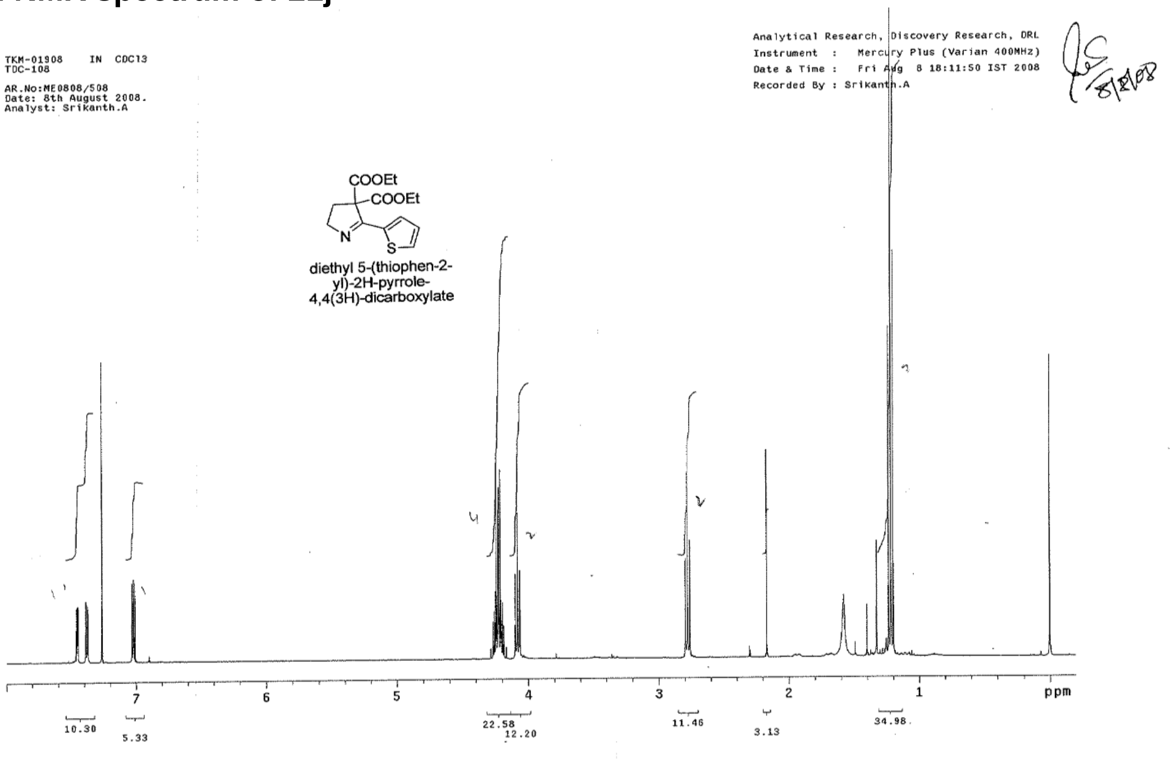

^{13}CNMR spectrum of 21j

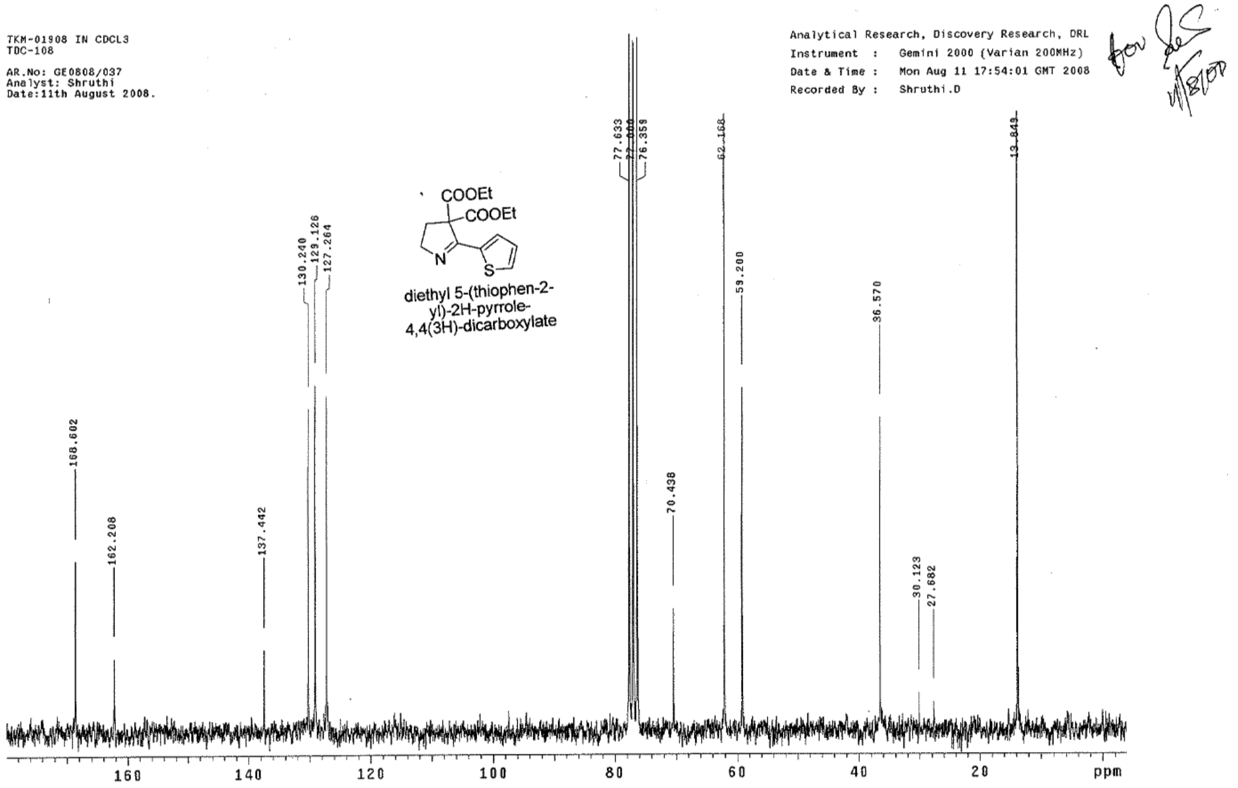

Mass spectrum of 21j

CPS,MIYAPUR

Mass Analysis Report

**Data Filename**	080811027.d	**Sample Name**	TKM-019-08
**Sample Type**	Sample	**Position**	Vial 86
**Instrument Name**	Instrument 1	**User Name**	
**Acq Method**		**IRM Calibration Status**	Success
**DA Method**	DA.m	**Comment**	

User Spectra

Fragmentor Voltage	Collision Energy	Ionization Mode
135	0	ESI

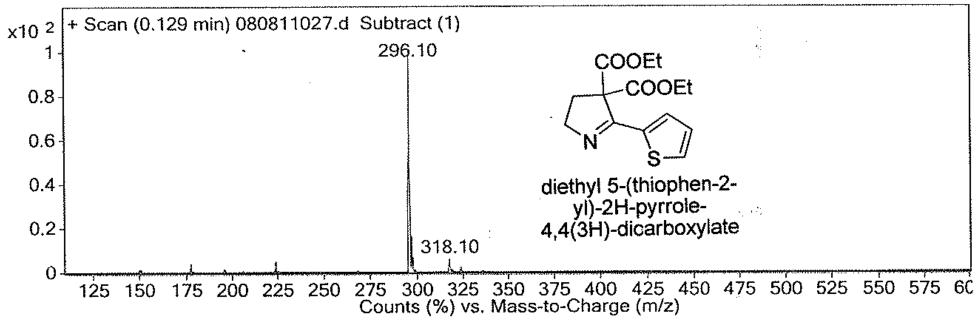

--- End Of Report ---

IR spectrum of 21j

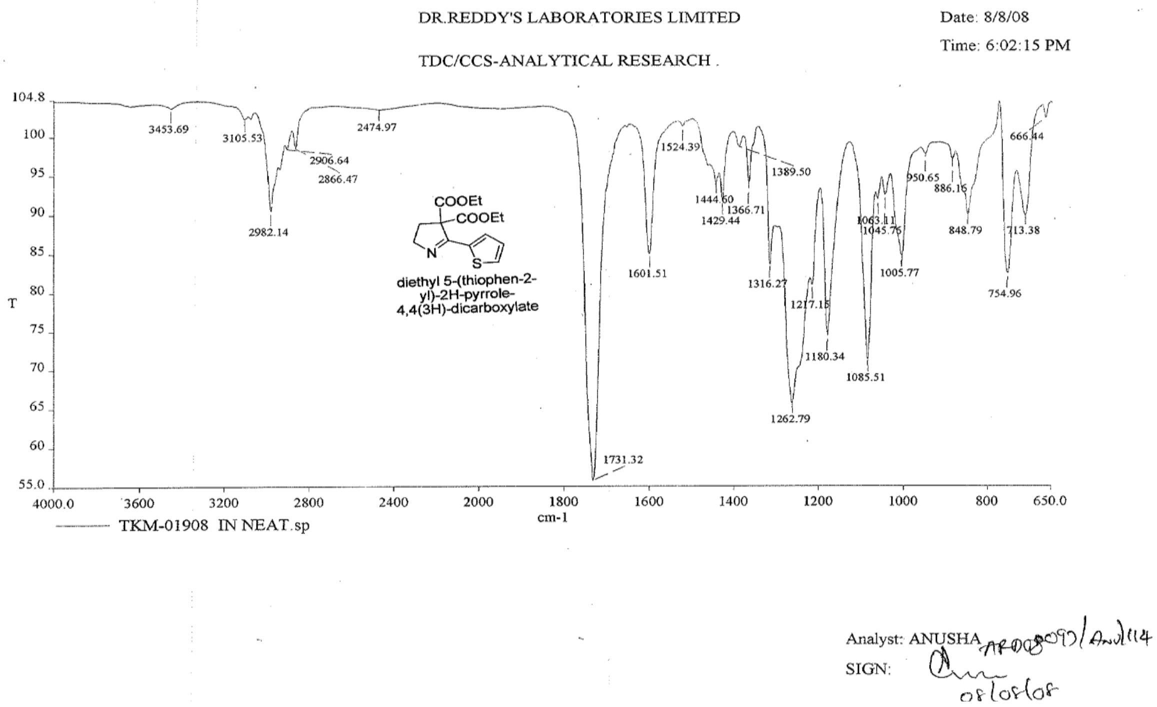

^{1}H NMR spectrum of 23

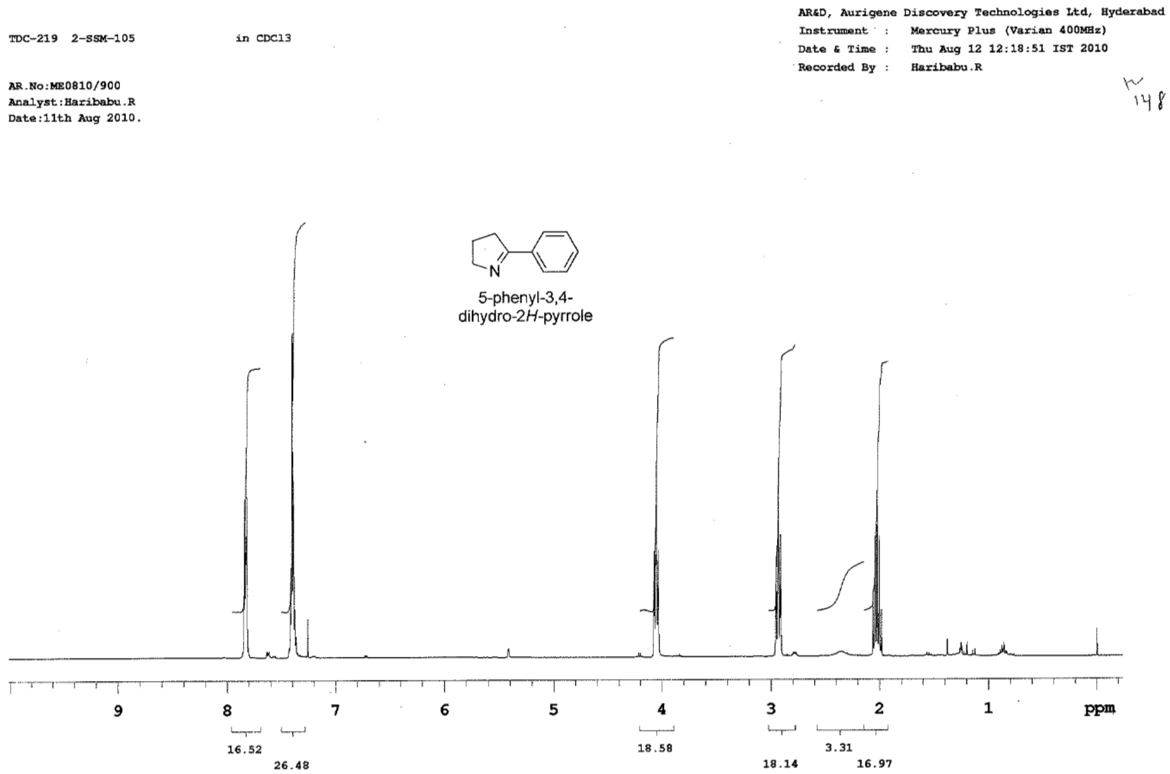

^{13}C NMR spectrum of 23

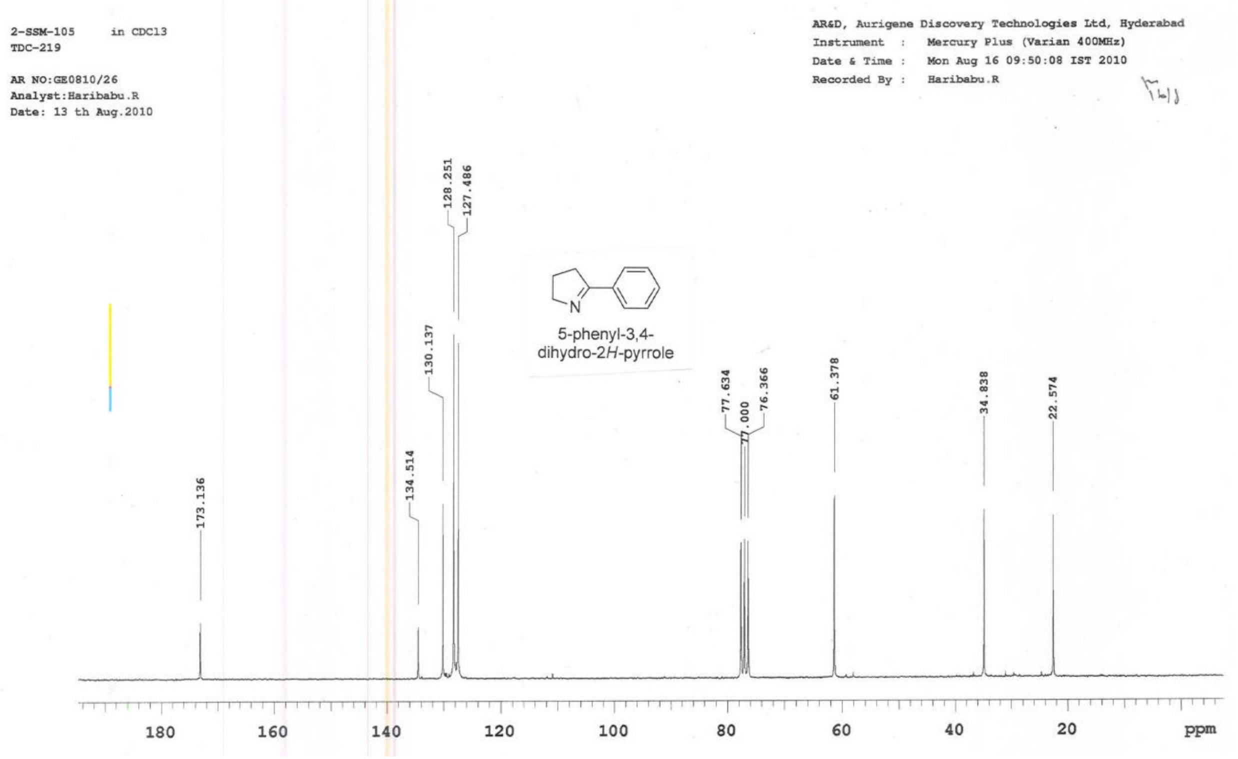

Mass spectrum of 23

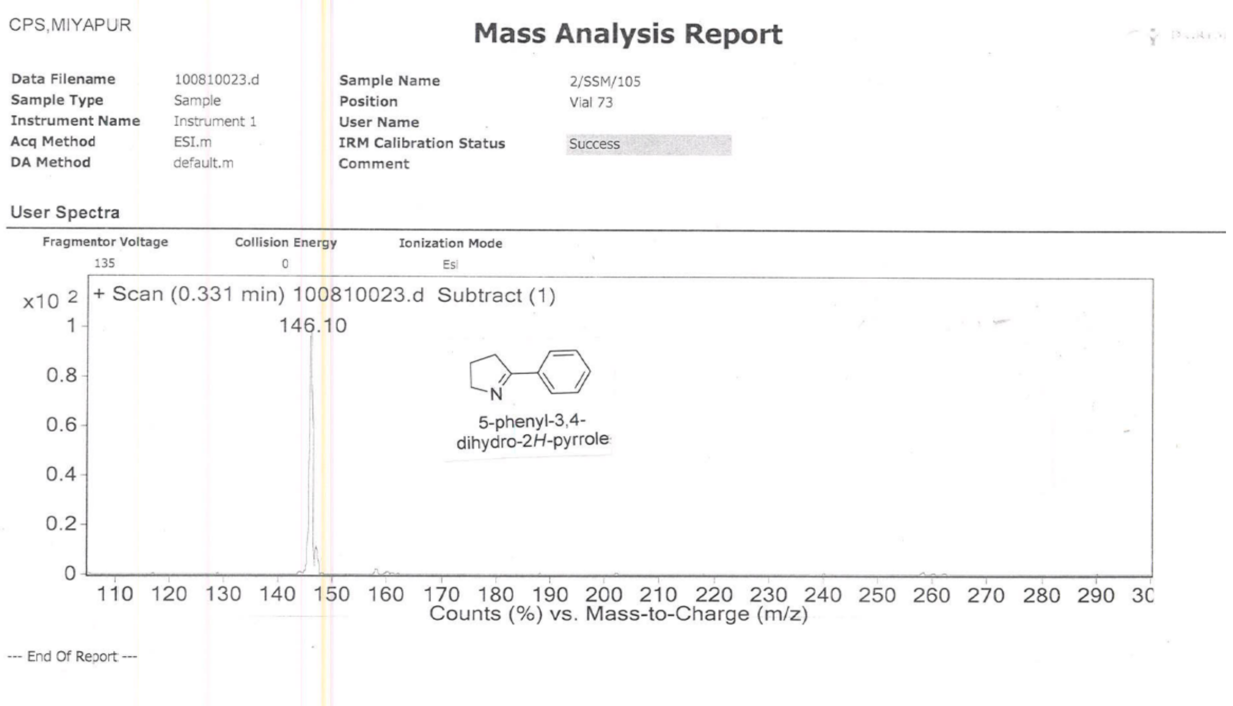

IR spectrum of 23

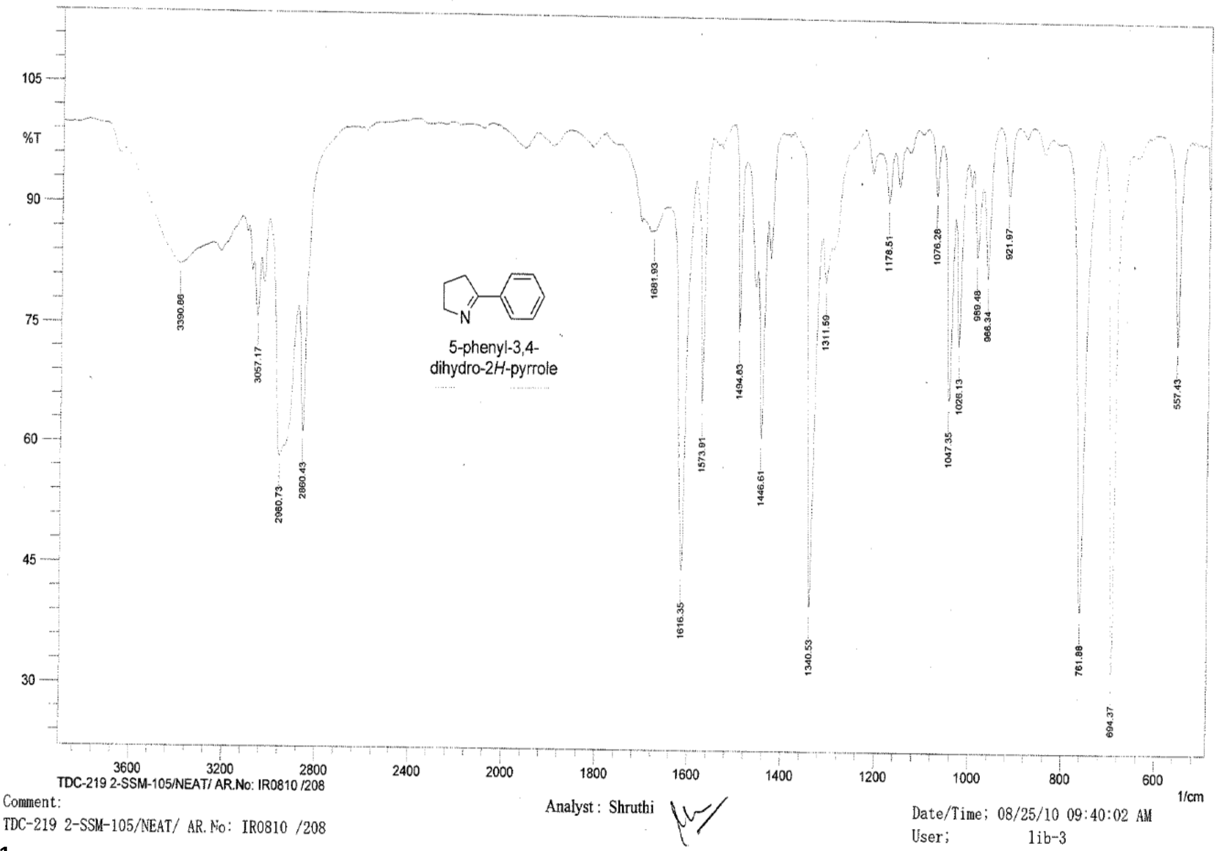

^{1}H NMR spectrum of 24

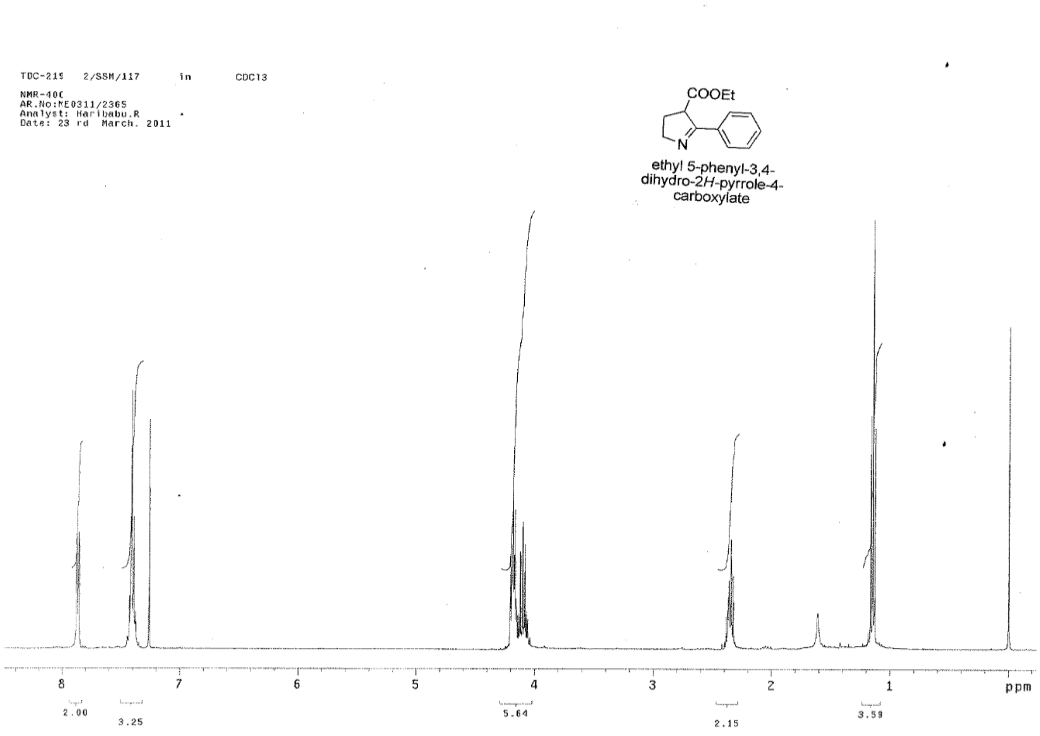

^{13}C NMR spectrum of 24

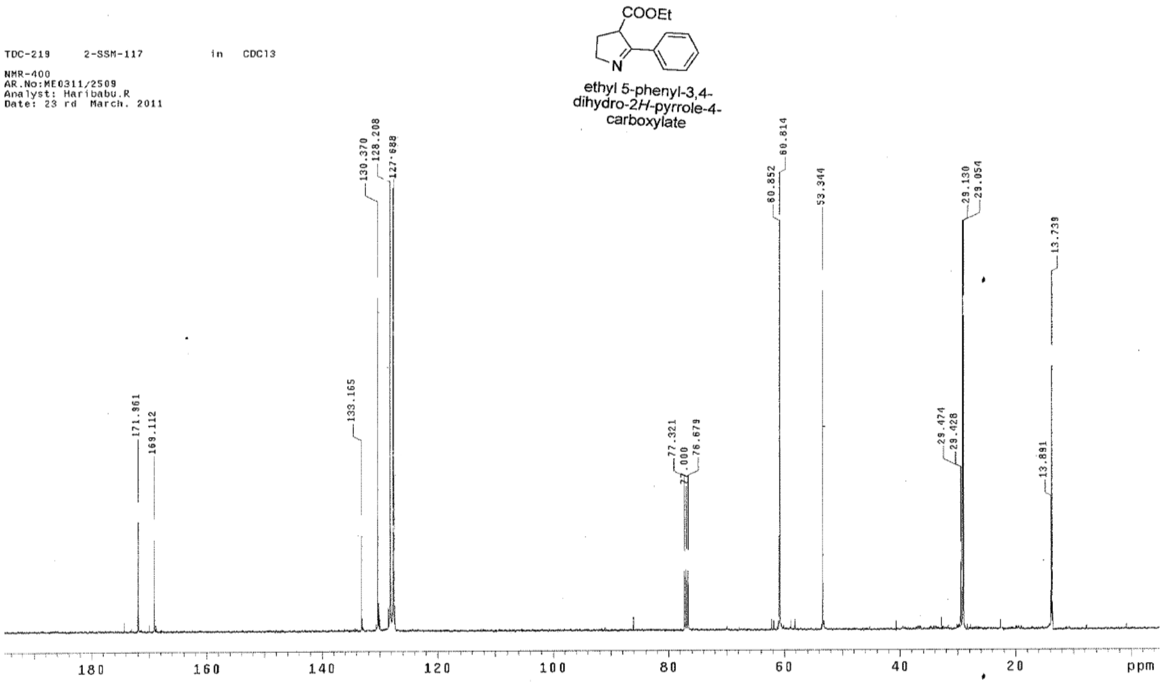

Mass spectrum of 24

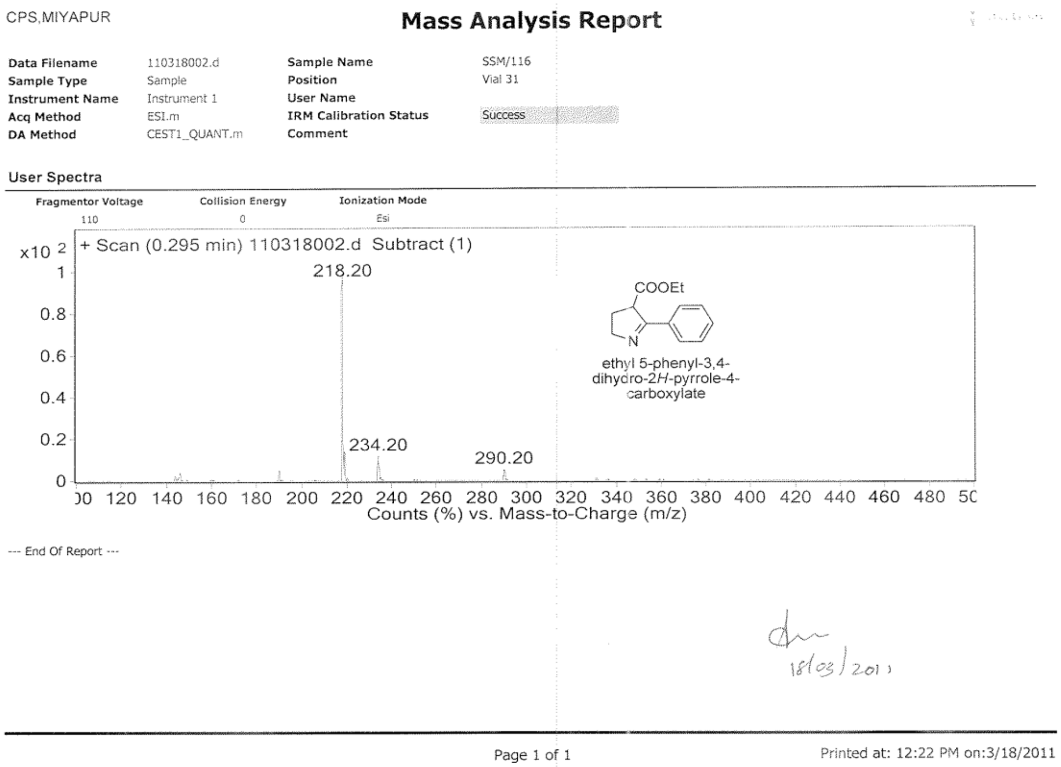

IR spectrum of 24

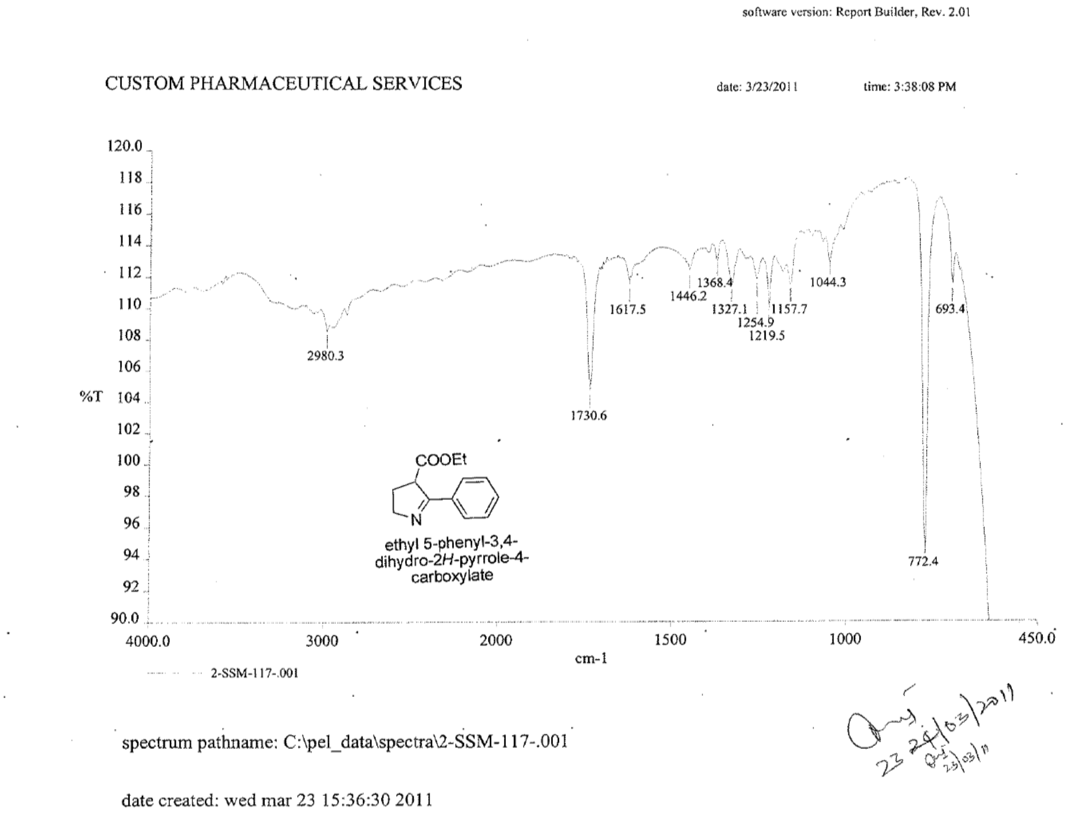

^{1}H NMR spectrum of 28

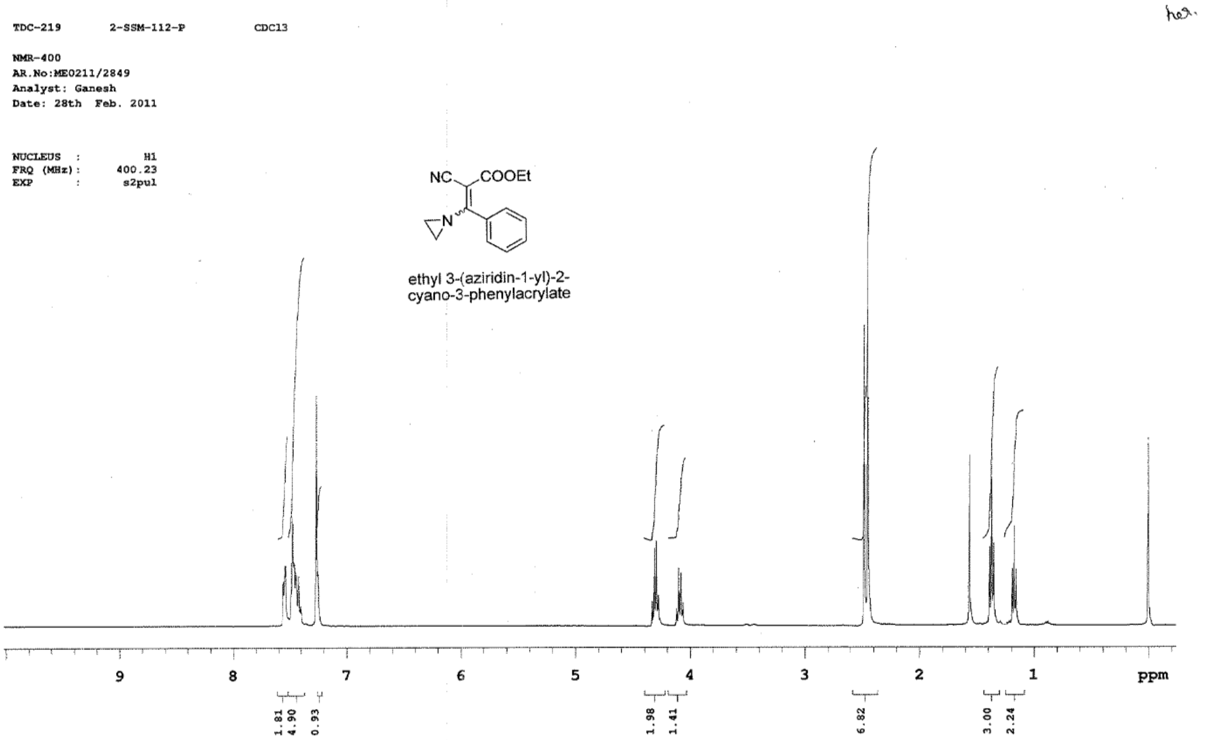

^{13}C NMR spectrum of 28

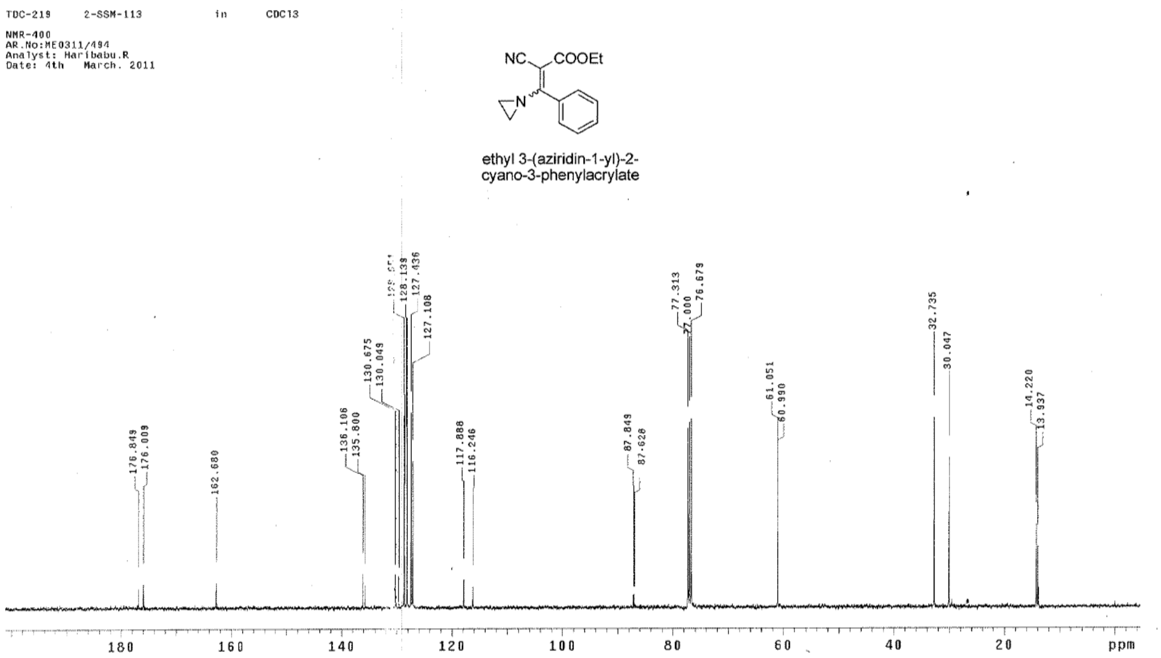

Mass spectrum of 28

CPS,MIYAPUR

Mass Analysis Report

**Data Filename**	110228003.d	**Sample Name**	2/SSM/112-Crude
**Sample Type**	Sample	**Position**	Vial 53
**Instrument Name**	Instrument 1	**User Name**	
**Acq Method**	ESI.m	**IRM Calibration Status**	Success
**DA Method**	CDD-MRM.m	**Comment**	

User Spectra

Fragmentor Voltage	Collision Energy	Ionization Mode
135	0	Esi

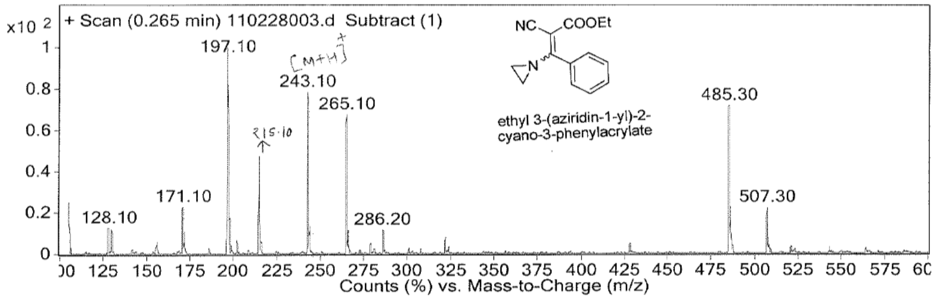

--- End Of Report ---

28/02/2011

Page 1 of 1 Printed at: 9:35 AM on:2/28/2011

IR spectrum of 28

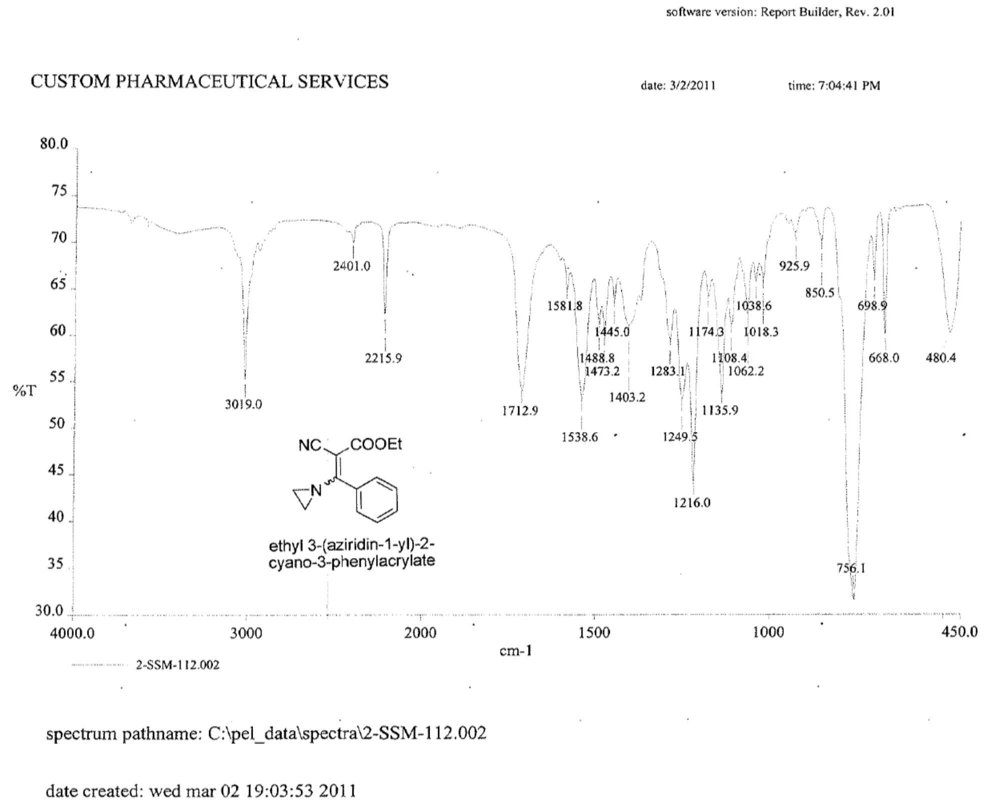

^{1}H NMR spectrum of 29

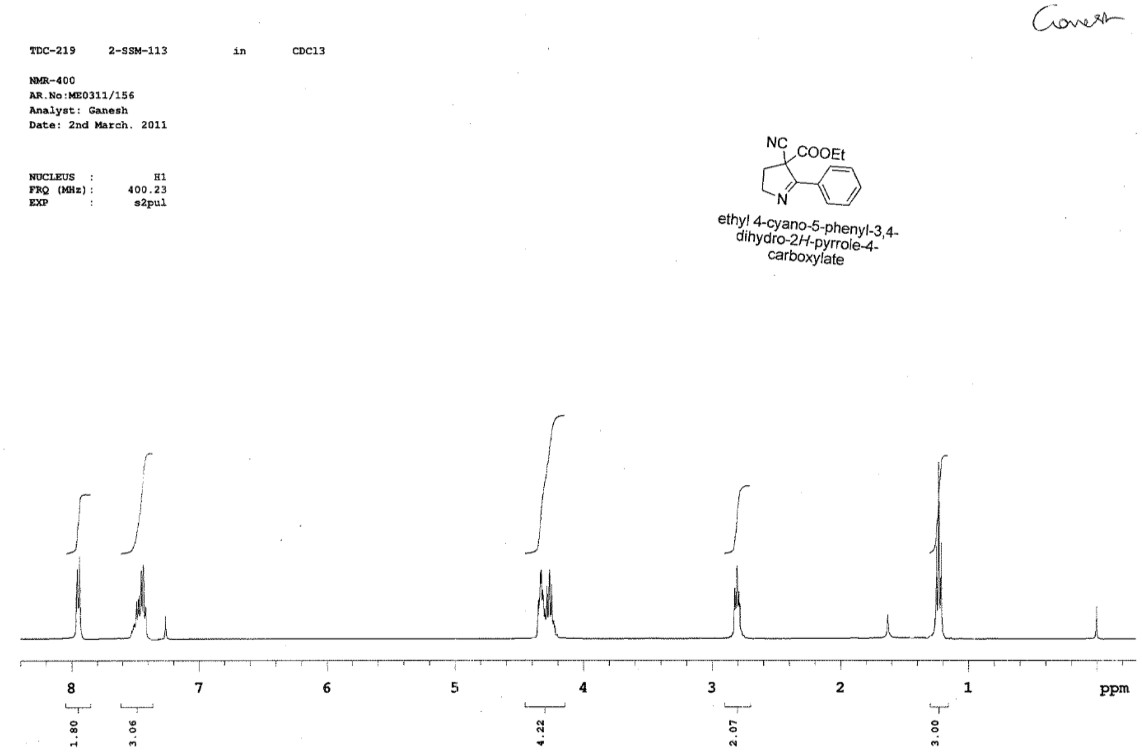

^{13}C NMR spectrum of 29

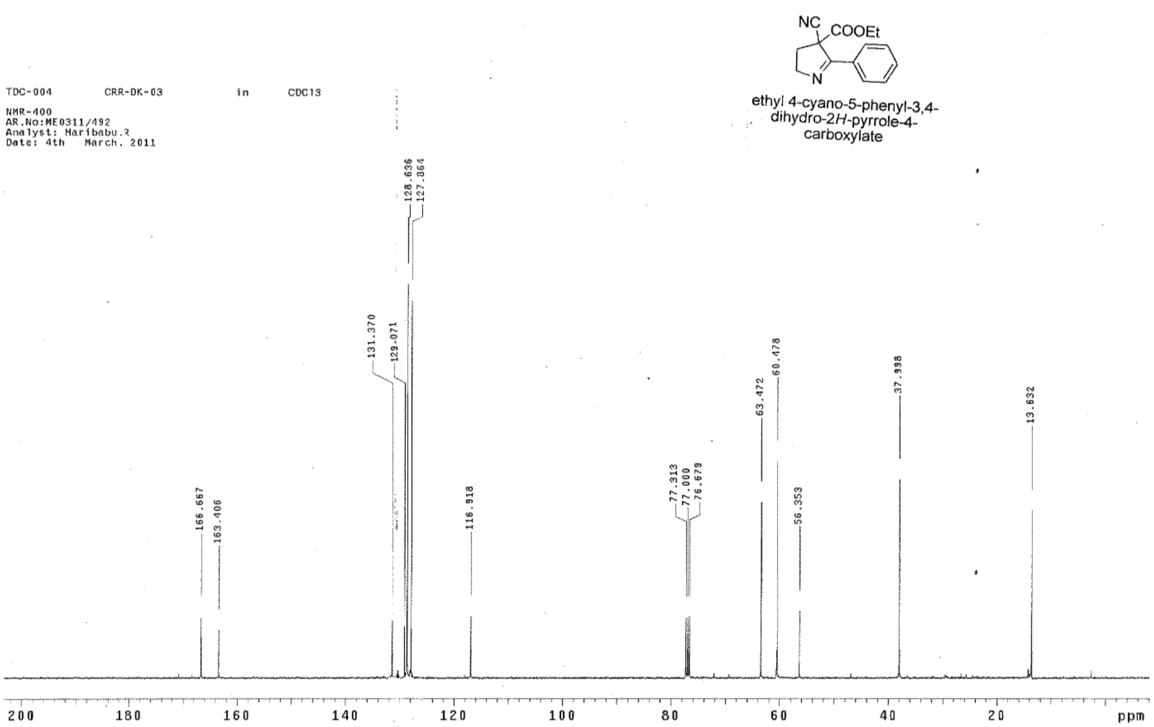

Mass spectrum of 29

CPS,MIYAPUR

Mass Analysis Report

**Data Filename**	110302007.d	**Sample Name**	2/SSM/113
**Sample Type**	Sample	**Position**	Vial 36
**Instrument Name**	Instrument 1	**User Name**	
**Acq Method**	ESI.m	**IRM Calibration Status**	Success
**DA Method**	CDD-MRM.m	**Comment**	

User Spectra

Fragmentor Voltage	Collision Energy	Ionization Mode
135	0	Esi

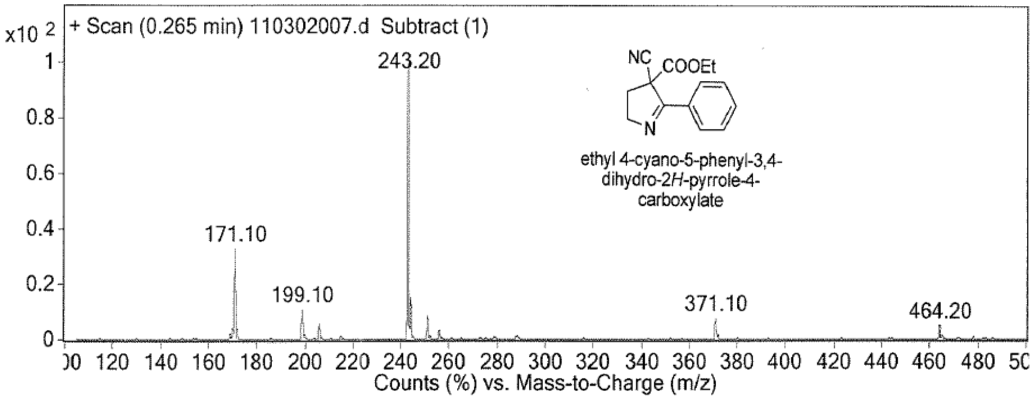

--- End Of Report ---

IR spectrum of 29

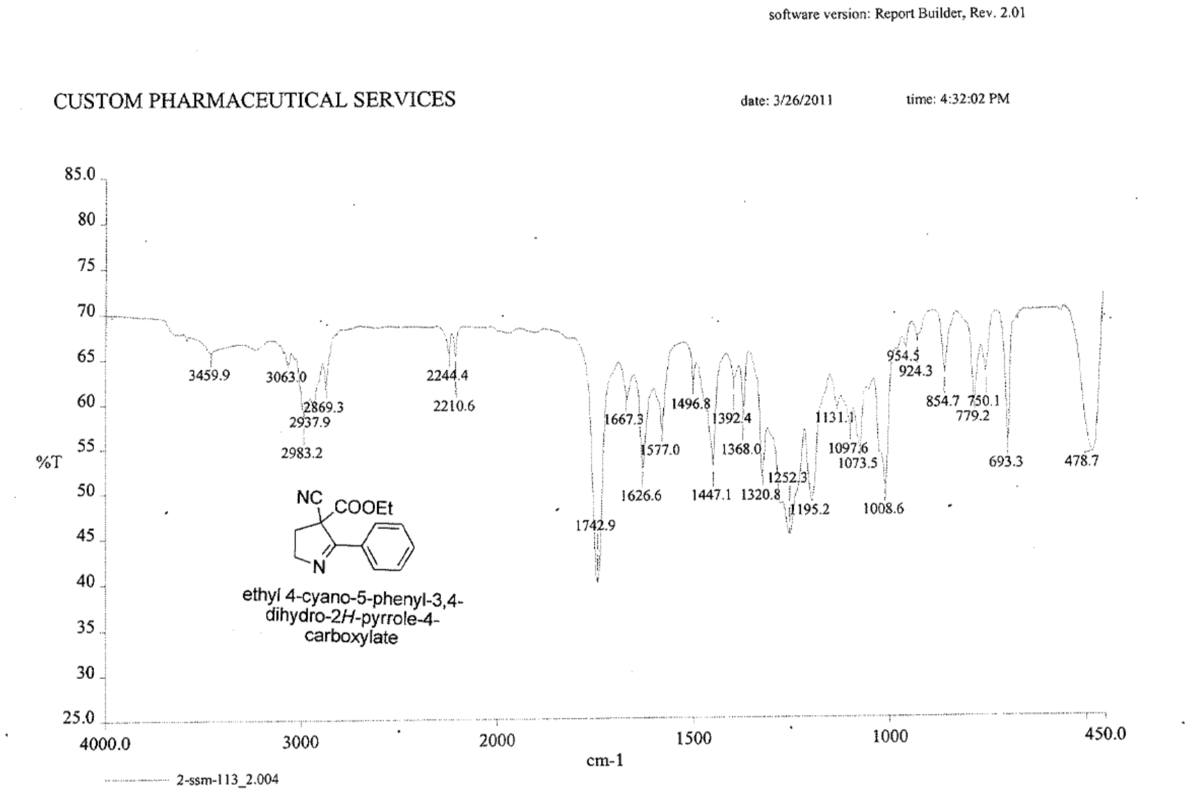

^{1}H NMR spectrum of 31

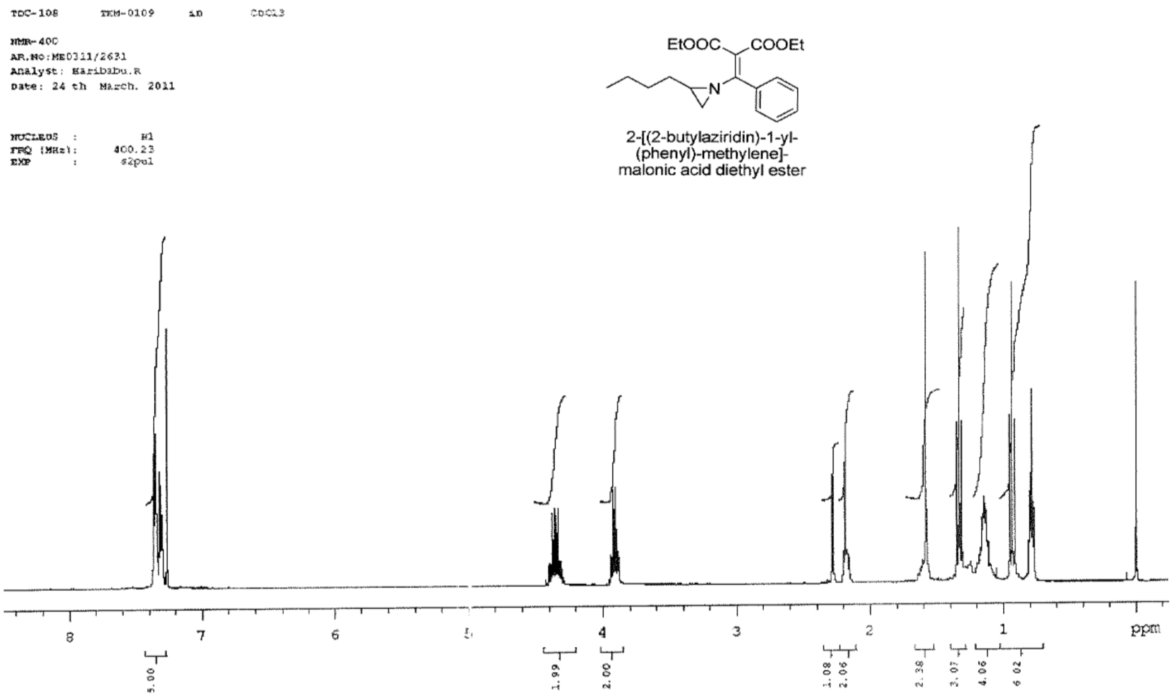

^{13}C NMR spectrum of 31

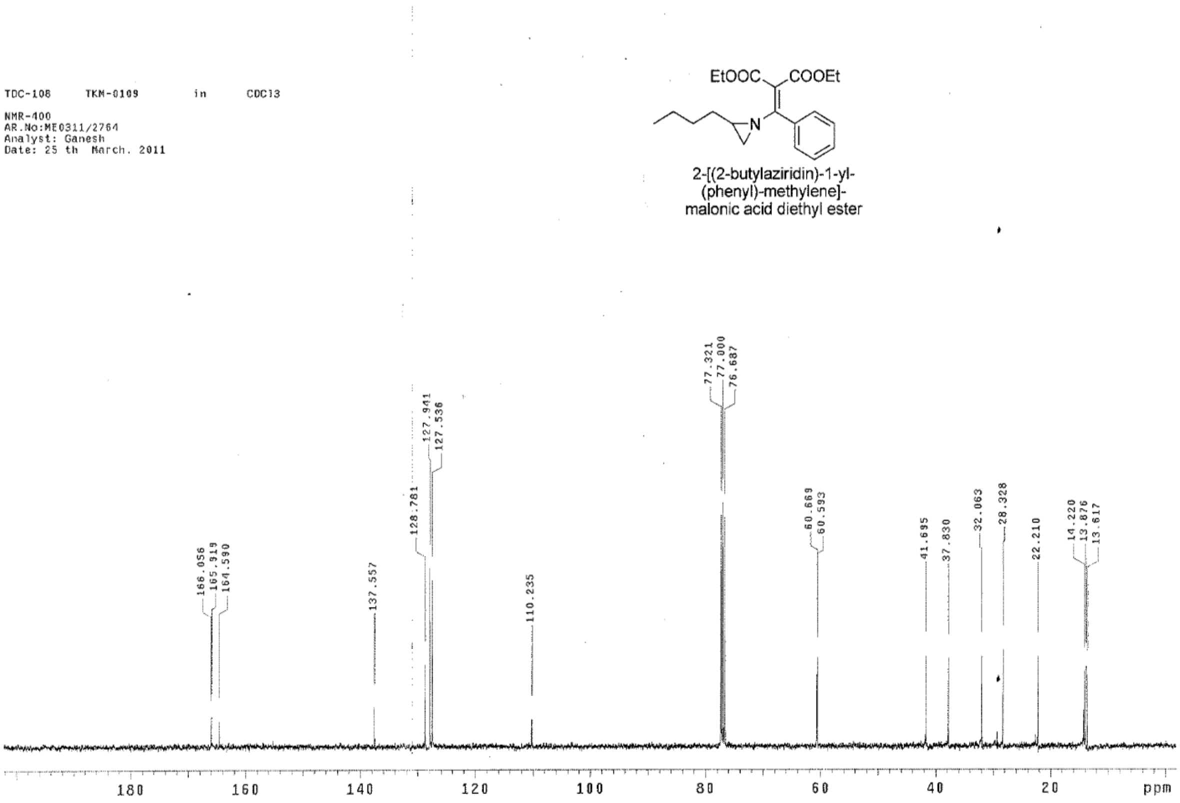

Mass spectrum of 31

CPS,MIYAPUR

Mass Analysis Report

**Data Filename**	110325005.d	**Sample Name**	TKM-0109
**Sample Type**	Sample	**Position**	Vial 18
**Instrument Name**	Instrument 1	**User Name**	
**Acq Method**	ESI.m	**IRM Calibration Status**	Success
**DA Method**	CEST1_QUANT.m	**Comment**	

User Spectra

Fragmentor Voltage	Collision Energy	Ionization Mode
135	0	Esi

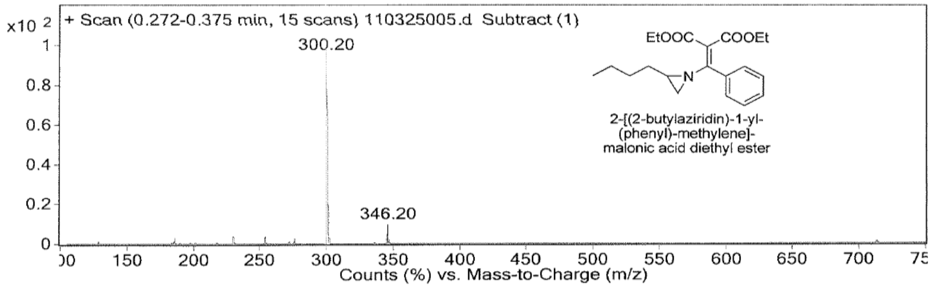

--- End Of Report ---

Page 1 of 1 Printed at: 5:25 PM on:3/25/2011

IR spectrum of 31

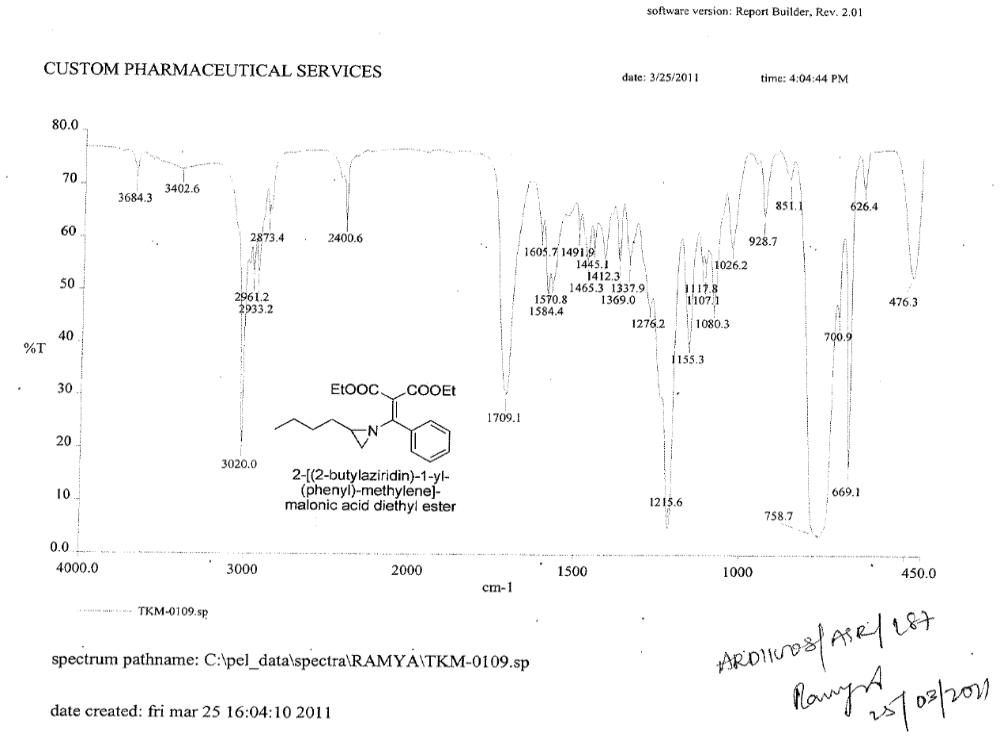

^{1}H NMR spectrum of 32

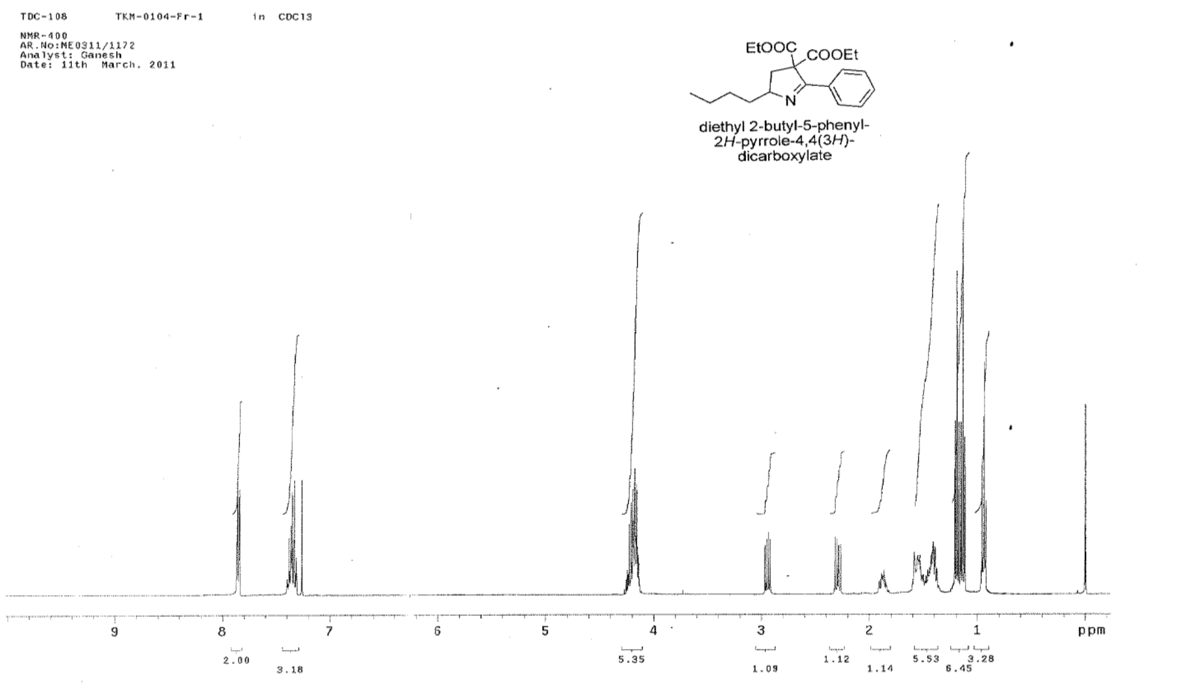

^{13}C NMR spectrum of 32

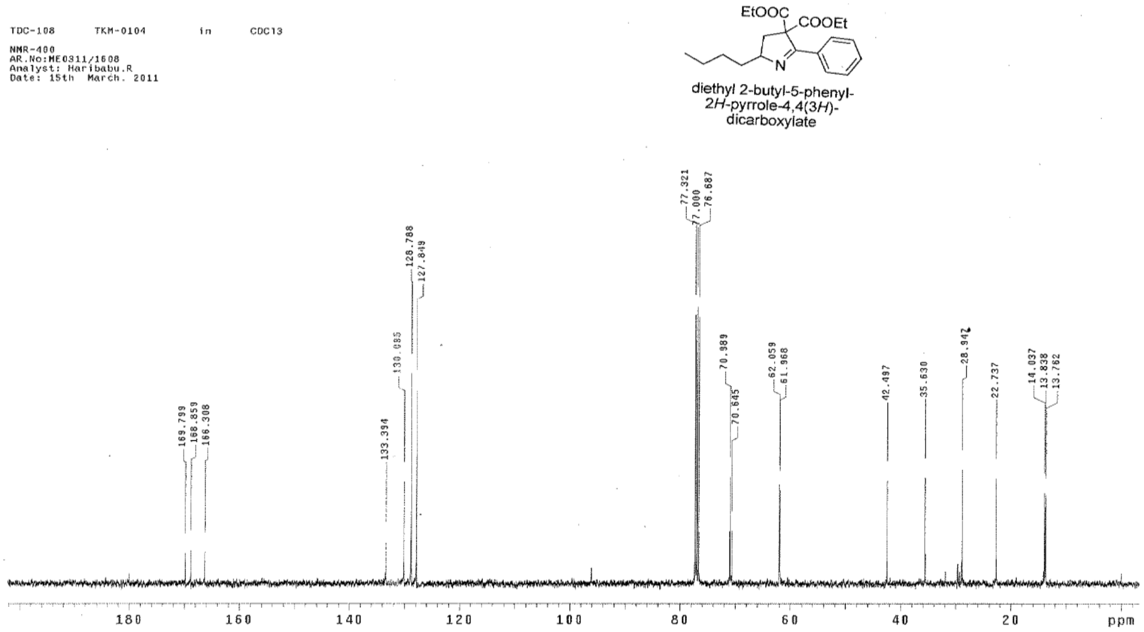

Mass spectrum of 32

CPS,MIYAPUR

Mass Analysis Report

**Data Filename**	110312026.d	**Sample Name**	TKM-0104/Fr1
**Sample Type**	Sample	**Position**	Vial 2
**Instrument Name**	Instrument 1	**User Name**	
**Acq Method**	ESI.m	**IRM Calibration Status**	Success
**DA Method**	CEST1_QUANT.m	**Comment**	

User Spectra

Fragmentor Voltage	Collision Energy	Ionization Mode
110	0	ESI

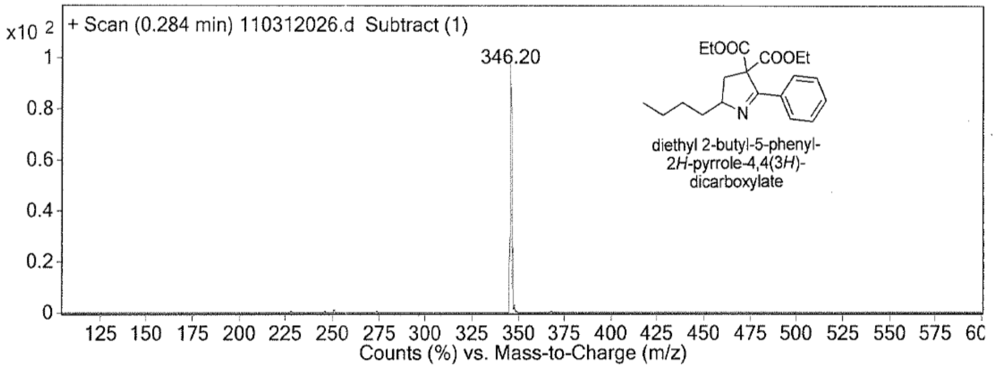

--- End Of Report ---

IR spectrum of 32

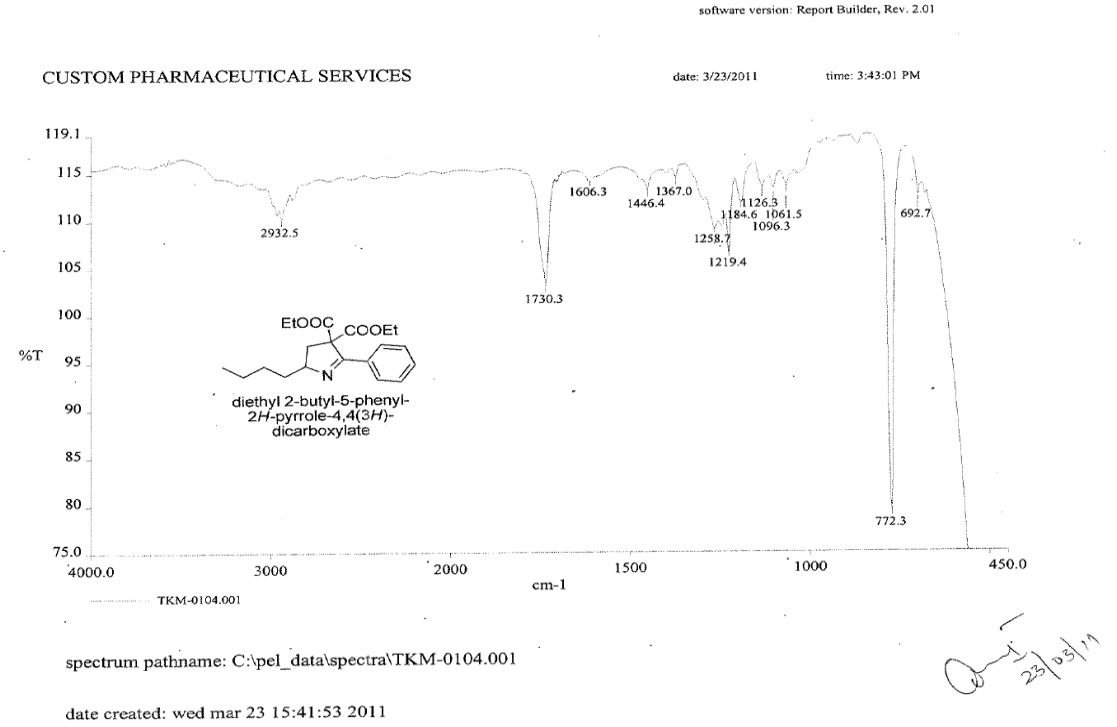

HRMS of 21a

Elemental Composition Report **Page 1**

Single Mass Analysis
Tolerance = 5.0 PPM / DBE: min = -1.0, max = 15.0
Element prediction: Off
Number of isotope peaks used for i-FIT = 3

Monoisotopic Mass, Even Electron Ions
52 formula(e) evaluated with 1 results within limits (all results (up to 1000) for each mass)
Elements Used:
C: 0-25 H: 0-25 N: 0-3 O: 0-5
TKM-070 TDC-108

Aurigene Discovery Technologies, Hyderabad

02/03/11

EtOOC COOEt

diethyl 5-methyl-2*H*-pyrrole-4,4(3*H*)-dicarboxylate

UT0311_014 22 (0.411) Cm (22:49-88:99x0.010)

02-Mar-2011
Lakshmikanth
1: TOF MS ES+
3.76e+005

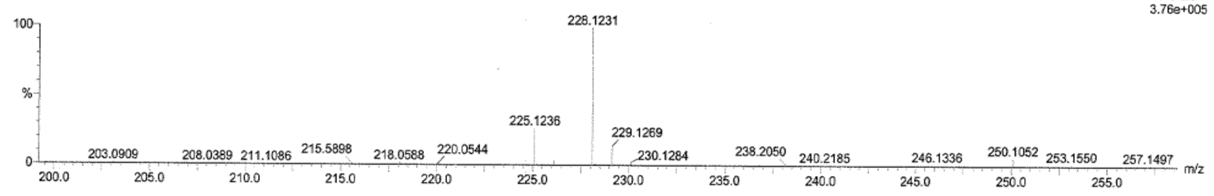

Minimum:				-1.0		
Maximum:		5.0	5.0	15.0		
Mass	Calc. Mass	mDa	PPM	DBE	i-FIT	Formula
228.1231	228.1236	-0.5	-2.2	3.5	102.7	C11 H18 N O4

HRMS of 21b

Elemental Composition Report

Page 1

Single Mass Analysis
Tolerance = 8.0 PPM / DBE: min = -1.0, max = 15.0
Element prediction: Off
Number of isotope peaks used for i-FIT = 3

Monoisotopic Mass, Even Electron Ions
28 formula(e) evaluated with 1 results within limits (all results (up to 1000) for each mass)
Elements Used:
C: 0-13 H: 0-30 N: 0-2 O: 0-5
TKM-046 TDC-108

02/03/11

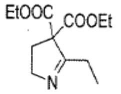

diethyl 5-ethyl-2*H*-pyrrole-4,4(3*H*)-dicarboxylate

Aurigene Discovery Technologies, Hyderabad

02-Mar-2011
Lakshmikanth
1: TOF MS ES+
2.87e+005

UT0311_015 15 (0.278) Cm (15:35-89:100x0.010)

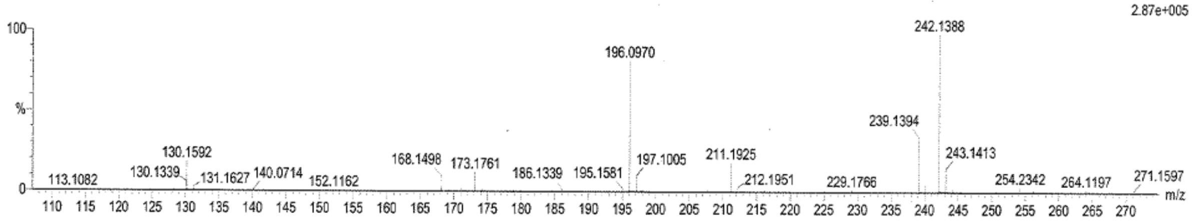

Minimum:				-1.0		
Maximum:		5.0	8.0	15.0		
Mass	Calc. Mass	mDa	PPM	DBE	i-FIT	Formula
242.1388	242.1392	-0.4	-1.7	3.5	148.4	C12 H20 N O4

HRMS of 21c

Elemental Composition Report

Page 1

Single Mass Analysis
Tolerance = 5.0 PPM / DBE: min = -1.0, max = 15.0
Element prediction: Off
Number of isotope peaks used for i-FIT = 3

Monoisotopic Mass, Even Electron Ions
62 formula(e) evaluated with 1 results within limits (all results (up to 1000) for each mass)
Elements Used:
C: 0-18 H: 0-30 N: 0-2 O: 0-8
TKM-031 TDC-108

02/03/11

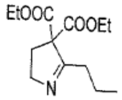

diethyl 5-propyl-2*H*-pyrrole-4,4(3*H*)-dicarboxylate

Aurigene Discovery Technologies, Hyderabad

02-Mar-2011
Lakshmikanth
1: TOF MS ES+
2.19e+005

UT0311_012 19 (0.351) Cm (19:31-80:88x0.010)

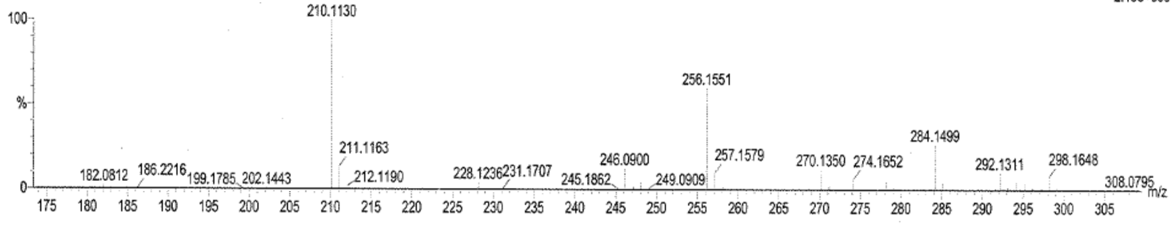

Minimum:				-1.0		
Maximum:		5.0	5.0	15.0		
Mass	Calc. Mass	mDa	PPM	DBE	i-FIT	Formula
256.1551	256.1549	0.2	0.8	3.5	139.4	C13 H22 N O4

HRMS of 21d

Elemental Composition Report Page 1

Single Mass Analysis
Tolerance = 5.0 PPM / DBE: min = -1.0, max = 15.0
Element prediction: Off
Number of isotope peaks used for i-FIT = 3

Monoisotopic Mass, Even Electron Ions
58 formula(e) evaluated with 1 results within limits (all results (up to 1000) for each mass)
Elements Used:
C: 0-18 H: 0-30 N: 0-2 O: 0-8
TKM-037 TDC-108

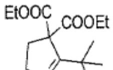

diethyl 5-*tert*-butyl-2*H*-pyrrole-4,4(3*H*)-dicarboxylate

Aurigene Discovery Technologies, Hyderabad

02-Mar-2011
Lakshmikanth
1: TOF MS ES+
3.29e+005

UT0311_011 29 (0.545) Cm (29:42-93:100x0.010)

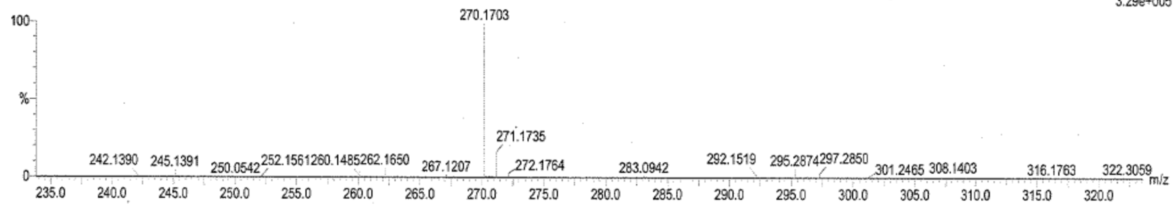

Minimum:				-1.0		
Maximum:		5.0	5.0	15.0		
Mass	Calc. Mass	mDa	PPM	DBE	i-FIT	Formula
270.1703	270.1705	-0.2	-0.7	3.5	149.3	C14 H24 N O4

HRMS of 21e

Elemental Composition Report Page 1

Single Mass Analysis
Tolerance = 5.0 PPM / DBE: min = -1.0, max = 15.0
Element prediction: Off
Number of isotope peaks used for i-FIT = 3

Monoisotopic Mass, Even Electron Ions
28 formula(e) evaluated with 1 results within limits (all results (up to 1000) for each mass)
Elements Used:
C: 0-18 H: 0-30 N: 0-2 O: 0-5
TKM-014 3 Amino piperdine CNOP

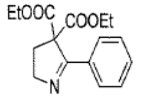

diethyl 5-phenyl-2*H*-pyrrole-4,4(3*H*)-dicarboxylate

Aurigene Discovery Technologies, Hyderabad

24-Feb-2011
Lakshmikanth
1: TOF MS ES+
3.40e+004

UT0211_183 16 (0.292) Cm (16:24-49:51x0.010)

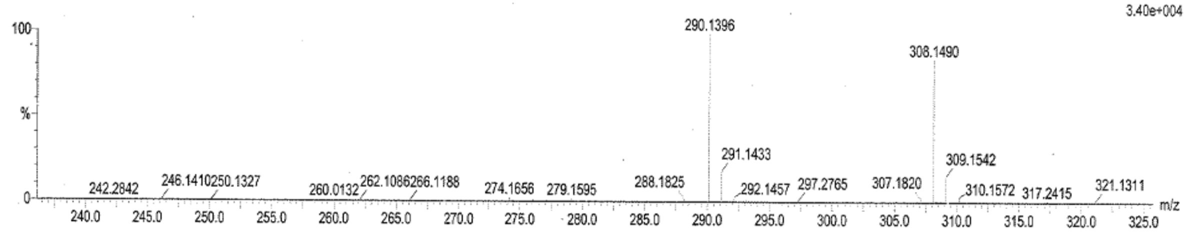

Minimum:				-1.0		
Maximum:		5.0	5.0	15.0		
Mass	Calc. Mass	mDa	PPM	DBE	i-FIT	Formula
290.1396	290.1392	0.4	1.4	7.5	3.2	C16 H20 N O4

HRMS of 21f

Elemental Composition Report

Page 1

Single Mass Analysis
Tolerance = 5.0 PPM / DBE: min = -1.0, max = 15.0
Element prediction: Off
Number of isotope peaks used for i-FIT = 3

Monoisotopic Mass, Even Electron Ions
38 formula(e) evaluated with 1 results within limits (all results (up to 1000) for each mass)
Elements Used:
C: 0-18 H: 0-30 N: 0-1 O: 0-6 Cl: 0-1
TKM-023 TDC-108

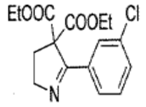

diethyl 5-(3-chlorophenyl)-2*H*-pyrrole-4,4(3*H*)-dicarboxylate

Aurigene Discovery Technologies, Hyderabad

02-Mar-2011
Lakshmikanth
1: TOF MS ES+
7.59e+005

UT0311_016 25 (0.472) Cm (25:40-93:100x0.010)

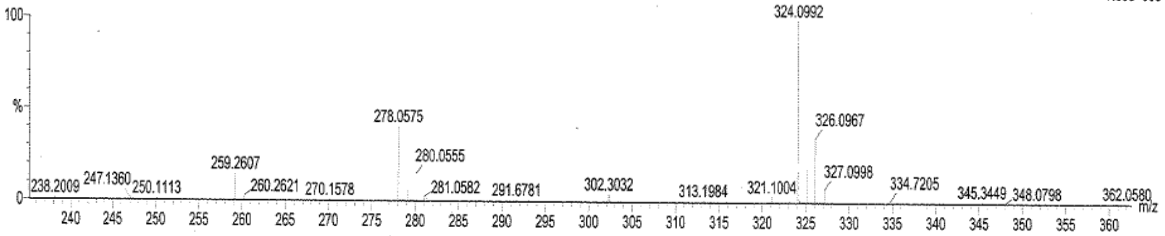

	Mass	Calc. Mass	mDa	PPM	DBE	i-FIT	Formula
Minimum:					-1.0		
Maximum:			5.0	5.0	15.0		
	324.0992	324.1003	-1.1	-3.4	7.5	75.0	C16 H19 N O4 Cl

HRMS of 21g

Elemental Composition Report

Page 1

Single Mass Analysis
Tolerance = 5.0 PPM / DBE: min = -1.0, max = 80.0
Element prediction: Off
Number of isotope peaks used for i-FIT = 4

Monoisotopic Mass, Even Electron Ions
34 formula(e) evaluated with 1 results within limits (up to 15 closest results for each mass)
Elements Used:
C: 0-18 H: 0-25 N: 0-1 O: 0-6 F: 0-1

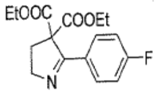

diethyl 5-(4-fluorophenyl)-2*H*-pyrrole-4,4(3*H*)-dicarboxylate

TKM-052 3 Amino piperdine CNOP

Aurigene Discovery Technologies, Hyderabad

24-Feb-2011
Lakshmikanth
1: TOF MS ES+
1.36e+004

UT0211_179 22 (0.412) Cm (21:28-86:100)

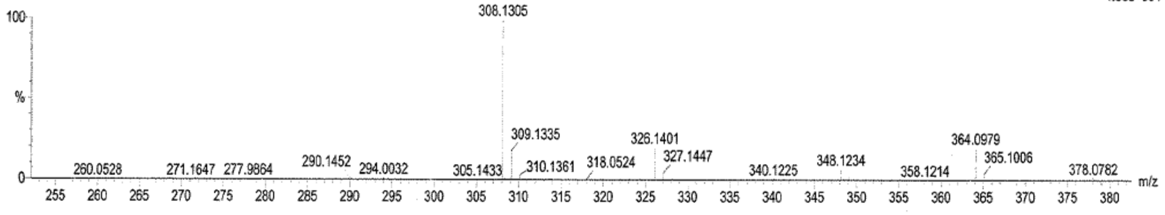

	Mass	Calc. Mass	mDa	PPM	DBE	i-FIT	Formula
Minimum:					-1.0		
Maximum:			5.0	5.0	80.0		
	308.1305	308.1298	0.7	2.3	7.5	5.2	C16 H19 N O4 F

HRMS of 21h

Elemental Composition Report

Page 1

02/03/11

Single Mass Analysis
Tolerance = 5.0 PPM / DBE: min = -1.0, max = 15.0
Element prediction: Off
Number of isotope peaks used for i-FIT = 3

Monoisotopic Mass, Even Electron Ions
38 formula(e) evaluated with 1 results within limits (all results (up to 1000) for each mass)
Elements Used:
C: 0-18 H: 0-30 N: 0-2 O: 0-8
TKM-055 3 Amino piperdine CNOP

Aurigene Discovery Technologies, Hyderabad

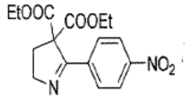

diethyl 5-(4-nitrophenyl)-2*H*-pyrrole-4,4(3*H*)-dicarboxylate

24-Feb-2011
Lakshmikanth
1: TOF MS ES+
2.40e+004

UT0211_180 17 (0.325) Cm (17:24-82:90x0.010)

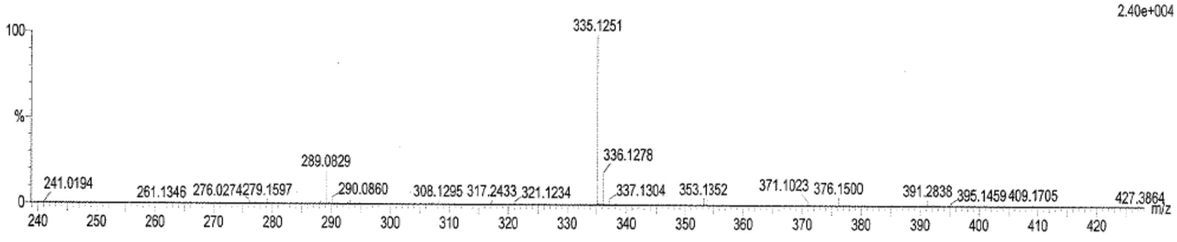

Minimum:				-1.0		
Maximum:		5.0	5.0	15.0		
Mass	Calc. Mass	mDa	PPM	DBE	i-FIT	Formula
335.1251	335.1243	0.8	2.4	8.5	4.5	C16 H19 N2 O6

HRMS of 21i

Elemental Composition Report

Page 1

02/03/11

Single Mass Analysis
Tolerance = 5.0 PPM / DBE: min = -1.0, max = 15.0
Element prediction: Off
Number of isotope peaks used for i-FIT = 3

Monoisotopic Mass, Even Electron Ions
42 formula(e) evaluated with 1 results within limits (all results (up to 1000) for each mass)
Elements Used:
C: 0-18 H: 0-30 N: 0-2 O: 0-8
TKM-058 TDC-108

Aurigene Discovery Technologies, Hyderabad

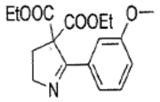

diethyl 5-(3-methoxyphenyl)-2*H*-pyrrole-4,4(3*H*)-dicarboxylate

02-Mar-2011
Lakshmikanth
1: TOF MS ES+
8.31e+004

UT0311_013 23 (0.426) Cm (23:28-89:93x0.010)

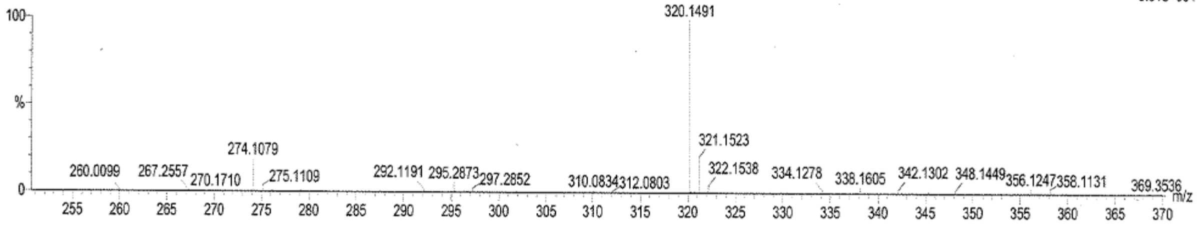

Minimum:				-1.0		
Maximum:		5.0	5.0	15.0		
Mass	Calc. Mass	mDa	PPM	DBE	i-FIT	Formula
320.1491	320.1498	-0.7	-2.2	7.5	90.1	C17 H22 N O5

HRMS of 21j

Elemental Composition Report **Page 1**

02/03/11

Single Mass Analysis
Tolerance = 5.0 PPM / DBE: min = -1.0, max = 15.0
Element prediction: Off
Number of isotope peaks used for i-FIT = 3

Monoisotopic Mass, Even Electron Ions
42 formula(e) evaluated with 1 results within limits (all results (up to 1000) for each mass)
Elements Used:
C: 0-15 H: 0-30 N: 0-2 O: 0-5 S: 0-1
TKM-017 TDC-108

Aurigene Discovery Technologies, Hyderabad

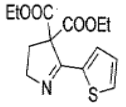

diethyl 5-(thiophen-2-yl)-2*H*-pyrrole-4,4(3*H*)-dicarboxylate

02-Mar-2011
Lakshmikanth
1: TOF MS ES+
7.55e+005

UT0311_010 19 (0.352) Cm (19:28-73:84x0.010)

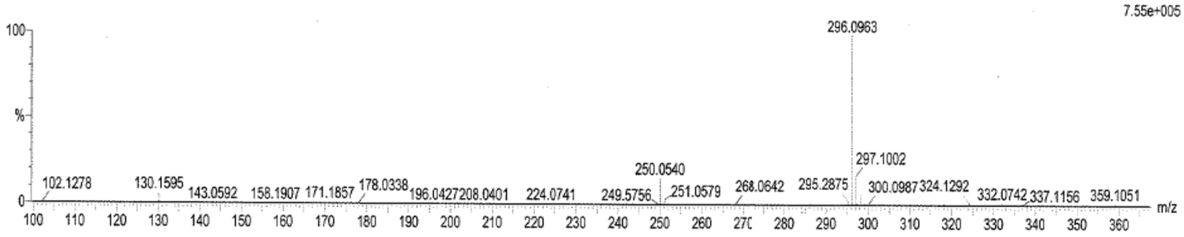

Minimum:				-1.0		
Maximum:		5.0	5.0	15.0		
Mass	Calc. Mass	mDa	PPM	DBE	i-FIT	Formula
296.0963	296.0957	0.6	2.0	6.5	125.1	C14 H18 N O4 S

HRMS of 23

Elemental Composition Report **Page 1**

Single Mass Analysis
Tolerance = 5.0 PPM / DBE: min = 0.0, max = 15.0
Element prediction: Off
Number of isotope peaks used for i-FIT = 3

Monoisotopic Mass, Even Electron Ions
32 formula(e) evaluated with 1 results within limits (all results (up to 1000) for each mass)
Elements Used:
C: 0-22 H: 0-35 N: 0-2 O: 0-5
2/SSM/1

Aurigene Discovery Technologies, Hyderabad

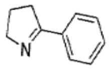

5-phenyl-3,4-dihydro-2*H*-pyrrole

25-Mar-2011
Lakshmikanth
1: TOF MS ES+
3.23e+005

UT0311_137 7 (0.251) Cm (5:9-47:50x0.010)

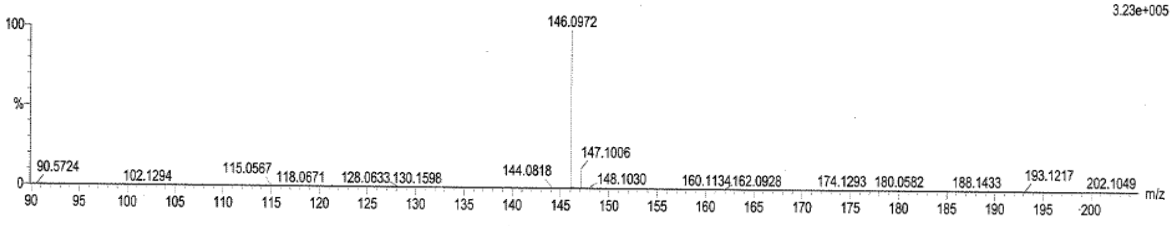

Minimum:				0.0		
Maximum:		5.0	5.0	15.0		
Mass	Calc. Mass	mDa	PPM	DBE	i-FIT	Formula
146.0972	146.0970	0.2	1.4	5.5	82.5	C10 H12 N

HRMS of 24

Elemental Composition Report **Page 1**

Single Mass Analysis
Tolerance = 8.0 PPM / DBE: min = 0.0, max = 15.0
Element prediction: Off
Number of isotope peaks used for i-FIT = 3

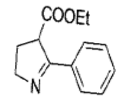

ethyl 5-phenyl-3,4-dihydro-2*H*-pyrrole-4-carboxylate

Monoisotopic Mass, Even Electron Ions
21 formula(e) evaluated with 1 results within limits (all results (up to 1000) for each mass)
Elements Used:
C: 0-15 H: 0-25 N: 0-2 O: 0-3
2/SSM/117

Aurigene Discovery Technologies, Hyderabad

23-Mar-2011
Lakshmikanth
1: TOF MS ES+
2.90e+004

UT0311_135 23 (0.620) Cm (23:28-53:55x0.010)

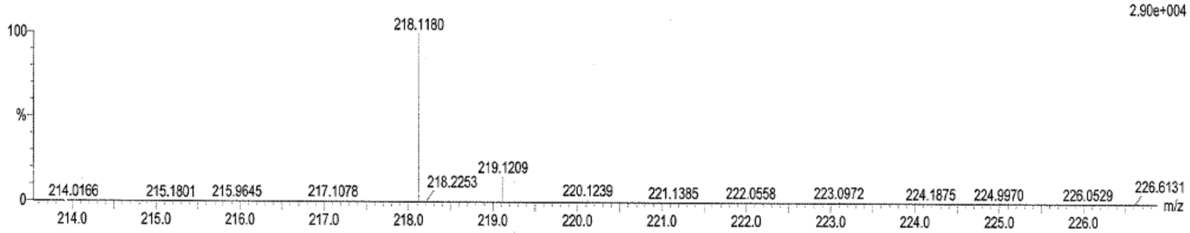

Minimum:				0.0		
Maximum:		5.0	8.0	15.0		
Mass	Calc. Mass	mDa	PPM	DBE	i-FIT	Formula
218.1180	218.1181	-0.1	-0.5	6.5	1.6	C13 H16 N O2

HRMS of 29

Elemental Composition Report **Page 1**

Single Mass Analysis
Tolerance = 5.0 PPM / DBE: min = -1.0, max = 15.0
Element prediction: Off
Number of isotope peaks used for i-FIT = 3

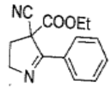

ethyl 4-cyano-5-phenyl-3,4-dihydro-2*H*-pyrrole-4-carboxylate

Monoisotopic Mass, Even Electron Ions
16 formula(e) evaluated with 1 results within limits (all results (up to 1000) for each mass)
Elements Used:
C: 0-15 H: 0-30 N: 0-3 O: 0-2
2/SSM/113 CATO

Aurigene Discovery Technologies, Hyderabad

02-Mar-2011
Lakshmikanth
1: TOF MS ES+
7.41e+005

UT0311_037 21 (0.397) Cm (21:31-89:94x0.010)

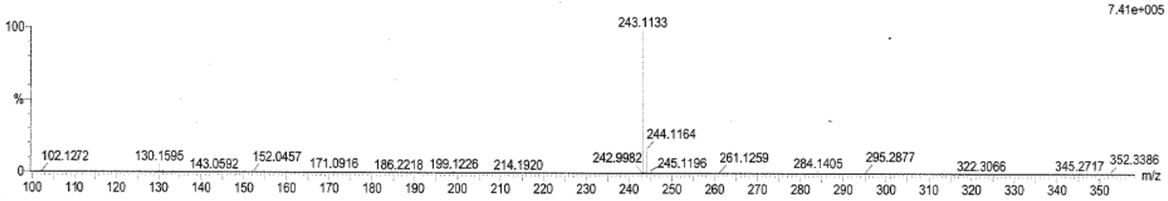

Minimum:				-1.0		
Maximum:		5.0	5.0	15.0		
Mass	Calc. Mass	mDa	PPM	DBE	i-FIT	Formula
243.1133	243.1134	-0.1	-0.4	8.5	71.1	C14 H15 N2 O2

HRMS of 32

Elemental Composition Report Page 1

Single Mass Analysis
Tolerance = 5.0 PPM / DBE: min = -1.0, max = 15.0
Element prediction: Off
Number of isotope peaks used for i-FIT = 3

14/03/11

Monoisotopic Mass, Even Electron Ions
178 formula(e) evaluated with 1 results within limits (all results (up to 1000) for each mass)
Elements Used:
C: 0-25 H: 0-35 N: 0-2 O: 0-6 S: 0-2

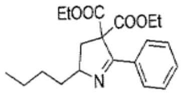

diethyl 2-butyl-5-phenyl-2*H*-pyrrole-4,4(3*H*)-dicarboxylate

TKM-0104 Aurigene Discovery Technologies, Hyderabad 14-Mar-2011
Lakshmikanth
UT0311_082 21 (0.489) Cm (21:31-64:72x0.010) 1: TOF MS ES+
5.95e+005

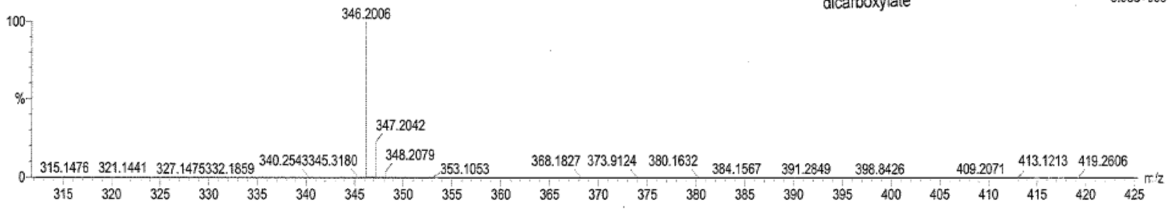

Minimum:				-1.0		
Maximum:		5.0	5.0	15.0		
Mass	Calc. Mass	mDa	PPM	DBE	i-FIT	Formula
346.2006	346.2018	-1.2	-3.5	7.5	162.1	C20 H28 N O4
